# Principal component analysis based unsupervised feature extraction applied to budding yeast temporally periodic gene expression

**DOI:** 10.1186/s13040-016-0101-9

**Published:** 2016-06-29

**Authors:** Y-h Taguchi

**Affiliations:** Department of Physics, Chuo University, 1-13-27 Kasuga, Bunkyo-ku, Tokyo, 112-8551 Japan

**Keywords:** Principal component analysis, Feature extraction, Budding yeast, Cell division cycle, Gene expression

## Abstract

**Background:**

The recently proposed principal component analysis (PCA) based unsupervised feature extraction (FE) has successfully been applied to various bioinformatics problems ranging from biomarker identification to the screening of disease causing genes using gene expression/epigenetic profiles. However, the conditions required for its successful use and the mechanisms involved in how it outperforms other supervised methods is unknown, because PCA based unsupervised FE has only been applied to challenging (i.e. not well known) problems.

**Results:**

In this study, PCA based unsupervised FE was applied to an extensively studied organism, i.e., budding yeast. When applied to two gene expression profiles expected to be temporally periodic, yeast metabolic cycle (YMC) and yeast cell division cycle (YCDC), PCA based unsupervised FE outperformed simple but powerful conventional methods, with sinusoidal fitting with regards to several aspects: (i) feasible biological term enrichment without assuming periodicity for YMC; (ii) identification of periodic profiles whose period was half as long as the cell division cycle for YMC; and (iii) the identification of no more than 37 genes associated with the enrichment of biological terms related to cell division cycle for the integrated analysis of seven YCDC profiles, for which sinusoidal fittings failed. The explantation for differences between methods used and the necessary conditions required were determined by comparing PCA based unsupervised FE with fittings to various periodic (artificial, thus pre-defined) profiles. Furthermore, four popular unsupervised clustering algorithms applied to YMC were not as successful as PCA based unsupervised FE.

**Conclusions:**

PCA based unsupervised FE is a useful and effective unsupervised method to investigate YMC and YCDC. This study identified why the unsupervised method without pre-judged criteria outperformed supervised methods requiring human defined criteria.

**Electronic supplementary material:**

The online version of this article (doi:10.1186/s13040-016-0101-9) contains supplementary material, which is available to authorized users.

## Background

Small-sample-large-feature problems, which occur when limited numbers of samples are available despite a large number of associated features, are common when biomedical/genomic data sets are analyzed. This is because the number of features is often equal to or greater than the number of genes (i.e., tens of thousands), whereas the number of samples are usually as small as the number of patients (in vivo study) or cell lines (in vitro study), i.e. a few hundred but often fewer than ten. The recently proposed principal component analysis (PCA) based unsupervised feature extraction (FE) [1–12] is an effective method to overcome these difficulties. Previously, PCA based unsupervised FE successfully identified stable (relatively insensitive to sample selection) sets composed of limited numbers of circulating microRNA that discriminated between multiple diseases (putative universal disease biomarkers), genes associated with aberrant promoter methylation commonly found among three distinct autoimmune diseases by integrating promoter methylation profiles from three distinct autoimmune diseases, and candidate disease-causing genes ranging from cancers to neurodegenerative diseases by integrating distinct expression profiles (genomic data and DNA methylation, mRNA and miRNA profiles, mRNA expression and promoter methylation). Despite several successful studies, the use of this methodology is not widely supported, possibly because no criteria regarding its successful use and the mechanisms involved in how it outperforms other methods have been reported. This lack of knowledge is because PCA based unsupervised FE was previously applied to challenging problems that other conventional methods cannot deal with to demonstrate superiority to existing methods. Without a comparison of results, the reasons why PCA based unsupervised FE can outperform other conventional methods cannot be determined.

In this study, we applied PCA based unsupervised FE to a well-established and extensively studied problem; namely the identification of *Saccharomyces cerevisiae* genes that exhibit temporal periodic expression. Because budding yeast genes have been ascribed well-defined functions to a greater degree than for other organisms, the suitability of genes identified by PCA based unsupervised FE can be evaluated. Specifically, two kinds of gene expression profiles measured under distinct conditions - yeast metabolic cycle (YMC) and yeast cell division cycle (YCDC) - were analyzed such that evaluations made were not strictly dependent upon the specific example. We found that fitting to the assumed functions including frequently employed sinusoidal functions is often erroneous and this might explain why conventional and supervised methods are often outperformed by unsupervised methodologies that do not assume the length of period as well as functional forms to be fitted. This also generally demonstrates the disadvantage of employing model-based methodologies because they are popular or commonly used. To our knowledge, this is the first successful unsupervised identification of budding yeast genes that exhibit temporal periodicity without specifying the length of period or accessing the information of known (previously reported) cell cycle regulated genes.

## Results

### PCA based unsupervised FE applied to yeast metabolic cycle

**Fig. 1:**
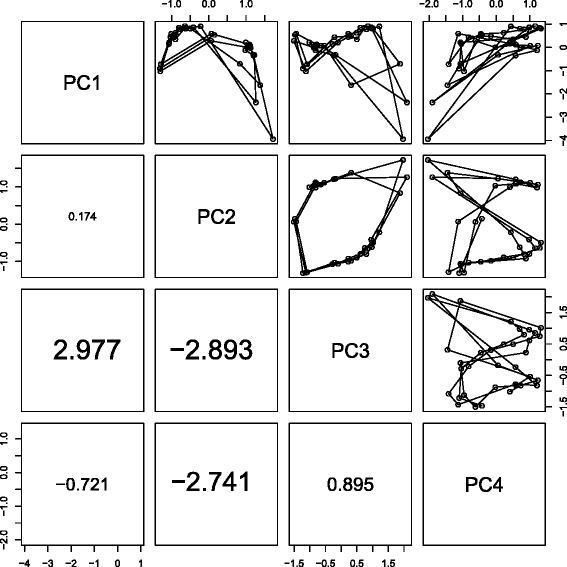
*Upper triangle*: scatter plots in YMC between the *k*th (1≤*k*≤4) PC loadings, *v*_*kj*_−〈*v*_*kj*_〉_*j*_,1≤*j*≤*M*, where *M* is the number of time points. *Lower triangle*: winding number *W*(*M*−1); *W*(*M*−1) at the *k*th row and *k* ^′^(<*k*)th column correspond to the scatter plot at the symmetric position, i.e., at the *k* ^′^th row and *k*th column. Time dependence of PC2 and PC3 loadings are shown in Fig. 3 b. The contributions of PC loadings are 88.7, 6.2, 2.1 and 0.6 %, respectively, indicating that PC loadings,*v*_*kj*_,*k*=2,3, with contributions as small as a few percent that correspond to tens of genes when the total number of genes correspond to thousands, cannot be disregarded

**Fig. 2:**
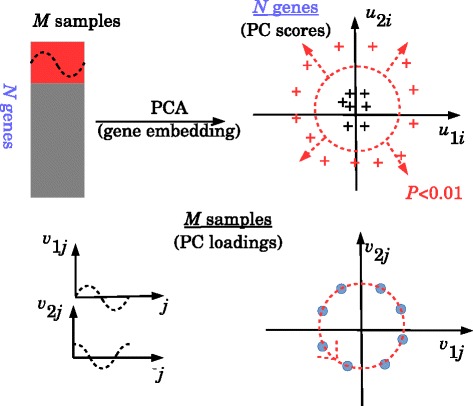
Schematic of PCA based unsupervised FE. In gene expression analyses (*top left*), limited genes (*red*) exhibit periodic motion. After gene embedding (*top right*), genes exhibiting periodic motion (*red crosses*) are identified as outliers if the BH criterion adjusted *P*-values are lower than 0.01 (or 0.05) assuming that the PC score attributed to each gene obeys Gaussian distribution. This is because PC loading attributed to each sample exhibits periodic motion (*bottom*)

PCA based unsupervised FE was applied to temporal gene expression observed during YMC [13] (see Methods). To identify principal component (PC) loadings that exhibited limit cycles, winding number analysis (see Methods) was applied. Figure 1 shows the identification of winding numbers and scatter plots of PC loadings. Because the first four PC loadings exhibited limit cycles when combined with any of the other four, the four PCs were used for PCA based unsupervised FE (see Methods and Fig. 2). The list of genes identified by PCA based unsupervised FE is shown in Additional file 1: Table S1A.

**Fig. 3:**
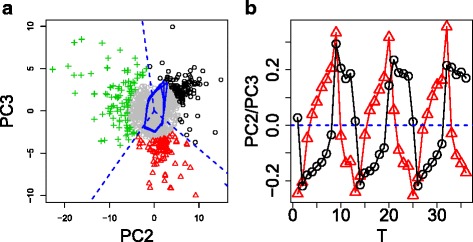
**a** Two dimensional embeddings of gene expression in YMC using PC2 and PC3 scores *u*_*kj*_,*k*=2,3, (the list of genes is shown in Additional file 1: Table S1B). *Black circle*, *red triangles*, and *green crosses* represent genes extracted and *gray circles* represent other genes. Biological terms enriched in genes shown as *black circles*, *red triangles* and *green crosses* are available in columns Cluster_1, Cluster_2, and Cluster_3 in Additional file 4: Table S3, respectively. Colors correspond to clusters identified by K-means. *Solid blue lines* represent PC2 and PC3 loadings and *broken blue lines* represent the boundary between the three clusters. **b** Time dependence of PC2 (*black*) and PC3 (*red*) loadings *u*_*kj*_,*k*=2,3

To identify the biological significance of the identified genes, the identified genes were uploaded to g:profiler [14], an enrichment analysis server. Although the full list of enriched Gene Ontology (GO) terms and pathways is available in Additional file 2: Table S2A, some specific examples are discussed below.

Among the identified significant enrichments of 138 GO Biological Process (BP) terms, 125 were child terms of “metabolic process”, which demonstrated the suitability of the methodology, because YMC was studied. Furthermore, most of the 46 significantly enriched GO Cellular Component (CC) terms were related to either ribosomes or mitochondria, both of which were also reported to be significant in a previous original research study [13]. Twenty-five GO Molecular Function (MF) terms and 13 Kyoto Encyclopedia of Genes and Genomes (KEGG) pathways were significantly enriched including reasonable pathways, such as “TCA cycle”, “Ribosome” and “metabolic pathways”. Twenty-one significantly enriched REACTOME pathways were mainly related to metabolism. All of these enrichments suggested the successful identification of critical genes in YMC using PCA based unsupervised FE without specifying the length of period. To our knowledge, this is the first successful identification of cell cycle regulated genes without using the length of period or accessing the information of known (previously reported) cell cycle regulated genes.

Tu et al. [13] also tried to group genes according to time points that exhibited peaks and found that distinct biological functions were attributed to three groups of genes, which were also automatically detected by our methodology as shown below. Because the period of cell division cycle was expected to equal the longest period observed, PC2 and PC3 (Fig. 1) were expected to represent the limit cycle corresponding to the cell division cycle Tu *et al* identified. Then, PCA based unsupervised FE was applied using only PC2 and PC3 (the list of genes is shown in Additional file 1: Table S1B). Figure 3 shows two-dimensional embeddings of the identified genes onto the plane spanned by PC2 and PC3 scores (limit cycle composed of PC2 and PC3 loadings is overdrawn). Clustering genes to three clusters using K-means (see Methods) was used to identify the three well-separated clusters (list of genes in each cluster is shown in Additional file 3: Document S1; black circles, red triangles and green crosses in Fig. 3 correspond to clusters 1, 2 and 3 in Additional file 3: Document S1, respectively). These clusters were clearly divided by angular variables (broken blue lines) despite the K-means not clustering genes apart from the angular variables, but with two-dimensional Cartesian coordinates. This suggested that PCA based unsupervised FE successfully identified three clusters coincident with phase variables during cell division cycles in an unsupervised manner without specifying the length of period. This demonstrates the superiority of PCA based unsupervised FE over other methods.

To confirm the superiority of PCA based unsupervised FE, we separately uploaded three groups of genes to g:profiler (Additional file 4: Table S3). These groups represented three distinct biological functions - ribosomes, mitochondria, and cell division - which were identified [13] as three functional groups assigned to three groups of genes. Thus, PCA based unsupervised FE without specifying the length of cell cycle period successfully identified the three functional gene groups identified by Tu et al. after their sophisticated and careful inspection of gene expression. Thus, PCA based unsupervised FE identified gene expression similar to supervised methods.

Moreover, PCA based unsupervised FE identified genes associated by periodic motion whose length of period was half of the cell cycle, because some orbits exhibited a figure eight shaped closed loop rather than a circle (Fig. 1). As long as the assumed temporal periodicity is strictly coincident with cell division cycle (i.e., the length of period must be as long as the cell division cycle), it is impossible to identify genes associated with periodic motion whose length of period is half of the cell cycle. Because genes including those associated with periodic motion whose length of period is half of the cell cycle, are fully associated with the enrichment of various biological terms (Additional file 2: Table S2), it is critically important to identify these genes. However, they cannot be identified when using supervised methodology coincident with cell division cycle. Thus, PCA based unsupervised FE not only can reproduce the outcomes identified by supervised methodology (Fig. 3 and Additional file 4: Table S3), but can also identify additional sets of cell cycle regulated genes that cannot be identified by supervised methods. Thus, PCA based unsupervised FE clearly outperformed the supervised methodology.

We also investigated protein-protein interactions (PPI) among genes identified by PCA based unsupervised FE using PC1, PC2, PC3 and PC4 (genes listed in Additional file 1: Table S1A). We uploaded the list of genes to the STRING server [15], which integrates various pairwise interactions between proteins. PPI enrichment estimated by STRING, which identified 419 genes among 422 uploaded genes, was 12,525, compared with the expected number, 5.50×10^3^(*P*=0). Thus, there was highly significant PPI enrichment between the selected genes.

### PCA based unsupervised FE applied to yeast cell division cycle

**Fig. 4:**
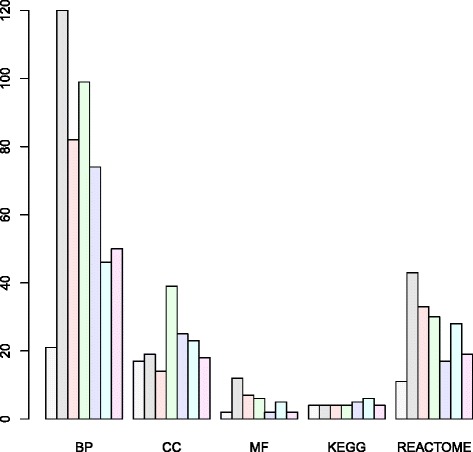
Number of biological terms enriched in each experiment (YCDC [16]). Key for color bars and individual experiments: *gray bars* correspond to Additional file 5: Figure S1A, Additional file 1: Table S1C and Additional file 6: Table S4A, *black bars* correspond to Additional file 5: Figure S1B, Additional file 1: Table S1D and Additional file 6: Table S4B, *magenta bars* correspond to Additional file 5: Figure S1C, Additional file 1: Table S1E and Additional file 6: Table S4C, *green bars* correspond to Additional file 5: Table Figure S1D, Additional file 1: Table S1F and Additional file 6: Table S4D, *blue bars* correspond to Additional file 5: Figure S1E, Additional file 1: Table S1G and Additional file 6: Table S4E, *cyan bars* correspond to Additional file 5: Figure S1F, Additional file 1: Table S1H and Additional file 6: Table S4F, and *pink bars* correspond to Additional file 5: Figure S1G, Additional file 1: Table S1I and Additional file 6: Table S4G

Although PCA based unsupervised FE was successfully applied to YMC, we confirmed its usefulness using another example, YCDC. Although YCDC is a yeast biological process that exhibits temporally periodic oscillations of gene expression, in contrast to the self-induced nature of YMC, YCDC is initiated from the artificially arrested G1 state; thus it is expected to differ from YMC. PCA based unsupervised FE was applied to seven of eight gene expression profiles in cyclebase [16], which ranks genes based upon both periodicity and the amplitude of gene expression. One set of data [17] was excluded because de Lichtenberg et al. pre-screened genes based upon previous studies. Because PCA based unsupervised FE screens significant genes as outliers, it does not function without the inclusion of non-outliers (seemingly non-significant) genes. Winding number analysis was applied and a pair of PCs that exhibited limit cycle were identified (Additional file 5: Figure S1). PCA based unsupervised FE identified more than 100 genes for each profile (list of genes shown in Additional file 1: Table S1C to S1I). Identified genes were uploaded to g:profiler independently (Additional file 6: Table S4, columns A to G correspond to Additional file 5: Figure S1A to S1G). A large number of identified biological terms were significantly enriched (Fig. 4) and were specifically related to cell division cycle, e.g., “cell cycle”, “cell cycle process”, “cell cycle phase transition”, “mitotic cell cycle”, “mitotic cell cycle process”, “DNA metabolic process”, “DNA repair”, (GO BP terms enriched in all seven experiments), “protein-DNA complexes”, “replication fork”, “nuclear replication fork”, (GO CC terms enriched in all seven experiments) “cyclin-dependent protein serine/threonine kinase regulatory activity” (a GO MF term enriched in all seven experiments), “missmatch repair”, “cell cycle - yeast”, “DNA replication” (KEGG pathways enriched in all seven experiments), “cell cycle”, “mitotic G1 - G1/S phases”, “mitotic G2 - G2/M phases”, (REACTOME pathways enriched in all seven experiments), and MCM1 and MCM1+SFF TF motifs (enriched in all seven experiments). *Fkh2* was previously recognized as a critical component of the MCM1-SFF complex for the regulation of cell cycle-dependent gene expression [18] and regulates the cell division cycle of *Schizosaccharomyces pombe* [19]. Thus, PCA based unsupervised FE identified many biological terms specific to the cell division cycle for all seven experiments.

We also investigated PPIs among the selected genes. We uploaded the list of genes in Additional file 1: Table S1C (those identified by applying PCA based unsupervised FE to the cdc28-13 cell experiments described by Cho et al. [20]) to the STRING server [15]. Although it was associated with the least number of enrichments (Fig. 4, gray bars), the number of PPI identified by STRING in 140 genes identified among 141 uploaded genes was 674, compared with the expected number, 2.83×10^2^(*P*=0). Thus, there was a highly significant PPI enrichment between the selected genes.

### Integration of YCDC gene expression using PCA based unsupervised FE

#### PCA based unsupervised FE identified common genes over seven experiments

**Fig. 5:**
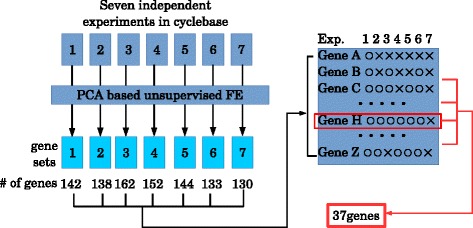
Schematic of integrated analysis of YCDC by PCA based unsupervised FE. After PCA based unsupervised FE was applied to each of seven experiments, seven sets of genes were identified. Then, the frequency of each gene identified within the seven sets of genes was counted. Thirty-seven genes were associated with more than or equal to six counts

Although we demonstrated PCA based unsupervised FE was successful for YMC and YCDC, the most important advantage compared with conventional (supervised) FE was integration. Because cyclebase is the integrated analysis of multiple cell division cycle gene expressions, it is the correct target for comparisons with integrated analysis using PCA based unsupervised FE. For integrated analyses by PCA based unsupervised FE, we used genes commonly selected among the seven experiments (see Fig. 5). Thirty-seven genes were identified in six or more of the seven experiments (list of genes is shown in Additional file 1: Table S1J). This was remarkable, because several hundred genes were selected from each experiment, which included several thousand genes. The probability that as many as 37 genes were accidentally identified in six of seven independent experiments is extremely small.

#### Enrichment analyses via YeastMine and g:profiler

Although this suggested that integrated analysis using PCA based unsupervised FE was successful, to verify further the biological feasibilities of the 37 identified genes, we uploaded these genes and 36 of the top ranked genes in cyclebase (list of genes is shown in Additional file 1: Table S1K) to the enrichment server, g:profiler (Additional file 7: Table S5). Performance in GO BP enrichment was comparable between PCA based unsupervised FE and cyclebase. The most important GO terms, e.g., “cell cycle”, “cell cycle process”, “mitotic cell cycle”, “mitotic cell cycle process”, and “DNA repair” were shared between PCA based unsupervised FE and cyclebase, although the number of genes identified in each term were greater in PCA based unsupervised FE than in cyclebase and “cell division” and “reproduction” were enriched only in genes identified by PCA based supervised FE. Although genes extracted from cyclebase had a greater enrichment of CC GO terms than PCA based unsupervised FE, the number of critical GO terms was comparative: “cellular bud” and “cellular bud neck” were enriched in genes identified by PCA based unsupervised FE while “replication fork” and “nuclear replication fork” were enriched in genes extracted from cyclebase. Enrichment in GO MF terms was greater in genes identified by PCA based unsupervised FE because “cyclin-dependent protein serine/threonine kinase regulator activity” was enriched while “protein heterodimerization” was the only GO MF term enriched in genes extracted from cyclebase. Although two KEGG pathway terms “cell cycle - yeast” and “missmatch repair” were enriched in genes extracted from cyclebase and PCA based unsupervised FE, the number of genes included in both KEGG pathways were greater in genes identified by PCA based unsupervised FE. The REACTOME pathway exhibited the biggest distinction - many REACTOME pathways specifically related to cell division cycle; e.g., “M phase”, “mitotic G1- G1/S phases”, “mitotic G2 - G2/M phases”, and “regulation of mitotic cell cycle” were enriched in genes identified by PCA based unsupervised FE, while the only REACTOME pathways enriched in genes extracted from cyclebase were polymerase related pathways. Although PCA based unsupervised FE correctly detected the enrichment of two TFs, MCM1 and SFF (see above), cyclebase identified only one TF, STE11, which has not been previously reported to be directly related to cell division cycle, although the enrichment of TF targeting was even originally employed to demonstrate the superiority of cyclebase over other data bases [21]. Overall, the performance by PCA based unsupervised FE outperformed cyclebase.

To confirm the superiority of PCA based unsupervised FE over cyclebase, genes were uploaded to an alternative enrichment server, YeastMine [22] (full list of results is shown in Additional file 8: Table S6). YeastMine was employed as well as g:profiler because it specifically targets yeasts. Thus, slight differences missed by g:profiler might be detected by YeastMine. Table 1 shows the top five GO BP terms/publications for both gene sets. As expected, YeastMine reported a clear superiority of PCA based unsupervised FE over cyclebase. GO BP terms enriched in genes identified by PCA based unsupervised FE were directly related to the cell division cycle, whereas genes from cyclebase were not. For publication enrichment, PCA based unsupervised FE outperformed cyclebase, because the top ranked publication for genes identified by PCA based unsupervised FE included 20 genes and studied cell division cycle while the genes identified by cyclebase only included seven genes and did not directly study cell division cycle.

**Table 1:** Top five GO BP term/publication enrichments reported by YeastMine [22] in genes identified by either PCA based unsupervised FE or cyclebase

PCA based unsupervised FE				Cyclebase			
GO BP Term		*p*-Value	#	GO BP Term		*p*-Value	#
Cell cycle	[GO:0007049]	5.32E-10	24	Chromosome organization	[GO:0051276]	1.13E-8	20
Cell cycle process	[GO:0022402]	3.08E-8	21	Telomere maintenance via recombination	[GO:0000722]	3.34E-8	8
Mitotic cell cycle	[GO:0000278]	4.45E-8	17	DNA metabolic process	[GO:0006259]	3.50E-8	19
Mitotic cell cycle process	[GO:1903047]	2.23E-7	16	Telomere maintenance	[GO:0000723]	2.07E-6	9
Cell division	[GO:0051301]	1.02E-6	15	Anatomical structure homeostasis	[GO:0060249]	2.07E-6	9
Publication	PMID	*p*-Value	#	Publication	PMID	*p*-Value	#
Clustering time-varying gene expression profiles using scale-space signals				Genome-wide array-CGH analysis reveals YRF1 gene copy number variation that modulates genetic stability in distillery yeasts			
	[16452778]	9.74E-24	20		[26384347]	2.80E-12	7
Serial regulation of transcriptional regulators in the yeast cell cycle				Transcriptional effects of the potent enediyne anti-cancer agent Calicheamicin gamma(I)(1)			
	[11572776]	6.14E-17	16		[11880039]	1.11E-11	7
Identification of a core set of signature cell cycle genes whose relative order of time to peak expression is conserved across species				Linking DNA replication checkpoint to MBF cell-cycle transcription reveals a distinct class of G1/S genes			
	[22135306]	6.34E-12	10		[22333912]	2.32E-11	11
Identification of sparsely distributed clusters of cis-regulatory elements in sets of co-expressed genes				Mcm1p-induced DNA bending regulates the formation of ternary transcription factor complexes			
	[15155858]	3.71E-10	9		[12509445]	2.35E-11	8
Computational reconstruction of transcriptional regulatory modules of the yeast cell cycle				A genetic screen for yeast genes induced by sustained osmotic stress			
	[17010188]	4.17E-10	12		[12868060]	1.82E-10	7

#: number of genes associated with GO BP terms or mentioned in the publications. PMID: PubMed ID

#### Gene–gene interactions via identification servers, STRING and GeneMania

We also uploaded 37 genes identified by PCA based unsupervised FE and 36 genes extracted from cyclebase to the STRING server, which identified 155 PPIs and 101 PPIs, respectively (*P*=0 for both) while the expected number of PPIs was 30 and 22, respectively. Although both were significant, genes identified by PCA based unsupervised FE identified more PPIs (1.5-fold greater).

**Fig. 6:**
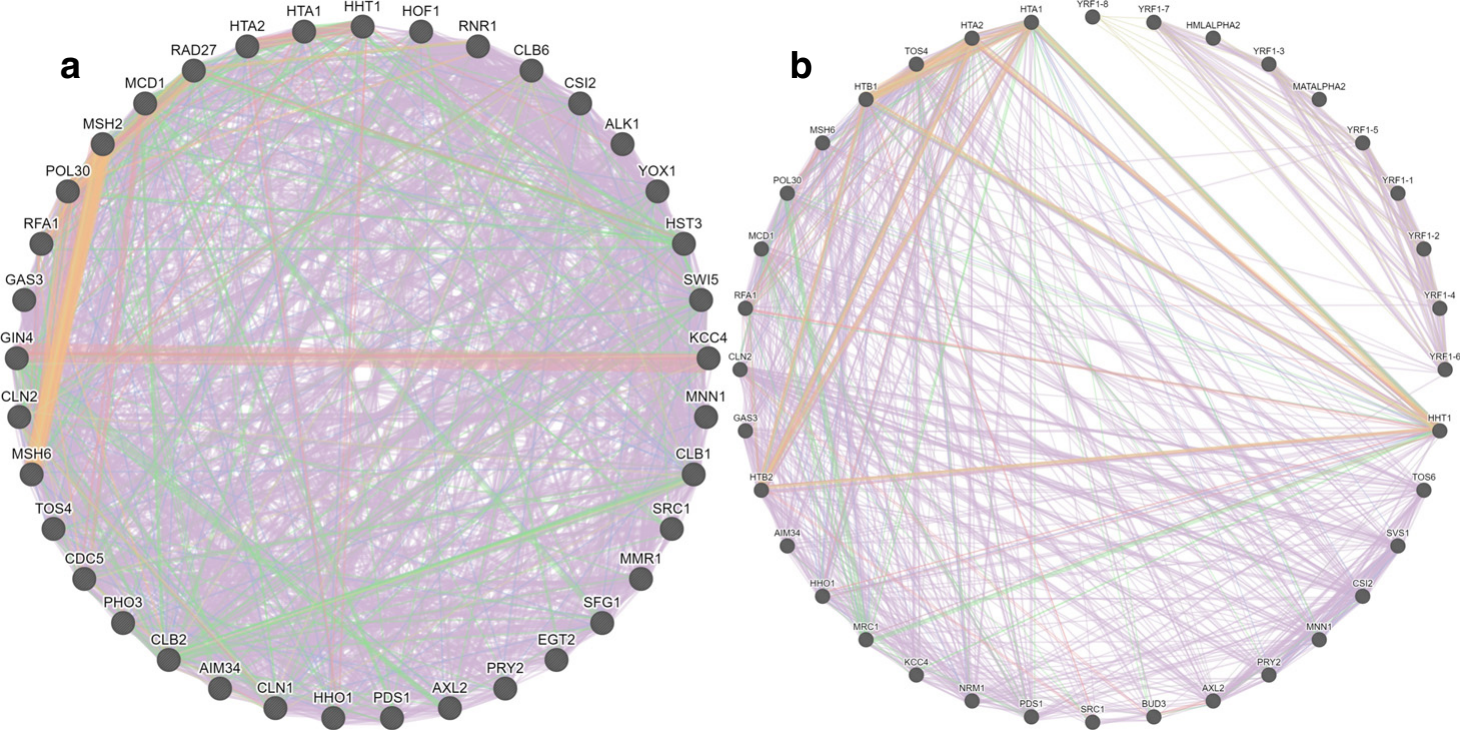
Gene-gene interactions identified by GeneMania [23]. Genes identified by (**a**) PCA based unsupervised FE and (**b**) cyclebase. *Purple*: co-expression, *green*: genetic interaction, *pink*: physical interactions, *orange*: predicted, *blue*: co-localization

Two sets of genes were additionally uploaded to GeneMania [23], another gene–gene interaction identification server. Again, this analysis demonstrated greater numbers of gene–gene interactions between genes identified by PCA based unsupervised FE than those by cyclebase (Fig. 6). Thus, independent of the servers employed, genes identified by PCA based unsupervised FE interacted with each other to a greater degree than those identified by cyclebase.

Taken together, these findings indicate the superiority of PCA based unsupervised FE compared with cyclebase with regards to integrated analysis and enrichment analyses of identified genes. These results demonstrate how PCA based unsupervised FE outperformed sinusoidal fitting.

## Discussion

### Comparison with synthetic data sets

To confirm the superiority of PCA based unsupervised FE over FEs based on sinusoidal fittings, we applied both to synthetic data sets (see Methods). *P* values were attributed to each gene by either PCA based unsupervised FE using the first and second PC score determined by gene embedding or sinusoidal regression. *P*-values were adjusted by the Benjamini and Hochberg (BH) criterion [24] and genes associated with adjusted *P*-values less than 0.01 were selected. Table 2 shows the confusion matrixes averaged over 100 independent ensembles with changing noise-signal ratio *A* from 1 to 6 (gene expression with larger *A* was more disturbed (non-sinusoidal)). PCA based unsupervised FE always achieved 100 % accuracy independent of the amount of *A*, while the accuracy achieved by sinusoidal fitting gradually deceased as *A* increased. This suggested the superiority of PCA based unsupervised FE over sinusoidal regression.

**Table 2:** Confusion matrixes for synthetic data sets

A	1		2		3	
PCA	*P*≥0.01	*P*<0.01	*P*≥0.01	*P*<0.01	*P*≥0.01	*P*<0.01
*i*>100	9900	0	9900	0	9900	0
*i*≤100	0	100	0	100	0	100
Regression	*P*≥0.01	*P*<0.01	*P*≥0.01	*P*<0.01	*P*≥0.01	*P*<0.01
*i*>100	9900	0	9900	17	9900	48
*i*≤100	0	100	0	83	0	52
A	4		5		6	
PCA	*P*≥0.01	*P*<0.01	*P*≥0.01	*P*<0.01	*P*≥0.01	*P*<0.01
*i*>100	9900	0	9900	0	9900	0
*i*≤100	0	100	0	100	0	100
Regression	*P*≥0.01	*P*<0.01	*P*≥0.01	*P*<0.01	*P*≥0.01	*P*<0.01
*i*>100	9900	64	9900	72	9900	85
*i*≤100	0	36	0	28	0	15

*P*-values were adjusted by BH criterion

The reason for this superiority was because PCA based unsupervised FE detected circular structures embedded in a two-dimensional plane spanned by the first and second PC scores attributed to each gene (red open circles in Additional file 9: Figure S2(A)). As shown in Additional file 9: Figure S2(B), circular structures were fully independent of sinusoidal shapes of PC loadings attributed to samples. Despite this, successful regression analysis between pre-defined non-sinusoidal periodic functions (*C*_*j*_ and *S*_*j*_) and PC loadings (*v*_*kj*_,*k*=1,2) suggested the ability of PCA based unsupervised FE to reproduce original non-sinusoidal functional forms (Additional file 9: Figure S2(C) and (D)). Of note, PCA based unsupervised FE correctly identified non-sinusoidal periodic gene expression. This might explain why PCA based unsupervised FE performed well even when gene expression profiles were far from sinusoidal as shown Additional file 5: Figure S1. This supports the robustness of PCA based unsupervised FE and the superiority over sinusoidal regression based FE.

### Usage of g:profiler instead of DAVID

g:profiler was used as an enrichment analysis server instead of the more popular The Database for Annotation, Visualization and Integrated Discovery (DAVID) [25] because our preliminary experiments suggested DAVID identifies less enrichments than g:profiler, which allows DAVID to enhance the superiority of PCA based unsupervised FE more than g:profiler. DAVID overlooked enrichments in 36 genes extracted from cyclebase, while g:profiler did not. In the integrated analysis of YCDC, DAVID did not identify the enrichments of cell division cycle specific GO BP terms in 36 genes extracted from cyclebase including “cell division”, “cell cycle phase”, “M phase”, “mitosis”, “regulation of cell cycle”, and “M phase of mitotic cell cycle”, which were identified by g:profiler. Because the primary purpose of this study was not to demonstrate superiority, but to investigate why PCA based unsupervised FE was superior, too large an outperformance of PCA based unsupervised FE should be avoided. Thus, we decided to use g:profiler instead of DAVID.

### The feasibility of three clusters identified in Fig. 3

**Fig. 7:**
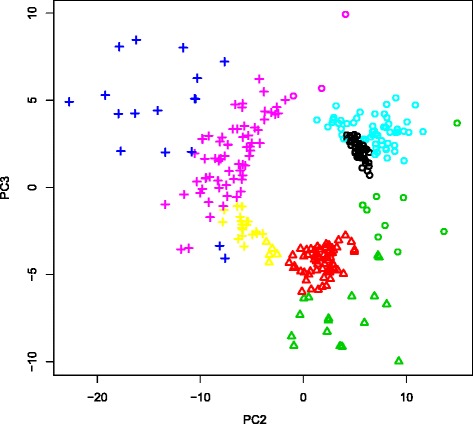
Comparisons between K-means (symbols, identical to those in Fig. 3) and Gaussian mixture (colors, clusters 1 to 7 correspond to *black*, *red*, *green*, *blue*, *cyan*, *pink* and *yellow*, respectively)

The number of clusters in Fig. 3 was assumed to be three based upon a previous study [13]. This estimation was not based upon our own analysis. To determine whether a cluster number of three was justified, we applied a Gaussian mixture clustering algorithm that reports the optimal number of clusters in a data driven way (see Methods). Figure 7 shows the comparison of clustering between K-means and Gaussian mixer. The optimal number of clusters identified by Gaussian mixture was not three, which we employed to perform K-means (Fig. 3). However, the seven clusters identified did not contradict the three clusters identified by K-means. Table 3 shows a comparison between the two identified clusterings. Cluster 1 identified by K-means was mostly composed of clusters 1 and 5 identified by Gaussian mixture; cluster 2 identified by K-means was mostly composed of clusters 2 and 3 identified by Gaussian mixture; and cluster 3 identified by K-means was mostly composed of clusters 4, 6 and 7 identified by Gaussian mixture. Although there were some discrepancies (cluster 3 identified by Gaussian mixtures was divided into clusters 1 and 2 identified by K-means, while a few genes within clusters 6 and 7 identified by Gaussian mixture were classified into K-means-identified clusters 1 and 2 that differed from cluster 3 where the majority of genes in clusters 6 and 7 identified by Gaussian mixture belong), the majority of genes were similarly clustered between K-means and Gaussian mixture.

**Table 3:** Comparison between clusters identified by K-means and Gaussian mixture shown in Fig. 7

	1 (Circle)	2 (Triangle)	3 (Cross)
1 (black)	42	0	0
5 (cyan)	59	0	0
2 (red)	0	61	0
3 (green)	10	17	0
4 (blue)	0	0	17
6 (pink)	3	0	65
7 (yellow)	0	5	19

Rows: Gaussian mixture, columns: K-means

The detailed analysis given by mclust is shown in Additional file 10: Figure S3 also strengthens the feasibility of assuming a cluster number of three. The dependence of Bayesian information criterion (BIC) upon cluster numbers showed a quick increase of BIC up to three clusters while BIC increased slowly between cluster numbers 3 and 7. This suggested that the cluster number of three was of primary importance. In addition, both “Classification Uncertainty” and “log Density Contour Plot” clearly show three clusters, not seven. Taken together, we assumed the three clusters in Fig. 3 were feasible.

### Comparison between PCA based unsupervised FE and FE based on fitting to various periodic functions using biological term enrichments

**Fig. 8:**
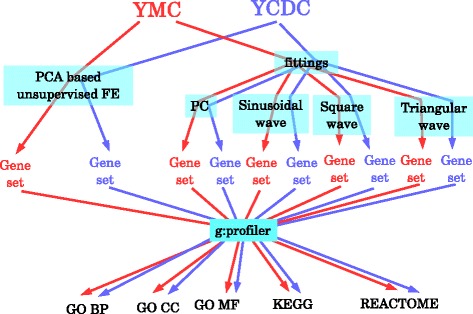
Schematic of comparisons between PCA based unsupervised FE and FE based on fittings to various periodic functions

**Fig. 9:**
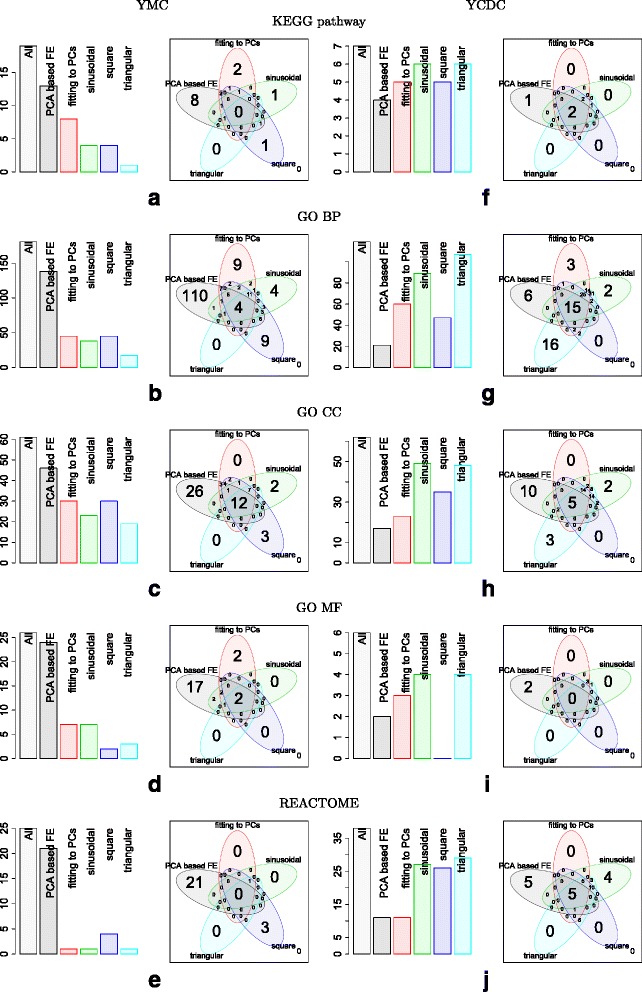
Barplots and Venn diagrams for biological term enrichments. List of biological terms are shown in Additional file 11: Table S7 (YMC; (**a**) to (**e**)) and Additional file 12: Table S8 (YCDC, Cho et al. [20] study of cdc28-13 cells; (**f**) to (**j**)); columns A, B, C, D and E in Additional file 11: Table S7 and Additional file 12: Table S8 correspond to PCA based unsupervised FE, fittings to PCs used for FE (PC1, PC2, PC3, and PC4 for YMC, PC2 and PC3 for YCDC), sinusoidal, square and triangular wave functions, respectively

PCA based unsupervised FE was superior to frequently used sinusoidal fittings from a biological point of view. However, it is still unclear why unsupervised methods can outperform supervised methods. To investigate this, we intentionally performed fittings to YMC and YCDC using other periodic functions, as well as PC loadings used for FEs (see Methods and Fig. 8. A full list of genes identified is shown in Additional file 1: Table S1L to S1S). Gene expression of YCDC was obtained from the cdc28-13 cell experiments reported by Cho et al. [20] that were least biologically significant, because we intended to minimize the superiority of PCA based unsupervised FE as discussed above. The extracted genes were uploaded to g:profiler (see Fig. 9). A list of enriched biological terms in YMC and YCDC are shown in Additional file 11: Table S7 and Additional file 12: Table S8, respectively, whose columns A, B, C, D and E correspond to PCA based unsupervised FE, fittings to PCs used for FE, sinusoidal, square and triangular wave functions, respectively. Bar plots show the number of enriched biological terms are distinct between YMC and YCDC (Fig. 9). The number of enriched biological terms in genes identified by PCA based unsupervised FE were the greatest in YMC, but the smallest in YCDC; the latter indicated that too great a superiority of PCA based unsupervised FE was successfully suppressed as intended. However, Venn diagrams did not show much distinction between YMC and YCDC but exhibited a distinction between PCA based unsupervised FE and FE based upon fittings; genes identified by PCA based unsupervised FE were always accompanied by biological terms not enriched in fittings that was not dependent on how many enrichments were identified in genes identified by PCA based unsupervised FE. For example, three of five REACTOME pathways enriched in YCDC only identified by PCA based unsupervised FE were “Mitotic G2-G2/M phases”, “G2/M Transition”, and “Cyclin A/B1 associated event during G2/M transition”, which are highly cell division cycle specific. Ten GO CC terms enriched in YCDC only identified by PCA based unsupervised FE included “cell periphery”, “cell wall” and “fungal type cell wall”, which are also highly cell division cycle specific. Thus, PCA based unsupervised FE can identify biological terms not detected by FE based upon fittings, despite the selection of gene expression profiles to which PCA based unsupervised FE achieved the least performance.

This might explain why PCA based unsupervised FE can outperform FE based upon fittings to functions including sinusoidal functions, because PCA based unsupervised FE must be distinct from FE based upon fittings to outperform them. Of note, PCA based unsupervised FE and FE based upon fittings to PCs were comparable because both employed the same functions, with the only difference being how to rank gene expression profiles based upon PCs. FE based upon fittings evaluates gene expression profiles using correlations while PCA based unsupervised FE evaluates gene expression profiles using the amount of projection to the plane spanned by PCs. FE based on fittings to PCs has an inferior ability to extract genes not extracted by sinusoidal, square or triangular wave functions compared with PCA based unsupervised FE, because the numbers filled in the region that exhibits genes extracted by FE only based upon fittings to PCs is as small as FE based upon fittings to either sinusoidal, square or triangular wave functions (Fig. 9). This can be understood as follows.

Suppose that *x*(*t*) is a gene expression time course of a gene at time *t* and *x*(*t*) is composed of two parts; i) a part not coincident with the considered functional form (thus, apparently assumed to be biologically irrelevant, noisy) part *x*_*n*_(*t*) and ii) a significant part *x*_*s*_(*t*) that is coincident with the considered functional forms, e.g., PCs or various periodic functions (Fig. 11), i.e., *x*(*t*)=*x*_*n*_(*t*)+*x*_*s*_(*t*). Fitting to PCs evaluates each gene by the ratio of *x*_*s*_(*t*) to *x*(*t*) because it makes use of the correlation between *x*(*t*) and *x*_*s*_(*t*), while PCA based unsupervised FE evaluates the amount of *x*_*s*_(*t*) because it measures the projection onto the plane spanned by two PCs used for FE. This is the main difference between the two methodologies. Because a projection-based approach, i.e., PCA based unsupervised FE, seems to outperform other FEs based upon fittings, rankings based upon projections are more biologically feasible in the present study than those based upon correlations.

This may be reasoned biologically as follows: genes often have multi-functional effects, thus *x*_*n*_(*t*) should not be regarded as a penalty, but should be simply ignored, because *x*_*n*_(*t*) may not be noise but is considered not to have a function, e.g., housekeeping genes. Alternatively the superiority of projection to correlation may also be interpreted biologically as follows: under biological situations where periodic motions are strongly induced, many genes passively exhibit periodic gene expression. This phenomenon can be observed in other organisms; for example, although many genes in cyanobacteria exhibit circadian rhythms, they are suppressed by the knockout of a small number of genes [26]. Therefore, sinusoidal fittings are not always a good strategy to identify genes that induce circadian rhythms, because passively oscillating genes may also exhibit circadian rhythm. Similarly, in YMC and YCDC, simple fittings to periodic functions or PCs are inferior to PCA based unsupervised FE, which consider projections onto *x*_*s*_(*t*) that exhibit biologically feasible periodic motion rather than a correlation between *x*(*t*) and *x*_*s*_(*t*).

**Fig. 10:**
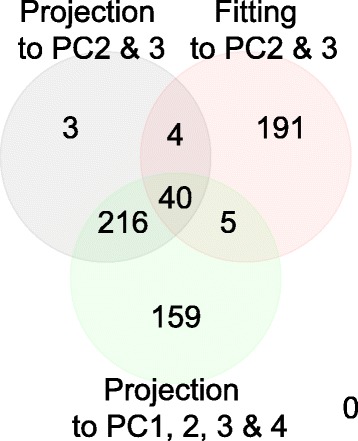
Venn diagram between genes identified by PCA based unsupervised with PC2 and PC3 (*gray*), with PC1, PC2, PC3 and PC4 (*green*) and by FE based upon fittings to PC2 and PC3 (*red*), in YMC [13]

This is illustrated in Fig. 10, which shows a Venn diagram in YMC between genes identified by PCA based unsupervised FE using only PC2 and PC3, genes selected based on fittings to PC2 and PC3, and genes identified by PCA based unsupervised FE using PC1, PC2, PC3 and PC4. As discussed above, the genes in YMC identified by PCA based unsupervised FE using PC1, PC2, PC3, and PC4 were biologically feasible. Genes identified by PCA based unsupervised FE using only PC2 and PC3 almost overlapped with the subset of genes identified by PCA based unsupervised FE using PC1, PC2, PC3 and PC4, while genes selected by FE based on fittings to PC2 and PC3 do not. This suggests that projection is better than correlation if *x*_*n*_(*t*) cannot be definitely considered to be noise, and thus biologically irrelevant.

### Comparisons with other unsupervised clustering and embedding methods

#### Previous unsupervised studies of YMC/YCDC

Finally, we investigated the comparison between PCA based unsupervised FE and other apparently unsupervised FE when applied to YCDC or YMC. For example, Tamayo et al. [27] applied SOM to YCDC and reported the identification of cell cycle regulated genes in an unsupervised manner. However, they had to filter genes before applying SOM and there was no discussion regarding how they determined the filtering criteria. In addition, SOM has many parameters that must be tuned, e.g., number of cells and lattice type on which cells are located. It is not clear how many were performed before obtaining their best results, and thus this system might be considered not to be unsupervised. To decide which cluster should be used for FE, they selected clusters associated with known cell division cycles. This is a potential limitation because gene expression profiles associated with a period distinct from the cell cycle period might be missed. Another example is a study by Rowicka et al. [28], who identified cell cycle regulated genes without specifying cell division cycle using an entropy method; however, they extracted genes associated with gene expression similar to known cell cycle regulated genes. Thus, to our knowledge, no methods as unsupervised as ours have successfully identified cell cycle regulated genes. The only assumption we made was that gene expression must be periodic (i.e., winding number analysis) regardless of the period.

#### Other unsupervised clustering

To emphasize further the superiority of PCA based unsupervised FE over ordinary (unsupervised) clustering, we investigated other (unsupervised) clustering methods. We applied four frequently used clusterings to YMC (see Additional file 13: Document S2). We found no clustering methods that could compete with PCA based unsupervised FE because clustering methods have no ability to separate aperiodic (thus seemingly noisy) profiles from periodic profiles, which is successfully achieved by PCA based unsupervised FE (Fig. 3a). Thus, the four (unsupervised) clustering methods tested could not form clusters whose representative profiles were as periodic as those shown in Fig. 3b. Therefore, we concluded that PCA based unsupervised FE was superior to the four (unsupervised) clustering methods.

#### Other unsupervised embedding methodologies

We also investigated replacing PCA with other embedding methods. The usage of kernel tricks [29] together with an embedding method was unsuccessful because it provided nothing to correspond with PC loadings, which enables the biological interpretation of embeddings and specification of which PCs should be used for FE. In contrast, independent component analysis (ICA) [30] could replace PCA, because it provides mixing weights that correspond to PC loadings. However, after replacing PCA with ICA for YMC analyses, we found that ICA often provided more than two almost identical profiles as independent components, because ICA attempts to maximize the overall (average) mutual independences among components; thus, local independence (independence among a specific pair of components) is not guaranteed. Furthermore, ICA does not provide variables that can be used to evaluate the importance of a PC in PCA. Therefore, identifying components used for FE in ICA is more difficult. Currently we do not have anything to replace PCA in PCA based unsupervised FE.

## Conclusions

In this study, we applied the recently proposed PCA based unsupervised FE to two budding yeast cell division time course data sets. It outperformed conventional supervised sinusoidal fitting methodologies, which demonstrated the superiority of the unsupervised method over the supervised method. This might explain why PCA based unsupervised FE often outperformed supervised methods when previously applied to various problems. A comparison study between PCA based unsupervised FE and other fitting based FEs identified the mechanism involved in why PCA based unsupervised FE outperforms sinusoidal fitting based FEs.

## Methods

### Relationships between the figures and tables

Because we have presented many figures and tables whose relationships are very complicated, these are explained in Additional file 14: Figure S4. Please note that Additional file 2: Table S2 and Additional file 6: S4A are equivalent to Additional file 11: Table S7A and Additional file 12: S8A, to enhance their understanding.

### Gene expression profiles

The YMC gene expression profiles analyzed were downloaded from the Gene Expression Omnibus (GEO) with GEO ID GSE3431. A file “GSE3431_series_matrix.txt” included in “Series Matrix File(s)” was downloaded. YCDC gene expression was downloaded from cyclebase [16]. They were normalized to have a mean of 0 and a variance of 1 within each sample (i.e., ${\frac {1}{N} \sum _{i} x_{ij}=0}$ and ${\frac {1}{N}\sum _{i} x_{ij}^{2}=1}$, where *N* is the total number of genes. No further normalization procedures were applied).

### PCA based unsupervised FE

Although our proposed method, PCA based unsupervised FE, was extensively and successfully applied to various biological problems [1*–*12*,*^31^*–*33], we briefly review the methodology here. The method is composed of two parts: i) gene embedding and ii) gene selection (Fig. 2).

Briefly, PCA based unsupervised FE, in contrast to the ordinary usage of PCA, uses features (genes) embedded into the low dimensional space rather than samples. After specifying PCs that exhibit biological significance, features as outliers along the specified PC are extracted as important features. The philosophy behind this methodology is that if a set of features have common dependence upon samples, no matter what they are, they are more likely to construct PCs because PCs represent the majority of behaviors. Samples dependent on PCs likely represent biological significance, e.g., the distinction between control and treated samples. Although there is no evidence to support this hypothesis, it is a simple methodology that is not computationally challenging. Gene expression profiles are normalized to have a mean of zero and unit variance before applying PCA.

#### Gene embedding by PCA

Suppose that we have mRNA expression *x*_*ij*_ of *i*th mRNA of *j*th sample. It is also supposed that ${\frac {1}{N}\sum _{i=1}^{N} x_{ij}=0}$ and ${\frac {1}{N} \sum _{i=1}^{N} x_{ij}^{2}} =1$. *X* is the matrix whose element is *x*_*ij*_. In contrast to the usual usage of PCA, where samples are embedded, genes (mRNAs) are embedded in the PCA based upon unsupervised FE. Then *k*th PC score *u*_*ki*_ attributed to *i*th gene can be computed as the element of eigenvector **u**_*k*_ of the Gram matrix *G*≡*XX*^*T*^, *XX*^*T*^***u***_*k*_=*λ*_*k*_***u***_*k*_ where *λ*_*k*_ is eigen value ordered such that *λ*_*k*+1_<*λ*_*k*_. The *k*th PC loading *v*_*kj*_ attributed to *j*th sample can be computed as the element of **v**_*k*_=*X*^*T*^**u**_*k*_, which is the eigenvector of the matrix *X*^*T*^*X*, because *X*^*T*^*X****v***_*k*_=*X*^*T*^*XX*^*T*^***u***_*k*_=*X*^*T*^*λ*_*k*_***u***_*k*_=*λ*_*k*_***v***_*k*_.

#### Winding number analysis

To identify limit cycles represented by the series of vectors composed of pairs of PC loadings, we used $\boldsymbol {v}^{k,k'}_{j} \equiv \left (\begin {array}{c} {v_{kj}} - \langle {v_{kj}} \rangle _{j} \\ v_{k'j} - \langle v_{k'j} \rangle _{j} \end {array}\right), j=1,\cdots,M$, where *M* is the number of time points and $\langle {v^{k}_{j}} \rangle _{j} = \frac {1}{M} \sum _{j} {v_{kj}}$ is used to introduce winding number analysis. Winding number represents the number of times “orbits” rotate around the origin. Winding number, *W*, is defined as $W(M') \equiv \sum _{j=1}^{M'} \frac {\Delta \theta _{j,j+1}}{2\pi }, M'<M$, where *Δθ*_*j,j*+1_ represents the incremental (signed) angle between subsequent vectors $\boldsymbol {v}^{k,k'}_{j}$ and $\boldsymbol {v}^{k,k'}_{j+1}$, which is specifically defined as $\Delta \theta _{j,j+1} \equiv \frac {\boldsymbol {v}^{k,k'}_{j} \cdot {\boldsymbol {v}^{k,k'}_{j+1}}} {\mid {\boldsymbol {v}^{k,k'}_{j}} \mid \mid {\boldsymbol {v}^{k,k'}_{j+1}} \mid }\text {sign} \left ({\boldsymbol {v}^{k,k'}_{j}} \times {\boldsymbol {v}^{k,k'}_{j+1}} \right) $ where sign(*x*) takes ±1 dependent upon the sign of *x*. We extracted outliers along the pair of *k*th and *k*^′^th PC loadings associated with the largest ∣*W*(*M*−1)∣ within the range *k,k*^′^≤4, because orbits do not seem to be limit cycles for some pair *k,k*^′^>4 with sufficiently large ∣*W*(*M*−1)∣. In addition, for the gene expression shown in Additional file 5: Figure S1D, time points *j*>30 were excluded when computing PC scores used for FE, because the time points *j*>30 seemingly deviated from limit cycles and using points *j*>30 substantially decreased the number of genes extracted as outliers.

#### Feature extraction

Gene embedding was performed in PCA based unsupervised FE. Then after identifying a set *Ω*_*k*_ of PCs whose PC loading were coincident with the distinction between treated and control samples, outlier genes were identified by assuming a Gaussian distribution of PC scores using *χ* square distribution, $ P_{i} = P \left [ \sum _{k \in \Omega _{k}} \left (\frac {u_{ki}}{\sigma _{k}} \right)^{2} > x \right ] $, where *P*[>*x*] is the cumulative probability of *χ* squared distribution when the argument is larger than *x* and *σ*_*k*_ is the standard deviation of *k*th PC scores. Then, if BH criterion [24] adjusted *P*_*i*_<0.01 (for YMC) or *P*_*i*_<0.05 (for YCDC), the *i*th gene is identified as an outlier.

### Enrichment analysis using g:profiler and YeastMine

Extracted gene IDs in YMC and YCDC were converted to gene symbols based on the probe annotation file available at GEO ID: GPL90 (although GPL90 was associated with GEO ID GSE3431, we used it for cyclebase and unified analysis). A list of gene symbols were uploaded to the “Cocoa:Compact Compare of Annotations” pages included in the g:profiler web pages. Output was extracted as either an Excel or PDF file by specifying the output type option. Gene symbols were also uploaded to YeastMine. *P*-values were adjusted by specifying the BH criterion.

### K-means clustering and Gaussian mixture

PC2 and PC3 scores of extracted genes were processed by the kmeans function included in R [34]. To compensate for the initial configuration dependence of K-means, majority clustering was identified within 100 trials by specifying nstart=100. Gaussian mixture clustering was performed for the same gene set using the Mclust function in the mclust package [35] and R [34] with default settings.

### PPI identification via STRING and gene–gene interaction identification via GeneMania

For both servers, gene symbols used for enrichment analyses were uploaded. For STRING, after selecting the “multiple proteins” menu, organism was specified in the pull down menu below (“*Saccharomyces cerevisiae*”). “PPI enrichment *p*-value” will appear under the “Analysis” tab. For GeneMania, in “Customise Advanced options” menu, both “Max resultant genes/attributes” were set to zero to identify only interactions within the uploaded genes.

### Regression analysis for FE based on fitting

**Fig. 11:**
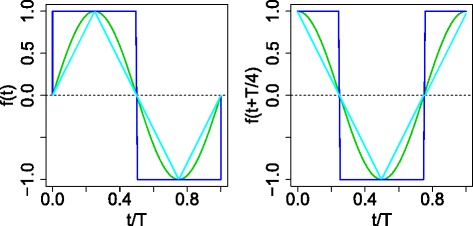
Periodic fitting functions *f*(*t*) (*left*) and $f(t+\frac {T}{4})$: sinusoidal (*green*), square (*blue*) and triangular (*cyan*) wave functions; colors correspond to those used in Fig. 9

Gene expression profiles were fitted toward the following regression function: $ x(t) = C_{0} + C_{1} f(t) + C_{2} f \left (t+\frac {T}{4}\right) $ where *f*(*t*) is a periodic function that satisfies *f*(*t*+*T*)=*f*(*t*). For simplicity, we considered only symmetric functions satisfying the following two conditions: $ f \left (t- \frac {T}{4} \right) = f \left (\frac {T}{4} - t\right) $ and $ f \left (t + \frac {T}{2} \right) = - f (t)$. This requires that the function should be symmetric with the first half of one period and that amplitudes are reversed between the first half and the latter half within one period. Then, the functions shown in Fig. 11 (sinusoidal, step and triangular wave) were specifically employed to determine the difference between distinct periodic functions sharing the same period, *T*; *T* corresponded to 12 and 8 times points for YMC [13] and YCDC [20], respectively. Therefore, 36 time points in YMC and 16 time points in YCDC were regarded as three times and two times as long as the cell division cycle period, respectively. The numbers of gene IDs extracted based upon regression analyses were equivalent to the number of gene IDs extracted by PCA based unsupervised FE, because the purpose of regression analysis was to compare the significance of extracted genes between PCA based unsupervised FE and those identified by regression analysis. If we uploaded a distinct number of genes to g:profiler between PCA based unsupervised FE and FE based upon fitting, FE with larger (smaller) genes would achieve more (less) significance. This may prevent a comparison of performances between PCA based unsupervised FE and FE based upon fittings.

### Synthetic data set

Suppose *x*_*ij*_ is the gene expression of the *i*th gene at the *j*th sample. *i*=1,…,10^4^,*j*=1,…,10^2^.

$$\begin{array}{@{}rcl@{}} {S^{0}_{j}} & = & \sin \left \{\frac{2 \pi}{25} (j ~\text{mod}~ 25)\right\} \\ {C^{0}_{j}} & = & \cos \left \{\frac{2 \pi}{25} (j ~\text{mod}~ 25)\right\} \\ S_{j} & = & {S^{0}_{j}} + \epsilon^{S}_{j ~\text{mod}~ 25} \\ {C^{1}_{j}} & = & {C^{0}_{j}} + \epsilon^{C}_{j ~\text{mod}~ 25} \\ C_{j} & = & {C^{1}_{j}} - S_{j} \frac{\sum_{j} {C^{1}_{j}} S_{j}}{\sum_{j} {S_{j}^{2}}} \\ x^{0}_{ij} & = & \left \{\begin{array}{cc} C_{j} \cos \delta_{i} + S_{j} \sin \delta_{i},& i \leq 10^{2} \\ \epsilon_{ij}, & i > 10^{2} \end{array} \right. \\ x_{ij}&= & \frac{x^{0}_{ij}}{\sqrt{\frac{1}{100}\sum_{j} \left(x^{0}_{ij} - \frac{1}{100} \sum_{j} x^{0}_{ij} \right)^{2}}} \end{array} $$

where $\epsilon ^{S}_{j ~\text {mod}~ 25}, \epsilon ^{C}_{j ~\text {mod}~ 25}, \delta _{i}, \epsilon _{ij}$ are uniform random numbers in the range of [−*A,A*],[−*A,A*],[0,2*π*], and [−1,1], respectively. These correspond to the linear combinations of noise added/orthogonalized sinusoidal functions ranging over four periods. *A* represents the ratio of noise-to-signal (pure sinusoidal function); a larger *A* causes *C*_*j*_ and *S*_*j*_ to become more distant from pure sinusoidal functions. Please note that *C*_*j*_ and *S*_*j*_ for *j*=1,…10^2^ remain as complete periodic functions despite the addition of noises, $\epsilon ^{S}_{j ~\text {mod}~ 25}$ and $\epsilon ^{C}_{j ~\text {mod}~ 25}$, because noises are also periodic functions.

*P*-values were attributed to *x*_*ij*_ assuming the sinusoidal regression equation

$$x_{ij} = \alpha_{i} {C^{0}_{j}} + \beta_{i} {S^{0}_{j}} + \gamma_{i} $$

where *α*_*i*_, *β*_*i*_ and *γ*_*i*_ are regression coefficients. The lm function in R [34] was used for regression analysis.

## Additional files

Additional file 1
**Table S1.** The list of genes selected by PCA based unsupervised FE for various experiments (data sets). See sheet 1 for more details regarding which sheet corresponds to which experiments. (XLSX 31 kb)

Additional file 2
**Table S2.** Enrichment analyses by g:profiler for the genes listed in Table S1A. (PDF 992 kb)

Additional file 3 Document S1. Genes shown in Fig. 3. Black circles, red triangles, and green crosses are annotated as Cluster_1, Cluster_2, and Cluster_3, respectively. (PDF 19.2 kb)

Additional file 4
**Table S3.** Enrichment analyses by g:profiler for the genes listed in document S1. (PDF 1361 kb)

Additional file 5
**Figure S1.** Winding numbers and scatter plots of seven YCDC data sets. (PDF 61.7 kb)

Additional file 6
**Table S4.** Enrichment analyses by g:profiler for the genes listed in Table S1C to S1I, each of which corresponds to A to G, respectively. (PDF 235 kb)

Additional file 7
**Table S5.** Enrichment analyses by g:profiler for the genes listed in Table S1J (PCA) and S1K (CYCLEBASE). (PDF 433 kb)

Additional file 8
**Table S6.** Enrichment analysis by YeastMine, the rest of Table 1. (XLSX 114 kb)

Additional file 9
**Figure S2.** PCA based unsupervised FE results for synthetic data (*A*=1,2,3,4,5,6). Representative examples (one of 100 ensembles, from which the averages shown in Table 2 were taken). (A) The first and second PCA scores attributed to genes. Red open circles correspond to genes associated with significant adjusted *P*-values (<0.01), which are equivalent to genes *i*≤10^2^. (B) The first and second PC loading attributed to each sample. Black: 1≤*j*≤25, red: 26≤*j*≤50, green: 51≤*j*≤75, blue: 76≤*j*≤100. (C) Regression analysis, *C*_*j*_=*av*_1*j*_+*bv*_2*j*_. Black: *C*_*j*_, red: fitted results. Blue: ${C^{0}_{j}}$. (D) Regression analysis, *S*_*j*_=*av*_1*j*_+*bv*_2*j*_. Black: *S*_*j*_, red: fitted results. Blue: ${S^{0}_{j}}$. (PDF 402 kb)

Additional file 10
**Figure S3.** Detailed results obtained from mclust (Gaussian mixture). (PDF 30.5 kb)

Additional file 11
**Table S7.** Enrichment analyses by g:profiler for the YMC genes that correspond to Figure 9A to 9E. (PDF 229 kb)

Additional file 12
**Table S8.** Enrichment analyses by g:profiler for YCDC, cdc28-13 cells from the study by Cho *et al* [20], genes that correspond to Figure 9F to 9J. (PDF 1259 kb)

Additional file 13 Document S2. Performances of four other unsupervised clustering methods. (PDF 252 kb)

Additional file 14
**Figure S4.** Relationship between figures and tables. (PDF 114 kb)

Additional file 15 Reviewer reports and AU response to reviewers. (DOCX 35.8 kb)

## Abbreviations

BH: Benjamini and Hochberg
BIC: Bayesian information criterion
BP: biological process
CC: cellular component
DAVID: the database for annotation, visualization and integrated discovery
FE: feature extraction
GEO: gene expression omnibus
GO: gene ontology
ICA: independent component analysis
KEGG: Kyoto encyclopedia of genes and genomes
MF: molecular function
PC: principal component
PCA: principal component
PI: protein-protein interactions
YCDC: yeast cell division cycle
YMC: yeast metabolic cycle

## References

[1] Taguchi YH, Iwadate M, Umeyama H (2015). Principal component analysis-based unsupervised feature extraction applied to in silico drug discovery for posttraumatic stress disorder-mediated heart disease. BMC Bioinforma.

[2] Taguchi Y-h, Iwadate M, Umeyama H, Murakami Y, Okamoto A, Wang B, Li R, Perrizo W (2015). Heuristic principal component analysis-aased unsupervised feature extraction and its application to bioinformatics. Big Data Analytics in Bioinformatics and Healthcare.

[3] Taguchi Y-h, Okamoto A, Shibuya T, Kashima H, Sese J, Ahmad S (2012). Principal component analysis for bacterial proteomic analysis. Pattern Recognition in Bioinformatics. LNCS, vol. 7632.

[4] Murakami Y, Toyoda H, Tanahashi T, Tanaka J, Kumada T, Yoshioka Y, Kosaka N, Ochiya T, Taguchi YH (2012). Comprehensive miRNA expression analysis in peripheral blood can diagnose liver disease. PLoS ONE.

[5] Ishida S, Umeyama H, Iwadate M, Taguchi YH (2014). Bioinformatic screening of autoimmune disease genes and protein structure prediction with FAMS for drug discovery. Protein Pept Lett.

[6] Taguchi YH, Murakami Y (2013). Principal component analysis based feature extraction approach to identify circulating microRNA biomarkers. PLoS ONE.

[7] Kinoshita R, Iwadate M, Umeyama H, Taguchi YH (2014). Genes associated with genotype-specific DNA methylation in squamous cell carcinoma as candidate drug targets. BMC Syst Biol.

[8] Taguchi YH, Murakami Y (2014). Universal disease biomarker: can a fixed set of blood microRNAs diagnose multiple diseases?. BMC Res Notes.

[9] Murakami Y, Tanahashi T, Okada R, Toyoda H, Kumada T, Enomoto M, Tamori A, Kawada N, Taguchi YH, Azuma T (2014). Comparison of Hepatocellular Carcinoma miRNA Expression Profiling as Evaluated by Next Generation Sequencing and Microarray. PLoS ONE.

[10] Umeyama H, Iwadate M, Taguchi YH (2014). TINAGL1 and B3GALNT1 are potential therapy target genes to suppress metastasis in non-small cell lung cancer. BMC Genomics.

[11] Taguchi Y-h (2014). Integrative analysis of gene expression and promoter methylation during reprogramming of a non-small-cell lung cancer cell line using principal component analysis-based unsupervised feature extraction. Intelligent Computing in Bioinformatics. LNCS, vol. 8590.

[12] Taguchi YH (2016). Identification of more feasible microRNA-mRNA interactions within multiple cancers using principal component analysis based unsupervised feature extraction. Int J Mol Sci..

[13] Tu BP, Kudlicki A, Rowicka M, McKnight SL (2005). Logic of the yeast metabolic cycle: temporal compartmentalization of cellular processes. Science.

[14] Reimand J, Arak T, Vilo J (2011). g:Profiler–a web server for functional interpretation of gene lists (2011 update). Nucleic Acids Res.

[15] Szklarczyk D, Franceschini A, Wyder S (2015). STRING v10: protein-protein interaction networks, integrated over the tree of life. Nucleic Acids Res.

[16] Santos A, Wernersson R, Jensen LJ (2015). Cyclebase 3.0: a multi-organism database on cell-cycle regulation and phenotypes. Nucleic Acids Res.

[17] de Lichtenberg U, Wernersson R, Jensen TS, Nielsen HB, Fausbøll A, Schmidt P, Hansen FB, Knudsen S, Brunak S (2005). New weakly expressed cell cycle-regulated genes in yeast. Yeast.

[18] Pic A, Lim FL, Ross SJ, Veal EA, Johnson AL, Sultan MR, West AG, Johnston LH, Sharrocks AD, Morgan BA (2000). The forkhead protein Fkh2 is a component of the yeast cell cycle transcription factor SFF. EMBO J.

[19] Bulmer R, Pic-Taylor A, Whitehall SK, Martin KA, Millar JB, Quinn J, Morgan BA (2004). The forkhead transcription factor Fkh2 regulates the cell division cycle of Schizosaccharomyces pombe. Eukaryot Cell.

[20] Cho RJ, Campbell MJ, Winzeler EA, Steinmetz L, Conway A, Wodicka L, Wolfsberg TG, Gabrielian AE, Landsman D, Lockhart DJ, Davis RW (1998). A genome-wide transcriptional analysis of the mitotic cell cycle. Mol Cell.

[21] Gauthier NP, Larsen ME, Wernersson R, de Lichtenberg U, Jensen LJ, Brunak S, Jensen TS (2008). Cyclebase.org–a comprehensive multi-organism online database of cell-cycle experiments. Nucleic Acids Res.

[22] Balakrishnan R, Park J, Karra K, Hitz BC, Binkley G, Hong EL, Sullivan J, Micklem G, Cherry JM (2012). YeastMine–an integrated data warehouse for Saccharomyces cerevisiae data as a multipurpose tool-kit. Database (Oxford).

[23] Zuberi K, Franz M, Rodriguez H, Montojo J, Lopes CT, Bader GD, Morris Q (2013). GeneMANIA prediction server 2013 update. Nucleic Acids Res.

[24] Benjamini Y, Hochberg Y (1995). Controlling the false discovery rate: A practical and powerful approach to multiple testing. J R Stat Soc Ser B Methodol.

[25] Huang daW, Sherman BT, Lempicki RA (2009). Systematic and integrative analysis of large gene lists using DAVID bioinformatics resources. Nat Protoc.

[26] Nakahira Y, Katayama M, Miyashita H, Kutsuna S, Iwasaki H, Oyama T, Kondo T (2004). Global gene repression by KaiC as a master process of prokaryotic circadian system. Proc Natl Acad Sci USA.

[27] Tamayo P, Slonim D, Mesirov J, Zhu Q, Kitareewan S, Dmitrovsky E, Lander ES, Golub TR (1999). Interpreting patterns of gene expression with self-organizing maps: methods and application to hematopoietic differentiation. Proc Natl Acad Sci USA.

[28] Rowicka M, Kudlicki A, Tu BP, Otwinowski Z (2007). High-resolution timing of cell cycle-regulated gene expression. Proc Natl Acad Sci USA.

[29] Schiilkopf B (2001). The kernel trick for distances. Advances in Neural Information Processing Systems 13: Proceedings of the 2000 Conference.

[30] Hyvärinen A (1999). Fast and robust fixed-point algorithms for independent component analysis. IEEE Trans Neural Netw.

[31] Taguchi YH (2015). Identification of aberrant gene expression associated with aberrant promoter methylation in primordial germ cells between E13 and E16 rat F3 generation vinclozolin lineage. BMC Bioinforma.

[32] Taguchi YH, Iwadate M, Umeyama H. Heuristic principal component analysis-based unsupervised feature extraction and its application to gene expression analysis of amyotrophic lateral sclerosis data sets. In: Computational Intelligence in Bioinformatics and Computational Biology (CIBCB), 2015 IEEE Conference On: 2015. p. 1–10, doi:10.1109/CIBCB.2015.7300274.

[33] Murakami Y, Kubo S, Tamori A, Itami S, Kawamura E, Iwaisako K, Ikeda K, Kawada N, Ochiya T, Taguchi YH (2015). Comprehensive analysis of transcriptome and metabolome analysis in Intrahepatic Cholangiocarcinoma and Hepatocellular Carcinoma. Sci Rep.

[34] R Core Team (2014). R: A Language and Environment for Statistical Computing.

[35] Chris Fraley AER (2002). Model-based clustering, discriminant analysis, and density estimation. J Am Stat Assoc.

