# Supplementary material for: Principal component analysis based unsupervised feature extraction applied to budding yeast temporally periodic gene expression
Source: BioData Min. 2016 Jun 29;9:22. doi: 10.1186/s13040-016-0101-9 (PMC4928327; doi:10.1186/s13040-016-0101-9)
Supplement: Additional file 2 — Table S2. Enrichment analyses by g:profiler for the genes listed in Table S1A. (PDF 992 kb) [file 13040_2016_101_MOESM2_ESM.pdf]

source	term name<br>Gene Ontology (Biological process)	term ID	n. of term genes	corrected p-value	
BP	protein folding	GO:0006457	110	9.41e-04	22
BP	'de novo' protein folding	GO:0006458	11	1.05e-03	7
BP	protein refolding	GO:0042026	17	2.33e-04	9
BP	ribosomal small subunit assembly	GO:0000028	16	2.78e-02	7
BP	monosaccharide metabolic process	GO:0005996	91	1.12e-02	18
BP	hexose metabolic process	GO:0019318	85	4.05e-03	18
BP	glucose metabolic process	GO:0006006	68	1.15e-04	18
BP	monosaccharide biosynthetic process	GO:0046364	35	5.95e-03	11
BP	hexose biosynthetic process	GO:0019319	34	4.32e-03	11
BP	gluconeogenesis	GO:0006094	33	3.09e-03	11
BP	translation	GO:0006412	476	1.31e-16	84
BP	cytoplasmic translation	GO:0002181	171	1.96e-42	74
BP	translational elongation	GO:0006414	100	1.17e-02	19
BP	rRNA transport	GO:0051029	18	1.20e-06	11
BP	rRNA export from nucleus	GO:0006407	18	1.20e-06	11
BP	metabolic process	GO:0008152	4109	3.57e-06	307
BP	organic substance metabolic process	GO:0071704	3723	1.44e-07	290
BP	carbohydrate derivative metabolic process	GO:1901135	367	3.20e-12	65
BP	organonitrogen compound metabolic process	GO:1901564	651	1.67e-17	103
BP	pyridine-containing compound metabolic process	GO:0072524	100	1.50e-10	30
BP	purine-containing compound metabolic process	GO:0072521	170	6.49e-24	56
BP	primary metabolic process	GO:0044238	3554	1.25e-05	274
BP	biosynthetic process	GO:0009058	2100	6.68e-03	172
BP	organic substance biosynthetic process	GO:1901576	2070	4.07e-03	171
BP	organonitrogen compound biosynthetic process	GO:1901566	385	4.21e-04	50
BP	single-organism metabolic process	GO:0044710	2051	1.25e-05	180
BP	small molecule metabolic process	GO:0044281	789	3.94e-29	136
BP	small molecule biosynthetic process	GO:0044283	315	2.80e-02	39
BP	nucleobase-containing small molecule metabolic process	GO:0055086	279	1.66e-22	70
BP	single-organism catabolic process	GO:0044712	398	1.17e-03	50
BP	small molecule catabolic process	GO:0044282	113	2.07e-02	20
BP	glycosyl compound metabolic process	GO:1901657	165	1.25e-25	57
BP	glycosyl compound biosynthetic process	GO:1901659	65	3.16e-04	17
BP	nucleoside metabolic process	GO:0009116	161	2.80e-26	57
BP	ribonucleoside metabolic process	GO:0009119	154	1.80e-26	56
BP	purine nucleoside metabolic process	GO:0042278	140	6.08e-27	54
BP	purine ribonucleoside metabolic process	GO:0046128	140	6.08e-27	54
BP	nucleoside biosynthetic process	GO:0009163	61	1.15e-04	17
BP	ribonucleoside biosynthetic process	GO:0042455	60	8.77e-05	17
BP	oxidation-reduction process	GO:0055114	437	3.77e-19	84
BP	cellular process	GO:0009987	4884	8.37e-10	354
BP	cellular metabolic process	GO:0044237	3696	1.78e-09	294
BP	generation of precursor metabolites and energy	GO:0006091	186	1.89e-23	58
BP	energy derivation by oxidation of organic compounds	GO:0015980	152	6.26e-15	43
BP	cellular respiration	GO:0045333	103	1.69e-15	36
BP	aerobic respiration	GO:0009060	78	5.85e-12	28
BP	electron transport chain	GO:0022900	32	3.47e-11	18
BP	respiratory electron transport chain	GO:0022904	31	1.61e-11	18
BP	phosphorus metabolic process	GO:0006793	760	1.03e-06	89
BP	organophosphate metabolic process	GO:0019637	417	8.59e-14	73
BP	phosphate-containing compound metabolic process	GO:0006796	737	6.36e-05	82
BP	nucleoside phosphate metabolic process	GO:0006753	247	1.50e-23	67
BP	nucleoside monophosphate metabolic process	GO:0009123	135	8.78e-25	51
BP	ribonucleoside monophosphate metabolic process	GO:0009161	132	2.47e-25	51
BP	purine nucleoside monophosphate metabolic process	GO:0009126	116	1.69e-27	50
BP	purine ribonucleoside monophosphate metabolic process	GO:0009167	116	1.69e-27	50
BP	nucleoside phosphate biosynthetic process	GO:1901293	121	1.75e-02	21
BP	nucleoside monophosphate biosynthetic process	GO:0009124	61	1.88e-02	14
BP	purine nucleoside monophosphate biosynthetic process	GO:0009127	42	9.97e-04	13
BP	ribonucleoside monophosphate biosynthetic process	GO:0009156	58	1.00e-02	14
BP	purine ribonucleoside monophosphate biosynthetic process	GO:0009168	42	9.97e-04	13
BP	nucleotide metabolic process	GO:0009117	243	5.19e-24	67
BP	purine nucleotide metabolic process	GO:0006163	139	4.45e-25	52
BP	nucleotide biosynthetic process	GO:0009165	119	4.53e-02	20
BP	nucleoside triphosphate metabolic process	GO:0009141	70	1.62e-15	30
BP	ribonucleoside triphosphate metabolic process	GO:0009199	62	2.09e-17	30
BP	purine nucleoside triphosphate metabolic process	GO:0009144	60	9.66e-17	29
BP	purine ribonucleoside triphosphate metabolic process	GO:0009205	58	2.85e-17	29
BP	nucleoside triphosphate biosynthetic process	GO:0009142	29	6.95e-04	11
BP	ribonucleoside triphosphate biosynthetic process	GO:0009201	26	1.84e-04	11
BP	purine nucleoside triphosphate biosynthetic process	GO:0009145	22	3.01e-04	10
BP	purine ribonucleoside triphosphate biosynthetic process	GO:0009206	22	3.01e-04	10
BP	ribose phosphate metabolic process	GO:0019693	168	4.08e-26	58
BP	ribonucleotide metabolic process	GO:0009259	148	1.65e-25	54
BP	purine ribonucleotide metabolic process	GO:0009150	135	8.30e-26	52
BP	ATP metabolic process	GO:0046034	54	3.63e-17	28
BP	oxidative phosphorylation	GO:0006119	32	1.62e-12	19
BP	ATP synthesis coupled electron transport	GO:0042773	29	3.03e-12	18
BP	mitochondrial ATP synthesis coupled electron tran ...	GO:0042775	29	3.03e-12	18
BP	mitochondrial electron transport, cytochrome c ...	GO:0006123	12	2.40e-03	7
BP	mitochondrial electron transport, ubiquinol to ...	GO:0006122	11	1.05e-03	7
BP	thioester metabolic process	GO:0035383	20	1.58e-02	8
BP	organic acid metabolic process	GO:0006082	434	2.45e-23	90
BP	oxoacid metabolic process	GO:0043436	433	2.04e-23	90
BP	carboxylic acid metabolic process	GO:0019752	417	5.31e-24	89
BP	tricarboxylic acid metabolic process	GO:0072350	29	1.47e-09	16
BP	citrate metabolic process	GO:0006101	29	1.47e-09	16
BP	tricarboxylic acid cycle	GO:0006099	29	1.47e-09	16
BP	dicarboxylic acid metabolic process	GO:0043648	44	1.15e-02	12
BP	monocarboxylic acid metabolic process	GO:0032787	157	1.56e-18	48
BP	pyruvate metabolic process	GO:0006090	46	7.32e-10	20
BP	cellular amino acid metabolic process	GO:0006520	262	5.67e-03	36
BP	alpha-amino acid metabolic process	GO:1901605	195	3.63e-03	30
BP	glutamine family amino acid metabolic process	GO:0009064	61	1.88e-02	14
BP	organic acid catabolic process	GO:0016054	89	8.06e-03	18
BP	carboxylic acid catabolic process	GO:0046395	89	8.06e-03	18
BP	cofactor metabolic process	GO:0051186	206	8.77e-10	43
BP	coenzyme metabolic process	GO:0006732	182	2.14e-10	41
BP	oxidoreduction coenzyme metabolic process	GO:0006733	99	5.08e-09	28
BP	pyridine nucleotide metabolic process	GO:0019362	85	7.27e-11	28
BP	nicotinamide nucleotide metabolic process	GO:0046496	84	4.08e-10	27
BP	acyl-CoA metabolic process	GO:0006637	20	1.58e-02	8
BP	cofactor catabolic process	GO:0051187	9	4.61e-03	6
BP	coenzyme catabolic process	GO:0009109	8	4.99e-02	5
BP	cellular aldehyde metabolic process	GO:0006081	53	3.18e-03	14
BP	ribose phosphate biosynthetic process	GO:0046390	76	1.53e-02	16
BP	ribonucleotide biosynthetic process	GO:0009260	70	4.95e-03	16
BP	organic acid biosynthetic process	GO:0016053	191	6.95e-03	29
BP	carboxylic acid biosynthetic process	GO:0046394	191	6.95e-03	29
BP	cellular amino acid biosynthetic process	GO:0008652	144	2.83e-02	23
BP	alpha-amino acid biosynthetic process	GO:1901607	136	3.47e-02	22
BP	glutamine family amino acid biosynthetic process	GO:0009084	29	6.95e-04	11
BP	glutamate biosynthetic process	GO:0006537	11	2.28e-02	6
BP	purine-containing compound biosynthetic process	GO:0072522	70	9.98e-04	17
BP	purine nucleoside biosynthetic process	GO:0042451	48	1.23e-04	15
BP	purine ribonucleoside biosynthetic process	GO:0046129	48	1.23e-04	15
BP	purine nucleotide biosynthetic process	GO:0006164	60	1.53e-02	14
BP	purine ribonucleotide biosynthetic process	GO:0009152	57	8.07e-03	14
BP	ATP biosynthetic process	GO:0006754	19	7.91e-04	9
BP	carbohydrate catabolic process	GO:0016052	95	1.22e-05	23
BP	single-organism carbohydrate catabolic process	GO:0044724	82	9.90e-05	20
BP	glycolytic process	GO:0006096	32	2.10e-06	14
BP	hydrogen transport	GO:0006818	68	1.15e-04	18
BP	ion transport	GO:0006811	319	1.56e-04	45
BP	cation transport	GO:0006812	176	1.21e-02	27
BP	monovalent inorganic cation transport	GO:0015672	98	2.30e-05	23
BP	proton transport	GO:0015992	67	8.95e-05	18
BP	transmembrane transport	GO:0055085	407	1.64e-04	53
BP	ion transmembrane transport	GO:0034220	146	2.82e-04	27
BP	cation transmembrane transport	GO:0098655	94	4.52e-03	19
BP	inorganic ion transmembrane transport	GO:0098660	101	8.56e-04	21
BP	inorganic cation transmembrane transport	GO:0098662	88	1.59e-03	19
BP	hydrogen ion transmembrane transport	GO:1902600	57	5.31e-06	18
BP	energy coupled proton transport, down electrochemical gradient	GO:0015985	17	4.47e-02	7
BP	ATP synthesis coupled proton transport	GO:0015986	17	4.47e-02	7
BP	fatty acid metabolic process	GO:0006631	65	8.71e-03	15
BP	NADH metabolic process	GO:0006734	15	1.65e-02	7
BP	NADH oxidation	GO:0006116	10	1.09e-02	6

source	term name<br>Gene Ontology (Cellular component)	term ID	n. of term genes	corrected p-value	
CC	cell	GO:0005623	5339	5.72e-11	373
CC	cell part	GO:0044464	5339	5.72e-11	373
CC	cell periphery	GO:0071944	661	3.34e-07	82
CC	envelope	GO:0031975	552	2.10e-05	68
CC	external encapsulating structure	GO:0030312	106	1.10e-04	23
CC	cell wall	GO:0005618	106	1.10e-04	23
CC	fungal-type cell wall	GO:0009277	99	1.34e-04	22
CC	intracellular	GO:0005622	5073	1.24e-02	346
CC	intracellular part	GO:0044424	5062	8.22e-03	346
CC	cytoplasm	GO:0005737	4072	1.08e-15	328
CC	cytoplasmic part	GO:0044444	2797	8.11e-25	277
CC	cytosol	GO:0005829	573	3.23e-29	114
CC	cytosolic part	GO:0044445	220	2.23e-40	81
CC	mitochondrion	GO:0005739	1124	1.87e-14	138
CC	mitochondrial part	GO:0044429	603	9.28e-10	83
CC	ribonucleoprotein complex	GO:0030529	685	7.14e-12	95
CC	plasma membrane	GO:0005886	469	3.51e-07	65
CC	oxidoreductase complex	GO:1990204	36	1.26e-06	15
CC	cytochrome complex	GO:0070069	22	6.17e-08	13
CC	non-membrane-bounded organelle	GO:0043228	1276	4.16e-02	112
CC	intracellular non-membrane-bounded organelle	GO:0043232	1276	4.16e-02	112
CC	ribosome	GO:0005840	264	7.14e-31	78
CC	ribosomal subunit	GO:0044391	230	4.98e-30	72
CC	large ribosomal subunit	GO:0015934	131	1.25e-18	44
CC	small ribosomal subunit	GO:0015935	99	5.08e-09	28
CC	cytosolic ribosome	GO:0022626	161	5.85e-46	75
CC	cytosolic large ribosomal subunit	GO:0022625	86	1.17e-26	43
CC	cytosolic small ribosomal subunit	GO:0022627	64	1.21e-14	28
CC	proton-transporting ATP synthase complex	GO:0045259	17	4.47e-02	7
CC	organelle envelope	GO:0031967	552	2.10e-05	68
CC	organelle inner membrane	GO:0019866	254	2.72e-03	36
CC	mitochondrial envelope	GO:0005740	418	5.97e-09	64
CC	respiratory chain	GO:0070469	32	1.62e-12	19
CC	respiratory chain complex IV	GO:0045277	12	2.40e-03	7
CC	transmembrane transporter complex	GO:1902495	11	2.28e-02	6
CC	respiratory chain complex III	GO:0045275	10	1.09e-02	6
CC	organelle envelope lumen	GO:0031970	66	1.06e-02	15
CC	mitochondrial intermembrane space	GO:0005758	61	3.74e-03	15
CC	mitochondrial membrane	GO:0031966	379	2.57e-04	50
CC	mitochondrial membrane part	GO:0044455	168	9.86e-06	32
CC	mitochondrial inner membrane	GO:0005743	242	8.38e-04	36
CC	mitochondrial respiratory chain	GO:0005746	27	1.31e-11	17
CC	mitochondrial respiratory chain complex IV	GO:0005751	12	2.40e-03	7
CC	mitochondrial respiratory chain complex III	GO:0005750	10	1.09e-02	6
CC	mitochondrial proton-transporting ATP synthase complex	GO:0005753	17	4.47e-02	7
CC	extracellular region	GO:0005576	97	1.87e-05	23

source	term name<br>Gene Ontology (Molecular function)	term ID	n. of term genes	corrected p-value	
MF	electron carrier activity	GO:0009055	47	2.40e-02	12
MF	catalytic activity	GO:0003824	2171	2.07e-03	179
MF	oxidoreductase activity	GO:0016491	331	1.49e-11	60
MF	oxidoreductase activity, acting on CH-OH group of donors	GO:0016614	85	9.02e-04	19
MF	oxidoreductase activity, acting on the CH-OH group of donors, NAD ...	GO:0016616	82	2.34e-03	18
MF	oxidoreductase activity, acting on the aldehyde or oxo group of donors	GO:0016903	28	3.56e-02	9
MF	oxidoreductase activity, acting on the aldehyde or oxo group of do ...	GO:0016620	22	3.59e-02	8
MF	lyase activity	GO:0016829	108	3.67e-02	19
MF	structural molecule activity	GO:0005198	359	1.76e-22	80
MF	structural constituent of ribosome	GO:0003735	225	8.19e-30	71
MF	unfolded protein binding	GO:0051082	78	1.07e-03	18
MF	cofactor binding	GO:0048037	153	4.18e-08	34
MF	coenzyme binding	GO:0050662	103	4.86e-07	26
MF	NAD binding	GO:0051287	29	6.69e-05	12
MF	transporter activity	GO:0005215	414	8.51e-03	49
MF	substrate-specific transporter activity	GO:0022892	351	2.46e-03	45
MF	transmembrane transporter activity	GO:0022857	329	5.37e-05	47
MF	substrate-specific transmembrane transporter activity	GO:0022891	294	1.07e-04	43
MF	ion transmembrane transporter activity	GO:0015075	219	2.05e-04	35
MF	cation transmembrane transporter activity	GO:0008324	151	1.98e-03	26
MF	inorganic cation transmembrane transporter activity	GO:0022890	124	2.58e-02	21
MF	monovalent inorganic cation transmembrane transporter activity	GO:0015077	85	3.67e-05	21
MF	hydrogen ion transmembrane transporter activity	GO:0015078	60	8.77e-05	17
MF	transferase activity, transferring acyl groups, acyl groups converted in ...	GO:0046912	11	1.05e-03	7

source	term name<br>Biological pathways (KEGG)	term ID	n. of term genes	corrected p-value	
ke	2-Oxocarboxylic acid metabolism	KEGG:01210	35	1.52e-02	12
ke	Oxidative phosphorylation	KEGG:00190	72	4.70e-07	27
ke	Biosynthesis of secondary metabolites	KEGG:01110	275	1.98e-05	60
ke	Glycolysis / Gluconeogenesis	KEGG:00010	58	4.16e-05	21
ke	Carbon metabolism	KEGG:01200	111	1.72e-11	42
ke	Biosynthesis of antibiotics	KEGG:01130	217	2.82e-08	57
ke	Biosynthesis of amino acids	KEGG:01230	123	1.07e-04	33
ke	Citrate cycle (TCA cycle)	KEGG:00020	32	4.29e-06	16
ke	Ribosome	KEGG:03010	181	5.10e-21	70
ke	Propanoate metabolism	KEGG:00640	11	2.46e-02	6
ke	Metabolic pathways	KEGG:01100	687	3.18e-03	110
ke	Pyruvate metabolism	KEGG:00620	39	1.21e-04	16
ke	Glyoxylate and dicarboxylate metabolism	KEGG:00630	22	5.13e-05	12

source	term name<br>Biological pathways (Reactome)	term ID	n. of term genes	corrected p-value	
re	Metabolism of proteins	REAC:5719483	308	2.97e-11	88
re	Gene Expression	REAC:5719443	319	8.37e-05	76
re	Nonsense-Mediated Decay (NMD)	REAC:5719944	145	2.79e-21	68
re	Nonsense Mediated Decay (NMD) enhanced by the Exon Junction Complex ...	REAC:5719945	145	2.79e-21	68
re	Nonsense Mediated Decay (NMD) independent of the Exon Junction Compl ...	REAC:5719943	143	9.74e-22	68
re	Translation	REAC:5719482	164	2.04e-17	68
re	SRP-dependent cotranslational protein targeting to membrane	REAC:5719995	138	6.25e-23	68
re	Eukaryotic Translation Elongation	REAC:5719700	139	1.10e-22	68
re	Peptide chain elongation	REAC:5719699	139	1.10e-22	68
re	Eukaryotic Translation Initiation	REAC:5719481	155	3.81e-19	68
re	L13a-mediated translational silencing of Ceruloplasmin expression	REAC:5719697	148	1.29e-20	68
re	Cap-dependent Translation Initiation	REAC:5719480	154	2.39e-19	68
re	GTP hydrolysis and joining of the 60S ribosomal subunit	REAC:5719486	136	1.98e-23	68
re	Formation of the ternary complex, and subsequently, the 43S complex	REAC:5719484	66	7.55e-05	26
re	Formation of a pool of free 40S subunits	REAC:5719487	136	1.98e-23	68
re	Ribosomal scanning and start codon recognition	REAC:5719479	70	2.92e-04	26
re	Gluconeogenesis	REAC:5719427	27	2.74e-02	12
re	The citric acid (TCA) cycle and respiratory electron transport	REAC:5719463	36	1.06e-02	15
re	Pyruvate metabolism and Citric Acid (TCA) cycle	REAC:5719462	33	3.02e-03	15
re	Citric acid cycle (TCA cycle)	REAC:5719461	24	1.25e-04	14
re	Glycolysis	REAC:5719448	26	3.08e-03	13

source	term name<br>Regulatory motifs in DNA (TRANSFAC TFBS)	term ID	n. of term genes	corrected p-value	
tf	Factor: TBP; motif: YNTTTATAT; match class: 4	TF:M00713_4	2164	1.95e-03	166
tf	Factor: TBP; motif: YNTTTATAT; match class: 3	TF:M00713_3	2070	3.16e-03	159
tf	Factor: TBP; motif: YNTTTATAT; match class: 2	TF:M00713_2	1710	1.73e-02	132
tf	Factor: HSF; motif: AGAANNTTCTAGAAN; match class: 3	TF:M00169_3	264	2.62e-04	35
tf	Factor: HSF; motif: AGAANNTTCTAGAAN; match class: 1	TF:M00169_1	113	6.63e-06	23
tf	Factor: HSF; motif: AGAANNTTCTAGAAN; match class: 0	TF:M00169_0	42	1.53e-04	12
tf	Factor: RAP1; motif: WRMACCCATACAYY; match class: 4	TF:M00213_4	65	1.57e-02	12
