# Supplementary material for: Principal component analysis based unsupervised feature extraction applied to budding yeast temporally periodic gene expression
Source: BioData Min. 2016 Jun 29;9:22. doi: 10.1186/s13040-016-0101-9 (PMC4928327; doi:10.1186/s13040-016-0101-9)
Supplement: Additional file 3 — Document S1. Genes shown in Fig. 3. Black circles, red triangles, and green crosses are annotated as Cluster_1, Cluster_2, and Cluster_3, respectively. (PDF 19.2 kb) [file 13040_2016_101_MOESM3_ESM.pdf]

> culster 1
RPS25B RPS28B THI7 YEF3 CBF5 SAM1 SAM2 RPL37A FRS1 RPL22A RPS0B RPS0A RPL15A RPL15B RPL8A RPL8B RPS21A RPL14A RPL14B MAE1 TMA19 OAC1 RPL17A RPS5 RPL43B RPS21B RPL17B RPS22A EFB1 CDC19 RHR2 RPL34A RPL34B RPL16A GAR1 RPS27B RPL27A RPL24B NSR1 RPL11A RPL11B RPL7A RPS2 RPL2A RPL2B RPL29 MET6 FCY2 FCY22 PMP2 RPL12A HSP31 RPL37B RPL12B RPP2B RPS13 MRH1 RPS16B RPL13A RPP1B ARF1 LYS20 LYS21 NHP2 LEU2 RPL21A RPS9B PHO88 ECM33 RPL19A RPL19B RPL23A RPL23B RPS23B RPL21B RPL5 MF(ALPHA)1 RPL7B RPS12 NOP58 RPS10A RPS7A RPS15 RPP2A RPL18A RPL9A RPL9B YDJ1 DBP2 MEP2 AAH1 RPS3 SSB1 SSB2 RPL18B GAS1 GUA1 RPL36A RPL36B ASC1 RPS18A RPS18B

> culster 2
ATP14 ACO1 HSP60 COX12 COX17 SDH2 YNK1 MDM35 MDH1 SDH1 SDH3 XPT1 ATP2 MIR1 CYC1 CIS3 YHB1 MAM33 KGD1 SIM1 GND1 COX6 SCW4 MNP1 PYC1 HXK2 HXK1 HAC1 QCR6 AGX1 ICL1 RGI1 RIP1 QCR7 ITR1 TIM11 SDH4 ATP16 ARF2 DLD1 CIT2 AIM5 ECM33 ATP3 HTA1 HTA2 HTB2 HHT1 PET9 ACH1 QCR2 PDH1 FUM1 SRL1 IDH2 CYT1 TOM6 ADH1 CIT1 IDH1 HHT2 POR1 POR2 COX5A TOS6 SNZ2 SNZ3 NDE1 MIC17 MRPL39 TUB1 CPR3 NDI1

> culster 3
CTS1 TFS1 AHP1 PET10 FOX2 PIR1 JEN1 BAT2 TES1 YAT1 SSA1 SSA2 ACS1 RGI2 OM45 AYR1 POT1 DSE2 HXT5 CUP1-1 CUP1-2 GND2 SPG1 FMP43 SOL4 PIL1 ERG25 STF2 PNC1 HSP12 ARG5,6 YAT2 FMP52 ATO3 CTA1 CPR1 HSP42 FMP16 SED1 NDE2 HBT1 FMP45 ADY2 GLK1 APE3 OM14 SSE2 TKL2 TIP1 HSP26 YRO2 SSA3 NCE102 SUE1 LSP1 CIT3 ICL2 PMA2 SSU1 PEP4 GRE1 FDH1 FDH2 ALD4 FIT3 PUT4 CPA1 RDL1 WTM1 LSC1 LPX1 CRS5 STI1 DDR2 MDH2 HPF1 ATO2 PBI2 HEF3 YGP1 SPS19 ADH2 PRC1 PAI3 SIP18 SPG4 HFD1 CAT2 CYB2 MSC1
