# Supplementary material for: Principal component analysis based unsupervised feature extraction applied to budding yeast temporally periodic gene expression
Source: BioData Min. 2016 Jun 29;9:22. doi: 10.1186/s13040-016-0101-9 (PMC4928327; doi:10.1186/s13040-016-0101-9)
Supplement: Additional file 4 — Table S3. Enrichment analyses by g:profiler for the genes listed in document S1. (PDF 1361 kb) [file 13040_2016_101_MOESM4_ESM.pdf]

source	term name<br>Gene Ontology (Biological process)	term ID	n. of term genes	corrected p-value	CULSTER_1	CULSTER_2	CULSTER_3
BP	metabolic process	GO:0008152	4109	1.24e-03	78		
BP	organic substance metabolic process	GO:0071704	3723	1.27e-05	77		
BP	carbohydrate derivative metabolic process	GO:1901135	367	1.16e-11		26	
BP	macromolecule metabolic process	GO:0043170	2922	2.54e-05	67		
BP	gene expression	GO:0010467	1571	2.54e-14	60		
BP	organonitrogen compound metabolic process	GO:1901564	651	1.55e-11		33	
BP	pyridine-containing compound metabolic process	GO:0072524	100	1.69e-02		8	
BP	purine-containing compound metabolic process	GO:0072521	170	7.79e-19		25	
BP	amino-acid betaine metabolic process	GO:0006577	3	2.54e-03			3
BP	primary metabolic process	GO:0044238	3554	6.69e-07	77		
BP	protein metabolic process	GO:0019538	1543	6.53e-14	59		
BP	biosynthetic process	GO:0009058	2100	2.62e-12	66		
BP	organic substance biosynthetic process	GO:1901576	2070	1.17e-12	66		
BP	macromolecule biosynthetic process	GO:0009059	1559	3.94e-12	57		
BP	single-organism process	GO:0044699	3755	3.52e-04		63	
BP	single-organism metabolic process	GO:0044710	2051	9.14e-09		52	
BP	small molecule metabolic process	GO:0044281	789	3.18e-15		40	29
BP	single-organism catabolic process	GO:0044712	398	1.15e-02			18
BP	small molecule catabolic process	GO:0044282	113	5.16e-04			11
BP	glycosyl compound metabolic process	GO:1901657	165	3.61e-19		25	
BP	oxidation-reduction process	GO:0055114	437	8.27e-17		33	23
BP	cellular process	GO:0009987	4884	8.98e-03	84	69	
BP	single-organism cellular process	GO:0044763	3397	2.41e-04		60	
BP	nucleobase-containing small molecule metabolic process	GO:0055086	279	1.34e-14		26	
BP	nucleoside metabolic process	GO:0009116	161	1.92e-19		25	
BP	ribonucleoside metabolic process	GO:0009119	154	1.46e-18		24	
BP	purine nucleoside metabolic process	GO:0042278	140	1.35e-19		24	
BP	purine ribonucleoside metabolic process	GO:0046128	140	1.35e-19		24	
BP	cellular metabolic process	GO:0044237	3696	8.05e-06	77		
BP	generation of precursor metabolites and energy	GO:0006091	186	3.99e-29		33	
BP	energy derivation by oxidation of organic compounds	GO:0015980	152	1.79e-27		30	
BP	cellular respiration	GO:0045333	103	6.28e-28		27	
BP	aerobic respiration	GO:0009060	78	7.19e-23		22	
BP	electron transport chain	GO:0022900	32	6.47e-15		13	
BP	respiratory electron transport chain	GO:0022904	31	3.88e-15		13	
BP	phosphorus metabolic process	GO:0006793	760	2.74e-07		30	
BP	organophosphate metabolic process	GO:0019637	417	3.07e-12		28	
BP	phosphate-containing compound metabolic process	GO:0006796	737	3.50e-06		28	
BP	nucleoside phosphate metabolic process	GO:0006753	247	6.11e-16		26	
BP	nucleoside monophosphate metabolic process	GO:0009123	135	5.38e-20		24	
BP	ribonucleoside monophosphate metabolic process	GO:0009161	132	3.04e-20		24	
BP	ribonucleoside monophosphate biosynthetic process	GO:0009156	58	4.72e-02		6	
BP	purine nucleoside monophosphate metabolic process	GO:0009126	116	1.11e-21		24	
BP	purine ribonucleoside monophosphate metabolic process	GO:0009167	116	1.11e-21		24	
BP	purine nucleoside monophosphate biosynthetic process	GO:0009127	42	7.09e-03		6	
BP	purine ribonucleoside monophosphate biosynthetic process	GO:0009168	42	7.09e-03		6	
BP	nucleotide metabolic process	GO:0009117	243	4.02e-16		26	
BP	pyridine nucleotide metabolic process	GO:0019362	85	2.49e-02			8
BP	purine nucleotide metabolic process	GO:0006163	139	4.12e-21		25	
BP	nucleoside triphosphate metabolic process	GO:0009141	70	1.22e-20		20	
BP	ribonucleoside triphosphate metabolic process	GO:0009199	62	7.38e-22		20	
BP	purine nucleoside triphosphate metabolic process	GO:0009144	60	3.42e-22		20	
BP	purine ribonucleoside triphosphate metabolic process	GO:0009205	58	1.53e-22		20	
BP	nucleoside triphosphate biosynthetic process	GO:0009142	29	7.24e-04		6	
BP	ribonucleoside triphosphate biosynthetic process	GO:0009201	26	3.61e-04		6	
BP	purine nucleoside triphosphate biosynthetic process	GO:0009145	22	1.21e-04		6	
BP	purine ribonucleoside triphosphate biosynthetic process	GO:0009206	22	1.21e-04		6	
BP	phosphorylation	GO:0016310	338	1.91e-04		17	
BP	ribose phosphate metabolic process	GO:0019693	168	2.48e-20		26	
BP	ribonucleotide metabolic process	GO:0009259	148	2.14e-20		25	
BP	purine ribonucleotide metabolic process	GO:0009150	135	1.90e-21		25	
BP	ATP metabolic process	GO:0046034	54	1.80e-21		19	
BP	oxidative phosphorylation	GO:0006119	32	8.51e-17		14	
BP	ATP synthesis coupled electron transport	GO:0042773	29	1.30e-15		13	
BP	mitochondrial ATP synthesis coupled electron tran ...	GO:0042775	29	1.30e-15		13	
BP	mitochondrial electron transport, succinate to ...	GO:0006121	6	2.31e-04		4	
BP	mitochondrial electron transport, ubiquinol to ...	GO:0006122	11	8.31e-07		6	
BP	cellular macromolecule metabolic process	GO:0044260	2805	4.52e-05	65		
BP	cellular protein metabolic process	GO:0044267	1430	5.95e-14	57		
BP	organic acid metabolic process	GO:0006082	434	1.12e-06		21	25
BP	oxoacid metabolic process	GO:0043436	433	1.07e-06		21	25
BP	carboxylic acid metabolic process	GO:0019752	417	4.82e-07		21	25
BP	tricarboxylic acid metabolic process	GO:0072350	29	1.30e-15		13	
BP	citrate metabolic process	GO:0006101	29	1.30e-15		13	
BP	tricarboxylic acid cycle	GO:0006099	29	1.30e-15		13	
BP	dicarboxylic acid metabolic process	GO:0043648	44	5.49e-04		7	
BP	glutamate metabolic process	GO:0006536	15	1.94e-02		4	
BP	monocarboxylic acid metabolic process	GO:0032787	157	5.31e-09			18
BP	carnitine metabolic process	GO:0009437	3	2.54e-03			3
BP	organic acid catabolic process	GO:0016054	89	4.31e-05			11
BP	carboxylic acid catabolic process	GO:0046395	89	4.31e-05			11
BP	monocarboxylic acid catabolic process	GO:0072329	30	5.84e-06			8
BP	cellular biosynthetic process	GO:0044249	2030	3.87e-13	66		
BP	cellular macromolecule biosynthetic process	GO:0034645	1546	2.63e-12	57		
BP	translation	GO:0006412	476	1.97e-38	56		
BP	cytoplasmic translation	GO:0002181	171	2.86e-54	50		
BP	translational elongation	GO:0006414	100	1.17e-05	12		
BP	ribose phosphate biosynthetic process	GO:0046390	76	2.33e-02		7	
BP	ribonucleotide biosynthetic process	GO:0009260	70	1.35e-02		7	
BP	purine-containing compound biosynthetic process	GO:0072522	70	1.35e-02		7	
BP	purine nucleoside biosynthetic process	GO:0042451	48	1.57e-02		6	
BP	purine ribonucleoside biosynthetic process	GO:0046129	48	1.57e-02		6	
BP	purine nucleotide biosynthetic process	GO:0006164	60	4.77e-03		7	
BP	purine ribonucleotide biosynthetic process	GO:0009152	57	3.36e-03		7	
BP	ATP biosynthetic process	GO:0006754	19	1.77e-03		5	
BP	hydrogen transport	GO:0006818	68	3.87e-06		10	
BP	ion transport	GO:0006811	319	2.65e-03		15	
BP	cation transport	GO:0006812	176	4.59e-03		11	
BP	monovalent inorganic cation transport	GO:0015672	98	1.41e-04		10	
BP	proton transport	GO:0015992	67	3.33e-06		10	
BP	transmembrane transport	GO:0055085	407	5.38e-04		18	
BP	ion transmembrane transport	GO:0034220	146	7.25e-04		11	
BP	cation transmembrane transport	GO:0098655	94	9.46e-05		10	
BP	inorganic ion transmembrane transport	GO:0098660	101	1.59e-05		11	
BP	inorganic cation transmembrane transport	GO:0098662	88	4.99e-05		10	
BP	hydrogen ion transmembrane transport	GO:1902600	57	6.36e-07		10	
BP	energy coupled proton transport, down electrochemical gradient	GO:0015985	17	9.60e-04		5	
BP	ATP synthesis coupled proton transport	GO:0015986	17	9.60e-04		5	
BP	lipid catabolic process	GO:0016042	55	9.02e-04			8
BP	cellular lipid catabolic process	GO:0044242	40	6.81e-05			8
BP	fatty acid metabolic process	GO:0006631	65	2.13e-05			10
BP	fatty acid catabolic process	GO:0009062	18	1.13e-04			6
BP	glutamate biosynthetic process	GO:0006537	11	4.86e-03		4	
BP	regulation of translational fidelity	GO:0006450	24	1.65e-02	5		
BP	ribonucleoprotein complex biogenesis	GO:0022613	530	1.20e-08	30		
BP	ribosome biogenesis	GO:0042254	445	1.24e-10	30		
BP	ribosomal small subunit biogenesis	GO:0042274	137	2.02e-02	10		
BP	nuclear export	GO:0051168	126	9.63e-03	10		
BP	RNA export from nucleus	GO:0006405	90	3.43e-02	8		
BP	ncRNA metabolic process	GO:0034660	556	6.60e-03	22		
BP	ncRNA processing	GO:0034470	446	6.94e-04	21		
BP	rRNA metabolic process	GO:0016072	348	9.72e-06	21		
BP	rRNA processing	GO:0006364	332	4.18e-06	21		
BP	rRNA transport	GO:0051029	18	4.48e-08	8		
BP	rRNA export from nucleus	GO:0006407	18	4.48e-08	8		
BP	ribosome assembly	GO:0042255	59	1.63e-02	7		
BP	regulation of peptidase activity	GO:0052547	5	2.49e-02			3
BP	regulation of endopeptidase activity	GO:0052548	4	1.01e-02			3
BP	negative regulation of peptidase activity	GO:0010466	4	1.01e-02			3
BP	negative regulation of endopeptidase activity	GO:0010951	4	1.01e-02			3
BP	endonucleolytic cleavage to generate mature 3'-end of SSU-rRNA from (SSU ...	GO:0000461	5	1.74e-04	4		
BP	coenzyme metabolic process	GO:0006732	182	3.95e-02		10	

source	term name<br>Gene Ontology (Cellular component)	term ID	n. of term genes	corrected p-value	CULSTER_1	CULSTER_2	CULSTER_3
CC	cytochrome complex	GO:0070069	22	5.99e-08		8	
CC	nucleoid	GO:0009295	25	7.66e-03		5	
CC	envelope	GO:0031975	552	1.01e-12		32	
CC	macromolecular complex	GO:0032991	2124	3.91e-09	62		
CC	membrane-bounded organelle	GO:0043227	4076	2.07e-02		63	
CC	intracellular membrane-bounded organelle	GO:0043231	4066	1.84e-02		63	
CC	transporter complex	GO:1990351	27	1.14e-02		5	
CC	non-membrane-bounded organelle	GO:0043228	1276	2.68e-17	58		
CC	intracellular non-membrane-bounded organelle	GO:0043232	1276	2.68e-17	58		
CC	transmembrane transporter complex	GO:1902495	11	7.57e-05		5	
CC	organelle envelope	GO:0031967	552	1.01e-12		32	
CC	organelle inner membrane	GO:0019866	254	3.52e-11		22	
CC	organelle envelope lumen	GO:0031970	66	7.15e-04		8	
CC	respiratory chain	GO:0070469	32	6.47e-15		13	
CC	respiratory chain complex II	GO:0045273	6	2.31e-04		4	
CC	fumarate reductase complex	GO:0045283	5	7.76e-05		4	
CC	oxidoreductase complex	GO:1990204	36	2.28e-12		12	
CC	respiratory chain complex III	GO:0045275	10	4.17e-05		5	
CC	succinate dehydrogenase complex	GO:0045281	6	2.31e-04		4	
CC	succinate dehydrogenase complex (ubiquinone)	GO:0045257	5	7.76e-05		4	
CC	proton-transporting two-sector ATPase complex	GO:0016469	32	2.73e-02		5	
CC	proton-transporting ATP synthase complex	GO:0045259	17	9.60e-04		5	
CC	proton-transporting ATP synthase complex, catalytic core F(1)	GO:0045261	5	1.37e-02		3	
CC	cytoplasm	GO:0005737	4072	7.15e-04	78	63	78
CC	cytoplasmic part	GO:0044444	2797	3.24e-08	70	59	64
CC	microbody	GO:0042579	70	5.84e-03			8
CC	peroxisome	GO:0005777	70	5.84e-03			8
CC	cytosol	GO:0005829	573	2.20e-31	54		
CC	cytosolic part	GO:0044445	220	9.20e-50	51		
CC	tricarboxylic acid cycle enzyme complex	GO:0045239	7	5.34e-04		4	
CC	mitochondrion	GO:0005739	1124	6.45e-21		52	39
CC	mitochondrial part	GO:0044429	603	2.24e-25		45	22
CC	mitochondrial envelope	GO:0005740	418	2.52e-16		32	
CC	mitochondrial matrix	GO:0005759	216	1.25e-04		14	
CC	mitochondrial tricarboxylic acid cycle enzyme complex	GO:0030062	6	2.72e-02		3	
CC	mitochondrial intermembrane space	GO:0005758	61	3.84e-04		8	
CC	mitochondrial nucleoid	GO:0042645	25	7.66e-03		5	
CC	ribonucleoprotein complex	GO:0030529	685	4.88e-32	58		
CC	preribosome	GO:0030684	178	1.06e-03	13		
CC	90S preribosome	GO:0030686	92	4.95e-03	9		
CC	ribosome	GO:0005840	264	2.37e-45	51		
CC	ribosomal subunit	GO:0044391	230	2.11e-45	49		
CC	large ribosomal subunit	GO:0015934	131	6.79e-28	31		
CC	small ribosomal subunit	GO:0015935	99	9.44e-13	18		
CC	cytosolic ribosome	GO:0022626	161	8.55e-56	50		
CC	cytosolic large ribosomal subunit	GO:0022625	86	2.15e-34	31		
CC	cytosolic small ribosomal subunit	GO:0022627	64	1.98e-16	18		
CC	mitochondrial membrane	GO:0031966	379	2.52e-11		26	
CC	mitochondrial membrane part	GO:0044455	168	8.38e-14		21	
CC	mitochondrial inner membrane	GO:0005743	242	1.27e-11		22	
CC	mitochondrial respiratory chain	GO:0005746	27	3.44e-14		12	
CC	mitochondrial respiratory chain complex II, succinate dehydrogenas ...	GO:0005749	5	7.76e-05		4	
CC	mitochondrial respiratory chain complex III	GO:0005750	10	4.17e-05		5	
CC	mitochondrial proton-transporting ATP synthase complex	GO:0005753	17	9.60e-04		5	
CC	mitochondrial proton-transporting ATP synthase complex, catalytic ...	GO:0000275	5	1.37e-02		3	
CC	microbody lumen	GO:0031907	12	1.63e-02			4
CC	peroxisomal matrix	GO:0005782	12	1.63e-02			4
CC	cell periphery	GO:0071944	661	2.13e-05			29
CC	external encapsulating structure	GO:0030312	106	1.79e-02			9
CC	cell wall	GO:0005618	106	1.79e-02			9
CC	fungal-type cell wall	GO:0009277	99	1.02e-02			9
CC	plasma membrane	GO:0005886	469	5.39e-04			22
CC	extracellular region	GO:0005576	97	1.02e-03			10

source	term name<br>Gene Ontology (Molecular function)	term ID	n. of term genes	corrected p-value	CULSTER_1	CULSTER_2	CULSTER_3
MF	inorganic cation transmembrane transporter activity	GO:0022890	124	1.08e-02		9	
MF	monovalent inorganic cation transmembrane transporter activity	GO:0015077	85	4.52e-04		9	
MF	hydrogen ion transmembrane transporter activity	GO:0015078	60	2.05e-05		9	
MF	proton-transporting ATP synthase activity, rotational mechanism	GO:0046933	6	2.72e-02		3	
MF	RNA binding	GO:0003723	730	2.99e-05	30		
MF	rRNA binding	GO:0019843	130	1.79e-03	11		
MF	structural molecule activity	GO:0005198	359	2.20e-35	49		
MF	structural constituent of ribosome	GO:0003735	225	6.50e-46	49		
MF	peptidase inhibitor activity	GO:0030414	5	2.49e-02			3
MF	endopeptidase inhibitor activity	GO:0004866	5	2.49e-02			3
MF	succinate dehydrogenase activity	GO:0000104	7	4.73e-02		3	
MF	carnitine O-acyltransferase activity	GO:0016406	3	2.54e-03			3
MF	carnitine O-acetyltransferase activity	GO:0004092	3	2.54e-03			3
MF	cofactor binding	GO:0048037	153	1.16e-03		11	
MF	coenzyme binding	GO:0050662	103	2.10e-02		8	
MF	catalytic activity	GO:0003824	2171	1.52e-02		43	
MF	oxidoreductase activity	GO:0016491	331	2.31e-05		18	17
MF	oxidoreductase activity, acting on the aldehyde or oxo group of donors	GO:0016903	28	3.81e-02			5
MF	oxidoreductase activity, acting on the aldehyde or oxo group of do ...	GO:0016620	22	1.09e-02			5
MF	transmembrane transporter activity	GO:0022857	329	1.88e-02		14	
MF	substrate-specific transmembrane transporter activity	GO:0022891	294	2.60e-02		13	
MF	ion transmembrane transporter activity	GO:0015075	219	6.41e-03		12	
MF	electron carrier activity	GO:0009055	47	8.77e-04		7	

source	term name<br>Human Phenotype Ontology	term ID	n. of term genes	corrected p-value	CULSTER_1	CULSTER_2	CULSTER_3
hp	Triphalangeal thumb	HP:0001199	15	4.24e-03	5		
hp	Abnormality of lower lip	HP:0000178	75	4.04e-02	8		
hp	Thick vermilion border	HP:0012471	62	9.43e-03	8		
hp	Thick lower lip vermilion	HP:0000179	59	6.40e-03	8		
hp	Abnormality of acid-base homeostasis	HP:0004360	129	4.50e-02		11	
hp	Acidosis	HP:0001941	122	2.60e-02		11	
hp	Neoplasm	HP:0002664	167	1.22e-02	12		
hp	Neoplasm by anatomical site	HP:0011793	164	1.00e-02	12		
hp	Abnormality of the immune system	HP:0002715	275	9.36e-03	15		
hp	Abnormality of cellular immune system	HP:0010987	138	1.44e-03	12		
hp	Abnormality of blood and blood-forming tissues	HP:0001871	304	3.77e-02	15		
hp	Abnormality of bone marrow cell morphology	HP:0005561	182	3.13e-02	12		
hp	Abnormality of cells of the erythroid lineage	HP:0012130	173	1.80e-02	12		
hp	Abnormality of erythrocytes	HP:0001877	173	1.80e-02	12		
hp	Anemia	HP:0001903	162	8.73e-03	12		
hp	Anemia of inadequate production	HP:0010972	67	1.72e-02	8		
hp	Macrocytic anemia	HP:0001972	40	2.76e-04	8		
hp	Erythrocyte macrocytosis	HP:0005518	7	3.16e-03	4		
hp	Abnormality of leukocytes	HP:0001881	138	1.44e-03	12		
hp	Hematological neoplasm	HP:0004377	91	1.07e-05	12		
hp	Leukemia	HP:0001909	63	5.48e-05	10		
hp	Acute leukemia	HP:0002488	50	4.99e-06	10		
hp	Hypoketotic hypoglycemia	HP:0001985	4	1.11e-02			3
hp	Pallor	HP:0000980	40	2.76e-04	8		
hp	Headache	HP:0002315	53	2.74e-03	8		
hp	Migraine	HP:0002076	44	6.08e-04	8		
hp	Abnormality of the nasal dorsum	HP:0011119	72	2.97e-02	8		
hp	Depressed nasal ridge	HP:0000457	46	8.75e-04	8		

source	term name<br>Biological pathways (KEGG)	term ID	n. of term genes	corrected p-value	CULSTER_1	CULSTER_2	CULSTER_3
ke	Oxidative phosphorylation	KEGG:00190	72	3.69e-14		18	
ke	2-Oxocarboxylic acid metabolism	KEGG:01210	35	2.60e-02		5	
ke	Peroxisome	KEGG:04146	38	1.49e-03			6
ke	Carbon metabolism	KEGG:01200	111	1.24e-10		18	10
ke	Fatty acid degradation	KEGG:00071	19	9.00e-03			4
ke	Citrate cycle (TCA cycle)	KEGG:00020	32	5.44e-13		13	
ke	Pyruvate metabolism	KEGG:00620	39	1.78e-02		5	5
ke	Biosynthesis of secondary metabolites	KEGG:01110	275	8.45e-05		18	17
ke	Biosynthesis of antibiotics	KEGG:01130	217	9.99e-06		18	16
ke	Metabolic pathways	KEGG:01100	687	6.05e-07		34	
ke	Glyoxylate and dicarboxylate metabolism	KEGG:00630	22	1.57e-04		6	5
ke	Valine, leucine and isoleucine degradation	KEGG:00280	13	1.81e-03			4
ke	Ribosome	KEGG:03010	181	3.84e-35	49		

source	term name<br>Biological pathways (Reactome)	term ID	n. of term genes	corrected p-value	CULSTER_1	CULSTER_2	CULSTER_3
re	Metabolism of proteins	REAC:5719483	308	1.93e-19	47		
re	Mitochondrial protein import	REAC:5719964	59	4.81e-09		14	
re	Gene Expression	REAC:5719443	319	1.05e-18	47		
re	Nonsense-Mediated Decay (NMD)	REAC:5719944	145	2.42e-36	47		
re	Nonsense Mediated Decay (NMD) enhanced by the Exon Junction Complex ...	REAC:5719945	145	2.42e-36	47		
re	Nonsense Mediated Decay (NMD) independent of the Exon Junction Compl ...	REAC:5719943	143	1.12e-36	47		
re	Translation	REAC:5719482	164	2.06e-33	47		
re	SRP-dependent cotranslational protein targeting to membrane	REAC:5719995	138	1.52e-37	47		
re	Eukaryotic Translation Elongation	REAC:5719700	139	2.28e-37	47		
re	Peptide chain elongation	REAC:5719699	139	2.28e-37	47		
re	Eukaryotic Translation Initiation	REAC:5719481	155	9.59e-35	47		
re	L13a-mediated translational silencing of Ceruloplasmin expression	REAC:5719697	148	7.54e-36	47		
re	Cap-dependent Translation Initiation	REAC:5719480	154	6.72e-35	47		
re	GTP hydrolysis and joining of the 60S ribosomal subunit	REAC:5719486	136	6.68e-38	47		
re	Formation of the ternary complex, and subsequently, the 43S complex	REAC:5719484	66	9.36e-08	17		
re	Formation of a pool of free 40S subunits	REAC:5719487	136	6.68e-38	47		
re	Ribosomal scanning and start codon recognition	REAC:5719479	70	2.57e-07	17		
re	Bile acid and bile salt metabolism	REAC:5719762	5	9.26e-03			3
re	Synthesis of bile acids and bile salts	REAC:5719761	5	9.26e-03			3
re	Cellular responses to stress	REAC:5719471	44	2.09e-02			6
re	Peroxisomal lipid metabolism	REAC:5719850	11	5.18e-03			4
re	Beta-oxidation of pristanoyl-CoA	REAC:5719857	4	3.75e-03			3
re	Beta-oxidation of very long chain fatty acids	REAC:5719863	6	2.53e-04			4
re	Attenuation phase	REAC:5720061	6	1.83e-02			3
re	Regulation of HSF1-mediated heat shock response	REAC:5720051	18	4.36e-02			4
re	Metabolism	REAC:5719430	431	2.00e-02			18
re	The citric acid (TCA) cycle and respiratory electron transport	REAC:5719463	36	5.26e-08		11	
re	Pyruvate metabolism and Citric Acid (TCA) cycle	REAC:5719462	33	1.78e-08		11	
re	Citric acid cycle (TCA cycle)	REAC:5719461	24	2.68e-10		11	

source	term name<br>Regulatory motifs in DNA (TRANSFAC TFBS)	term ID	n. of term genes	corrected p-value	CULSTER_1	CULSTER_2	CULSTER_3
tf	Factor: TAF; motif: CGTNNRAAAYGACHA; match class: 4	TF:M00703_4	131	1.62e-02		7	
tf	Factor: TAF; motif: CGTNNRAAAYGACHA; match class: 3	TF:M00703_3	102	2.48e-02		6	
tf	Factor: RAP1; motif: WRMACCCATACAYY; match class: 4	TF:M00213_4	65	5.81e-06	9		
tf	Factor: RAP1; motif: WRMACCCATACAYY; match class: 3	TF:M00213_3	47	5.51e-06	8		
tf	Factor: ABF1; motif: NNWNRTCAYTNTANACGDANNN; match class: 4	TF:M00015_4	508	3.92e-02	16		
tf	Factor: TBP; motif: YNTTTATAT; match class: 4	TF:M00713_4	2164	5.75e-03			47
tf	Factor: TBP; motif: YNTTTATAT; match class: 3	TF:M00713_3	2070	4.01e-03			46
tf	Factor: TBP; motif: YNTTTATAT; match class: 2	TF:M00713_2	1710	2.29e-03			41
tf	Factor: TBP; motif: YNTTTATAT; match class: 1	TF:M00713_1	1651	5.79e-03			39
tf	Factor: TBP; motif: YNTTTATAT; match class: 0	TF:M00713_0	1207	2.18e-02			30
tf	Factor: HSF; motif: AGAANNTTCTAGAAN; match class: 3	TF:M00169_3	264	2.16e-03			13
tf	Factor: HSF; motif: AGAANNTTCTAGAAN; match class: 1	TF:M00169_1	113	7.76e-04			9
tf	Factor: ABF1; motif: NNWNRTCAYTNTANACGDANNN; match class: 2	TF:M00015_2	155	3.59e-02	8		
