# Supplementary material for: Principal component analysis based unsupervised feature extraction applied to budding yeast temporally periodic gene expression
Source: BioData Min. 2016 Jun 29;9:22. doi: 10.1186/s13040-016-0101-9 (PMC4928327; doi:10.1186/s13040-016-0101-9)
Supplement: Additional file 5 — Figure S1. Winding numbers and scatter plots of seven YCDC data sets. (PDF 61.7 kb) [file 13040_2016_101_MOESM5_ESM.pdf]

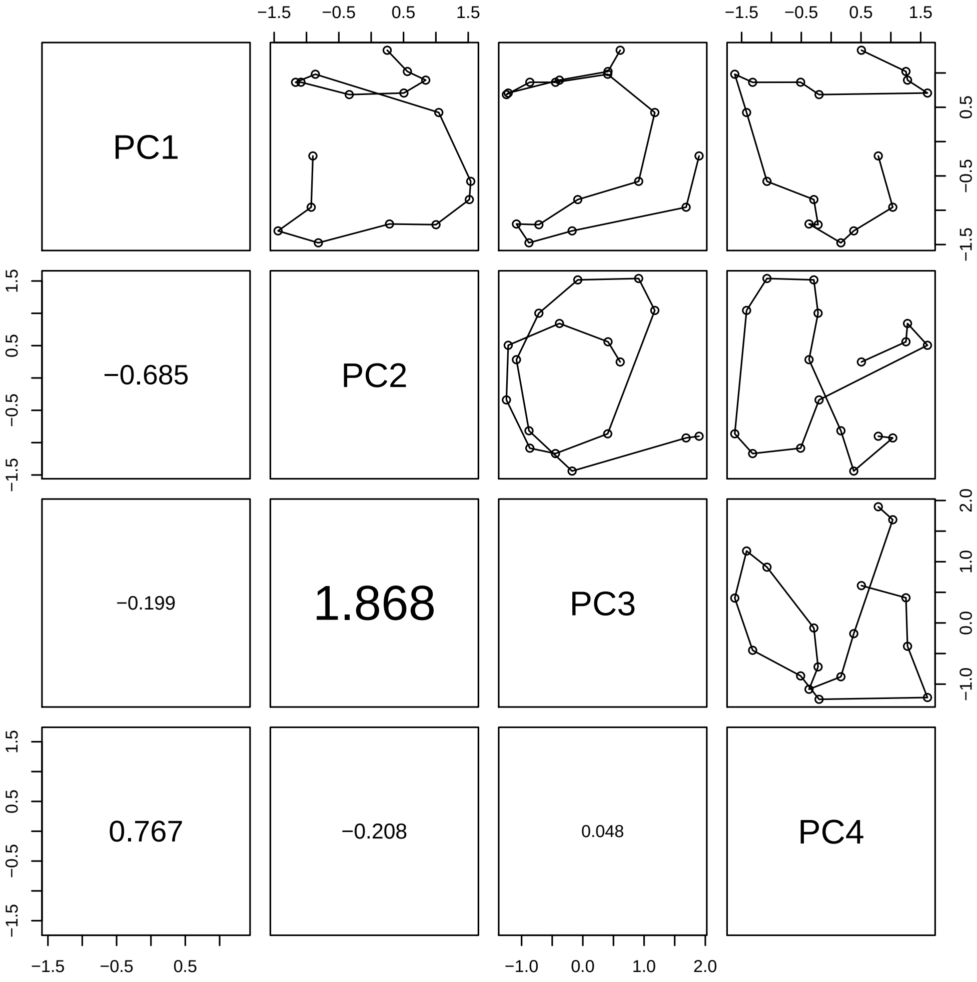

Fig. S1A: Cho et al [1]: G1 phase arrest by temperature-sensitive cdc28-13 mutant cells, PC2 and PC3 are employed for FE.

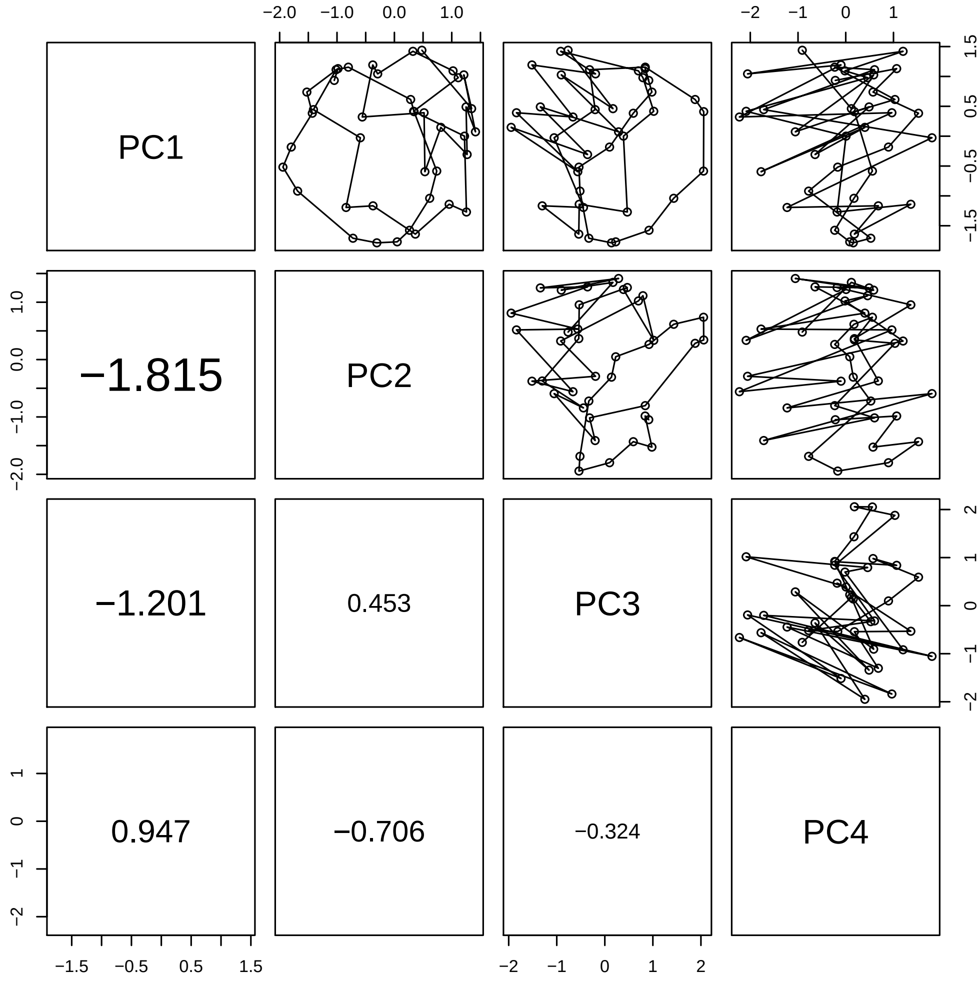

Fig. S1B: Granovskaia et al [2] : G1 phase arrest by α-factor, PC1 and PC2 are employed for FE.

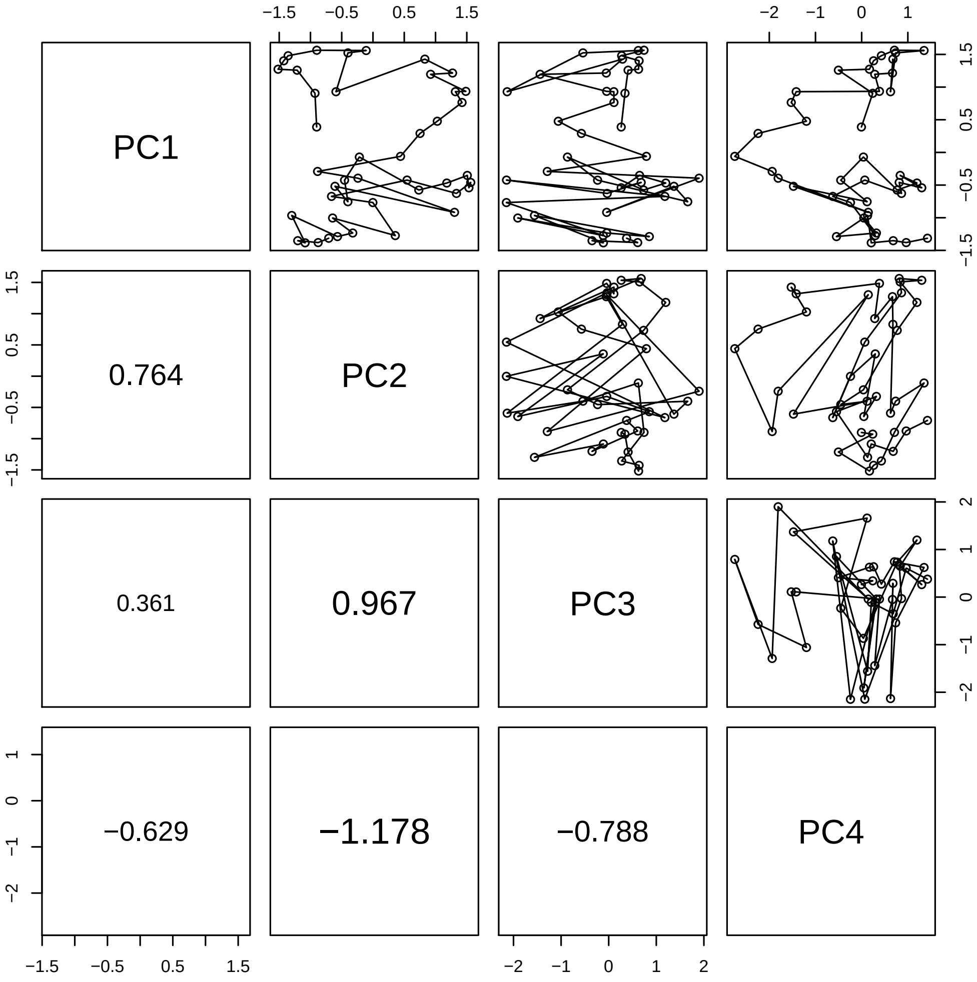

Fig. S1C: Granovskaia et al [2] : G1 phase arrest by temperature-sensitive cdc28-13 mutant cells, PC2 and PC4 are employed for FE.

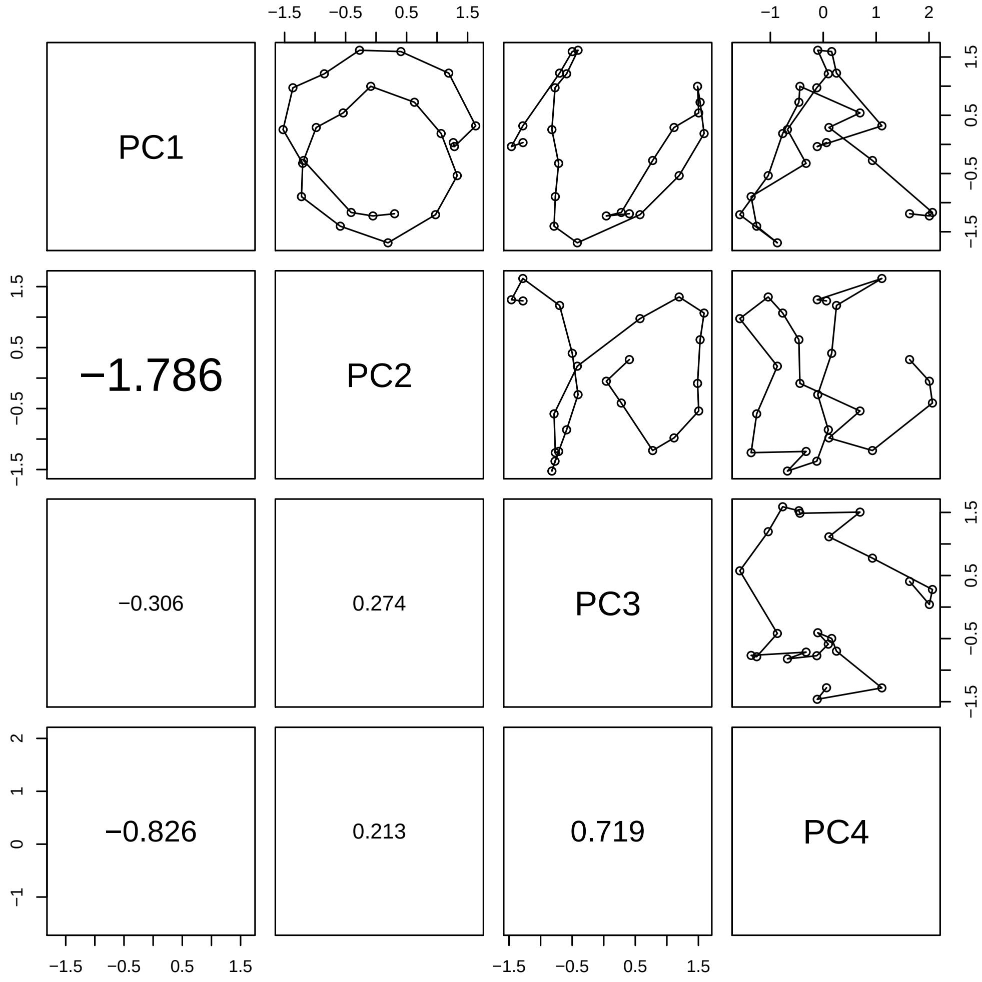

Fig. S1D: Pramila et al [3] : α-Factor synchronization: α30 data, PC1 and PC2 are employed for FE.

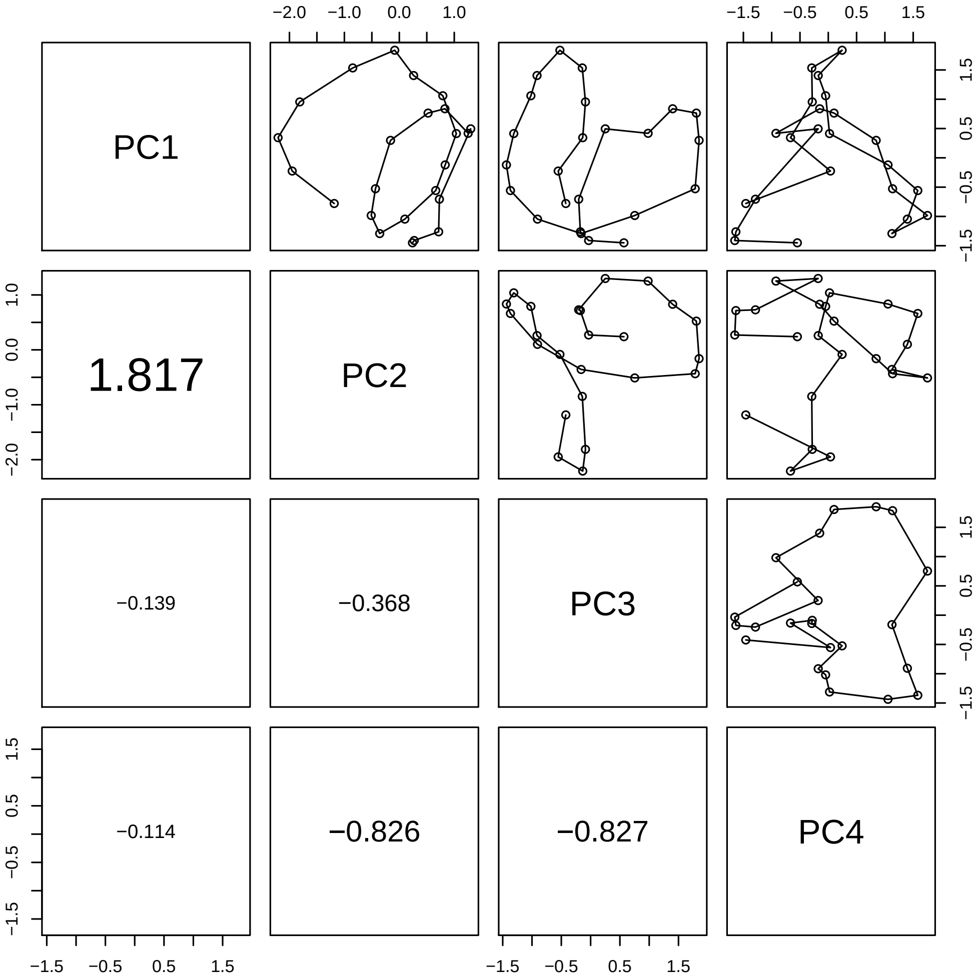

Fig. S1E: Pramila et al [3] : α-Factor synchronization: α38 data, PC1 and PC2 are employed for FE.

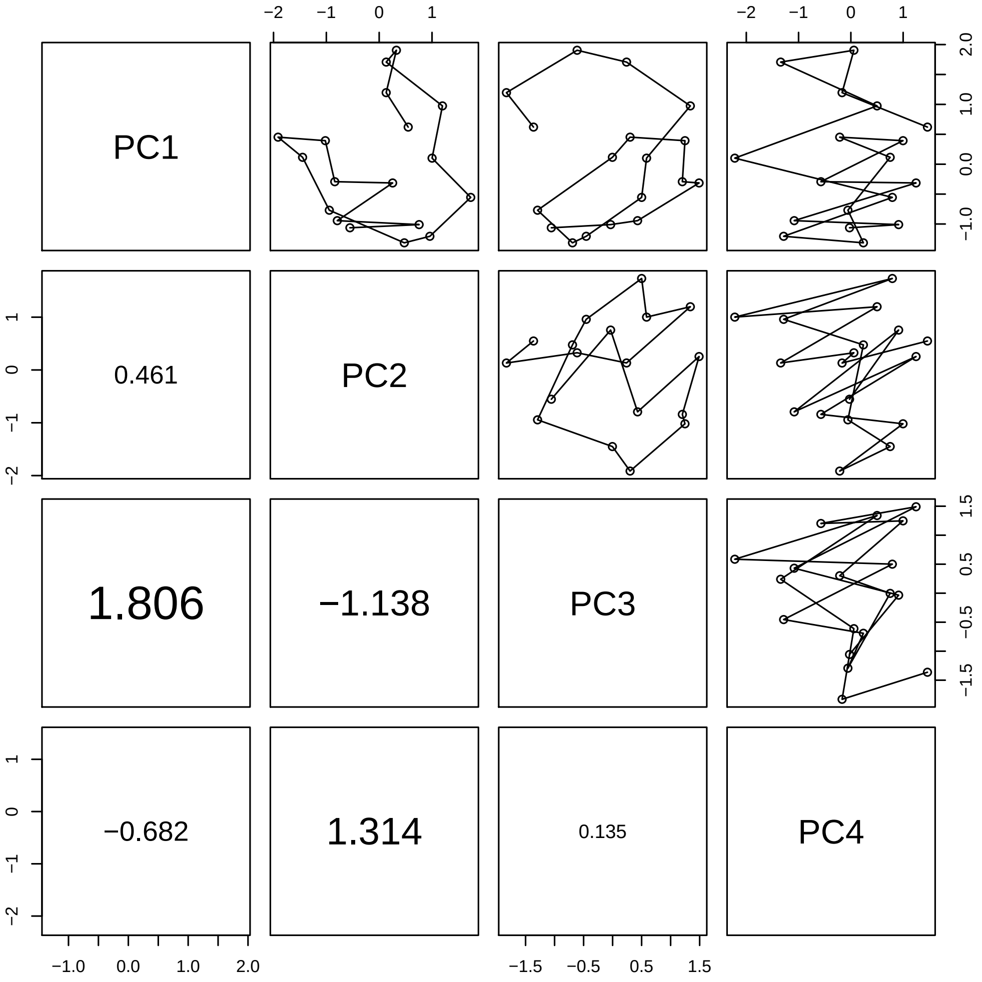

Fig. S1F: Spellman et al [4] : α factor arrest, PC1 and PC3 are employed for FE.

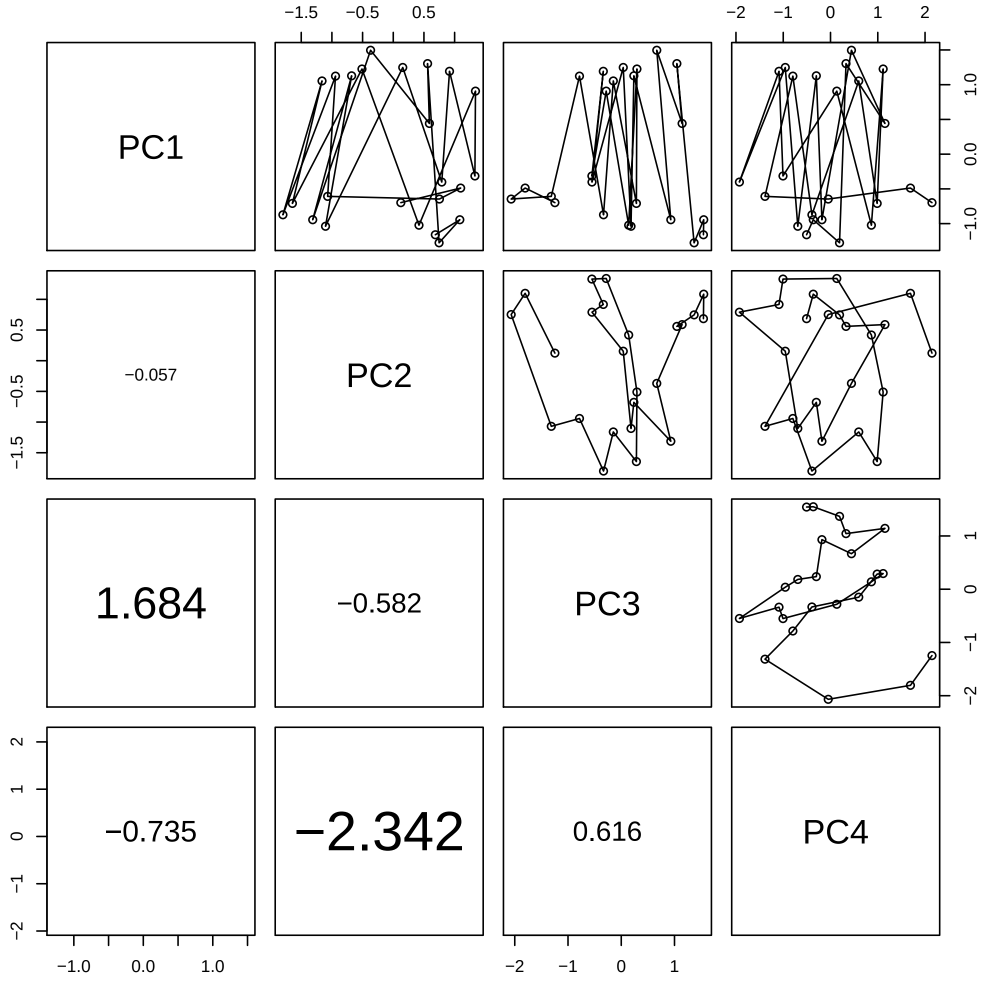

Fig. S1G: Spellman et al [4] : arrest of a cdc15 temperature-sensitive mutant, PC2 and PC4 are employed for FE.

References

[1] R. J. Cho, M. J. Campbell, E. A. Winzeler, L. Steinmetz, A. Conway, L. Wodicka, T. G. Wolfsberg, A. E. Gabrielian, D. Landsman, D. J. Lockhart, and R. W. Davis. A genome-wide transcriptional analysis of the mitotic cell cycle. *Mol. Cell*, 2(1):65–73, Jul 1998.

[2] M. V. Granovskaia, L. J. Jensen, M. E. Ritchie, J. Toedling, Y. Ning, P. Bork, W. Huber, and L. M. Steinmetz. High-resolution transcription atlas of the mitotic cell cycle in budding yeast. *Genome Biol.*, 11(3):R24, 2010.

[3] T. Pramila, W. Wu, S. Miles, W. S. Noble, and L. L. Breeden. The Forkhead transcription factor Hcm1 regulates chromosome segregation genes and fills the S-phase gap in the transcriptional circuitry of the cell cycle. *Genes Dev.*, 20(16):2266–2278, Aug 2006.

[4] P. T. Spellman, G. Sherlock, M. Q. Zhang, V. R. Iyer, K. Anders, M. B. Eisen, P. O. Brown, D. Botstein, and B. Futcher. Comprehensive identification of cell cycle-regulated genes of the yeast Saccharomyces cerevisiae by microarray hybridization. *Mol. Biol. Cell*, 9(12):3273–3297, Dec 1998.
