# Supplementary material for: Principal component analysis based unsupervised feature extraction applied to budding yeast temporally periodic gene expression
Source: BioData Min. 2016 Jun 29;9:22. doi: 10.1186/s13040-016-0101-9 (PMC4928327; doi:10.1186/s13040-016-0101-9)
Supplement: Additional file 7 — Table S5. Enrichment analyses by g:profiler for the genes listed in Table S1J (PCA) and S1K (CYCLEBASE). (PDF 433 kb) [file 13040_2016_101_MOESM7_ESM.pdf]

source	term name<br>Gene Ontology (Biological process)	term ID	n. of term genes	corrected p-value	PCA	CYCLEBASE
BP	cell division	GO:0051301	355	5.06e-07	15	
BP	regulation of spindle pole body separation	GO:0010695	9	1.83e-02	3	
BP	positive regulation of spindle pole body separation	GO:0010696	7	7.68e-03	3	
BP	chromatin assembly or disassembly	GO:0006333	67	9.19e-03		5
BP	reproduction	GO:0000003	486	3.16e-04	14	
BP	protein phosphorylation	GO:0006468	213	3.28e-03	9	
BP	anatomical structure homeostasis	GO:0060249	78	1.94e-02		5
BP	organelle organization	GO:0006996	1491	3.82e-03	22	
BP	organelle fission	GO:0048285	351	5.02e-06	14	
BP	nuclear division	GO:0000280	341	3.44e-06	14	
BP	single-organism organelle organization	GO:1902589	1007	4.15e-03	18	
BP	chromosome organization	GO:0051276	523	9.34e-07	13	15
BP	telomere organization	GO:0032200	79	2.07e-02		5
BP	regulation of protein kinase activity	GO:0045859	65	4.10e-02	5	
BP	regulation of cyclin-dependent protein kinase activity	GO:1904029	32	1.15e-03	5	
BP	regulation of protein serine/threonine kinase activity	GO:0071900	43	5.25e-03	5	
BP	cell cycle	GO:0007049	741	4.10e-10	23	12
BP	cell cycle process	GO:0022402	629	2.65e-08	20	12
BP	meiosis I	GO:0007127	98	1.53e-03	7	
BP	mitotic cell cycle	GO:0000278	379	7.91e-09	17	9
BP	mitotic cell cycle process	GO:1903047	367	6.51e-08	16	9
BP	mitotic nuclear division	GO:0007067	215	2.75e-05	11	
BP	regulation of cell cycle	GO:0051726	214	3.41e-03	9	
BP	regulation of cyclin-dependent protein serine/threonine kinase activity	GO:0000079	32	1.15e-03	5	
BP	cellular response to DNA damage stimulus	GO:0006974	331	2.53e-04	12	10
BP	DNA metabolic process	GO:0006259	542	1.75e-05	15	14
BP	regulation of DNA metabolic process	GO:0051052	107	3.85e-02	6	
BP	DNA recombination	GO:0006310	241	6.94e-03		8
BP	maintenance of DNA repeat elements	GO:0043570	23	2.00e-04	5	4
BP	DNA repair	GO:0006281	284	2.41e-03	9	9
BP	telomere maintenance	GO:0000723	78	1.94e-02		5
BP	telomere maintenance via recombination	GO:0000722	34	1.23e-02		4
BP	meiotic mismatch repair	GO:0000710	9	1.83e-02	3	

source	term name<br>Gene Ontology (Cellular component)	term ID	n. of term genes	corrected p-value	PCA	CYCLEBASE
CC	cellular bud	GO:0005933	219	4.13e-03	9	
CC	cellular bud neck	GO:0005935	168	5.31e-03	8	
CC	DNA bending complex	GO:1990104	12	8.98e-07	3	5
CC	protein-DNA complex	GO:0032993	61	2.06e-04		6
CC	DNA packaging complex	GO:0044815	17	6.90e-06		5
CC	chromosome	GO:0005694	387	3.99e-04		11
CC	chromosomal part	GO:0044427	351	1.53e-03		10
CC	replication fork	GO:0005657	56	3.19e-06		7
CC	chromatin	GO:0000785	139	2.60e-02		6
CC	nucleosome	GO:0000786	12	8.98e-07	3	5
CC	nuclear chromosome	GO:0000228	275	1.60e-04	9	10
CC	nuclear chromosome part	GO:0044454	248	7.77e-04		9
CC	nuclear replication fork	GO:0043596	44	2.76e-05		6
CC	nuclear chromatin	GO:0000790	117	9.72e-03		6
CC	replication fork protection complex	GO:0031298	27	8.73e-05		5
CC	nuclear nucleosome	GO:0000788	9	1.44e-07	3	5

source	term name<br>Gene Ontology (Molecular function)	term ID	n. of term genes	corrected p-value	PCA	CYCLEBASE
MF	protein heterodimerization activity	GO:0046982	33	1.09e-02		4
MF	kinase regulator activity	GO:0019207	53	1.50e-02	5	
MF	protein kinase regulator activity	GO:0019887	51	1.24e-02	5	
MF	cyclin-dependent protein serine/threonine kinase regulator activity	GO:0016538	26	3.85e-04	5	

source	term name<br>Biological pathways (KEGG)	term ID	n. of term genes	corrected p-value	PCA	CYCLEBASE
ke	Cell cycle - yeast	KEGG:04111	126	1.67e-08	12	5
ke	Mismatch repair	KEGG:03430	20	1.01e-03	4	3

source	term name<br>Biological pathways (Reactome)	term ID	n. of term genes	corrected p-value	PCA	CYCLEBASE
re	Lagging Strand Synthesis	REAC:5719416	16	1.97e-02	3	2
re	Processive synthesis on the lagging strand	REAC:5719417	11	5.93e-03	3	2
re	Removal of the Flap Intermediate	REAC:5719418	11	5.93e-03	3	2
re	Cell Cycle	REAC:5719402	113	5.33e-04	7	
re	Cell Cycle, Mitotic	REAC:5719401	101	2.44e-04	7	
re	M Phase	REAC:5719693	22	1.53e-03	4	
re	Mitotic Prometaphase	REAC:5720035	16	1.97e-02	3	
re	Condensation of Prometaphase Chromosomes	REAC:5720034	16	1.97e-02	3	
re	Mitotic Prophase	REAC:5720016	11	7.25e-05	4	
re	Nuclear Envelope Breakdown	REAC:5720063	8	2.04e-03	3	
re	Depolymerisation of the Nuclear Lamina	REAC:5720062	8	2.04e-03	3	
re	Mitotic G1-G1/S phases	REAC:5719408	21	1.26e-03	4	
re	G1/S Transition	REAC:5719407	21	1.26e-03	4	
re	E2F mediated regulation of DNA replication	REAC:5719680	19	8.23e-04	4	
re	E2F-enabled inhibition of pre-replication complex formation	REAC:5719681	8	2.04e-03	3	
re	Mitotic G2-G2/M phases	REAC:5719696	14	2.17e-04	4	
re	G2/M Transition	REAC:5719695	14	2.17e-04	4	
re	Cyclin A/B1 associated events during G2/M transition	REAC:5719730	14	2.17e-04	4	
re	Cyclin B2 mediated events	REAC:5719732	8	2.04e-03	3	
re	Regulation of mitotic cell cycle	REAC:5719736	9	3.05e-03	3	
re	APC/C-mediated degradation of cell cycle proteins	REAC:5719735	9	3.05e-03	3	
re	Regulation of APC/C activators between G1/S and early anaphase	REAC:5719737	8	2.04e-03	3	
re	DNA Repair	REAC:5719501	66	8.63e-03		3
re	Mismatch Repair	REAC:5720068	8	2.04e-03	3	2
re	Mismatch repair (MMR) directed by MSH2:MSH6 (MutSalpha)	REAC:5720067	7	1.28e-03	3	2
re	DNA Damage Bypass	REAC:5719649	19	3.69e-02		2
re	Translesion synthesis by Y family DNA polymerases bypasses lesions o ...	REAC:5719648	19	3.69e-02		2
re	Translesion synthesis by POLK	REAC:5720079	14	1.97e-02		2
re	Termination of translesion DNA synthesis	REAC:5720077	15	2.27e-02		2
re	Translesion synthesis by REV1	REAC:5719647	13	1.69e-02		2
re	Translesion synthesis by POLI	REAC:5720080	14	1.97e-02		2
re	Translesion Synthesis by POLH	REAC:5719650	16	2.59e-02		2
re	G2/M Checkpoints	REAC:5719425	14	1.29e-02	3	
re	G2/M DNA replication checkpoint	REAC:5719731	8	2.04e-03	3	

source	term name<br>Regulatory motifs in DNA (TRANSFAC TFBS)	term ID	n. of term genes	corrected p-value	PCA	CYCLEBASE
tf	Factor: MCM1; motif: WTTCCYAAWNNGGTAA; match class: 4	TF:M00125_4	198	8.38e-06	9	
tf	Factor: MCM1; motif: WTTCCYAAWNNGGTAA; match class: 3	TF:M00125_3	161	2.86e-04	7	
tf	Factor: STE11; motif: AGAACAAAGAAA; match class: 0	TF:M01005_0	17	4.39e-02		2
tf	Factor: MCM1+SFF; motif: TTWCCYAAWNNGGAAAWWNGTAAAYAA; match class: 4	TF:M01051_4	26	6.86e-03	3	
