# Supplementary figures and images for: Principal component analysis based unsupervised feature extraction applied to budding yeast temporally periodic gene expression
Source: BioData Min. 2016 Jun 29;9:22. doi: 10.1186/s13040-016-0101-9 (PMC4928327; doi:10.1186/s13040-016-0101-9)

**(A):  $A = 1$** 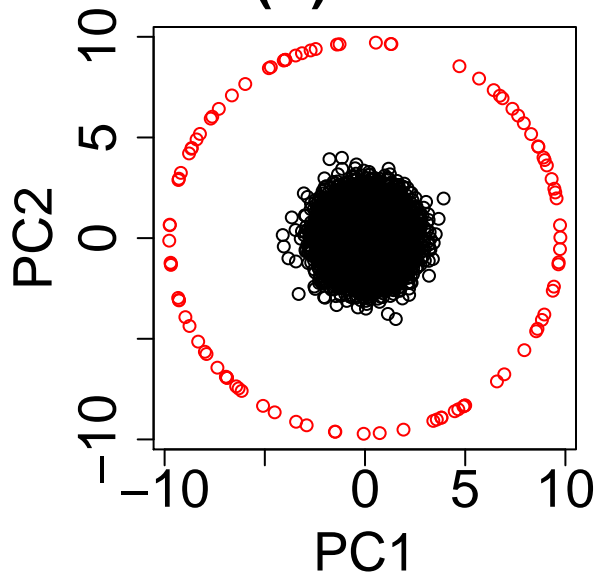**(B)**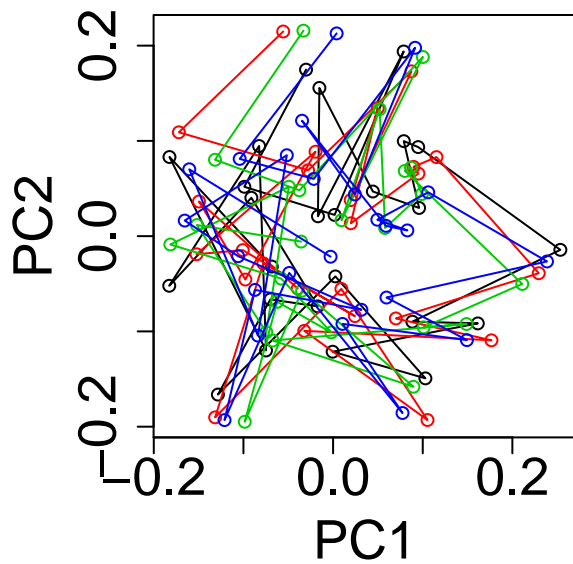**(C)**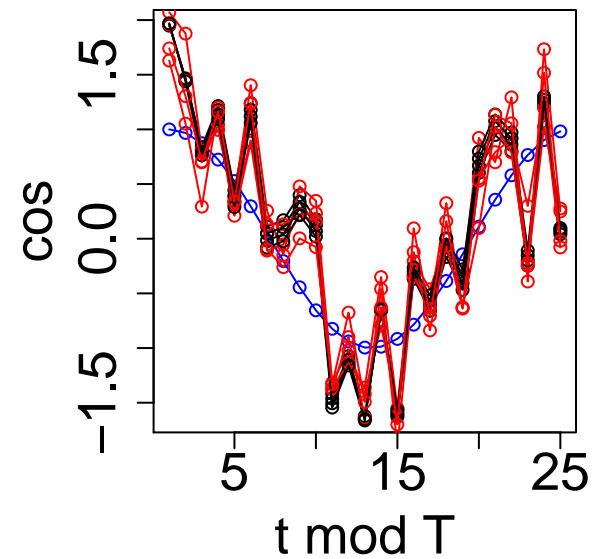**(D)**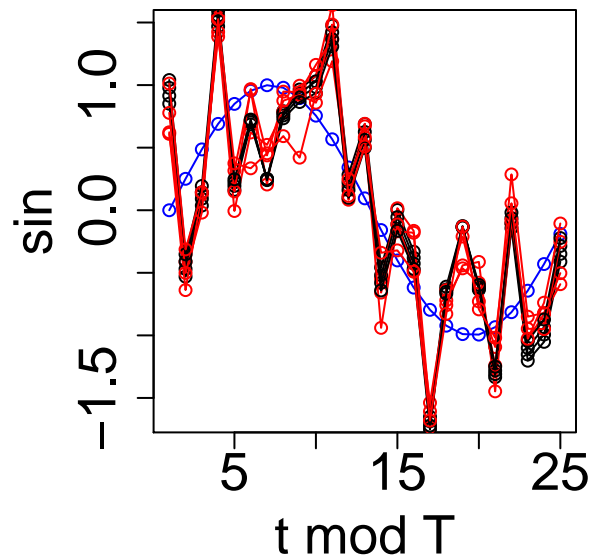

**(A):  $A = 2$** 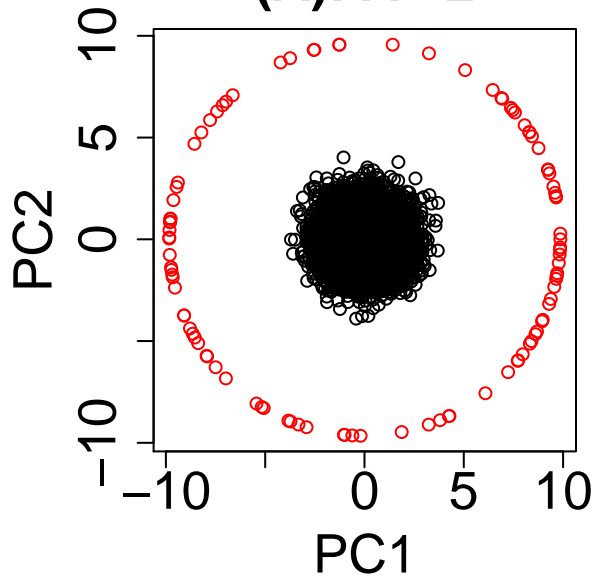**(B)**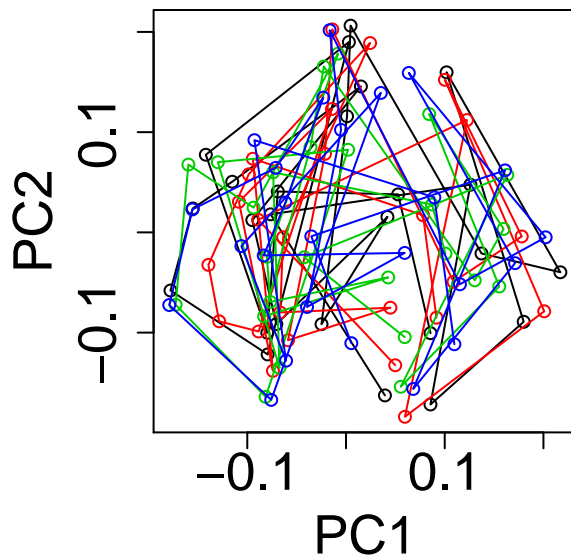**(C)**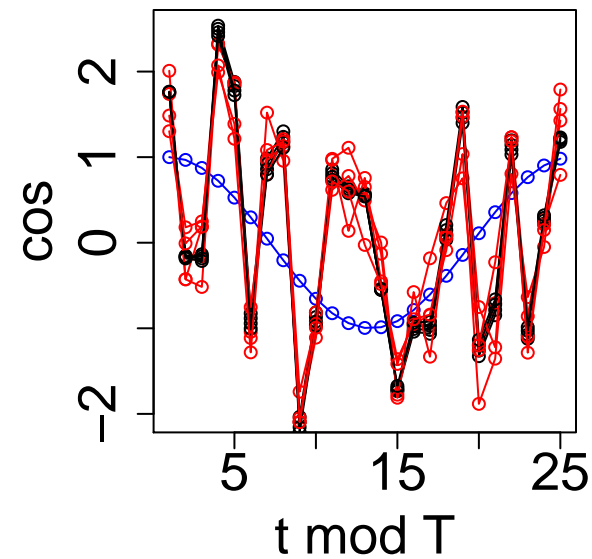**(D)**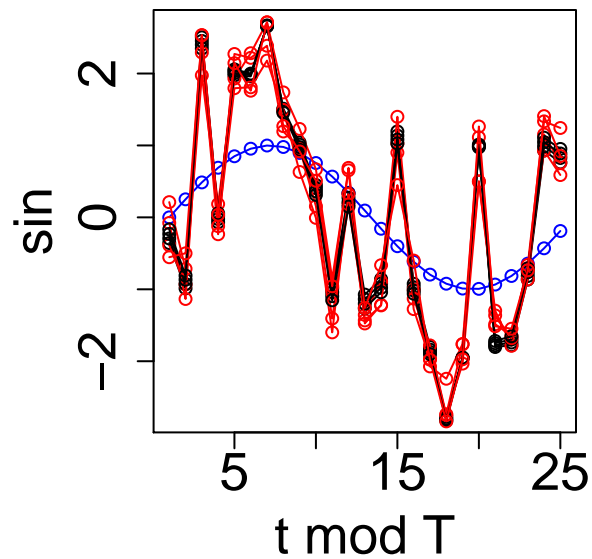

**(A):  $A = 3$** 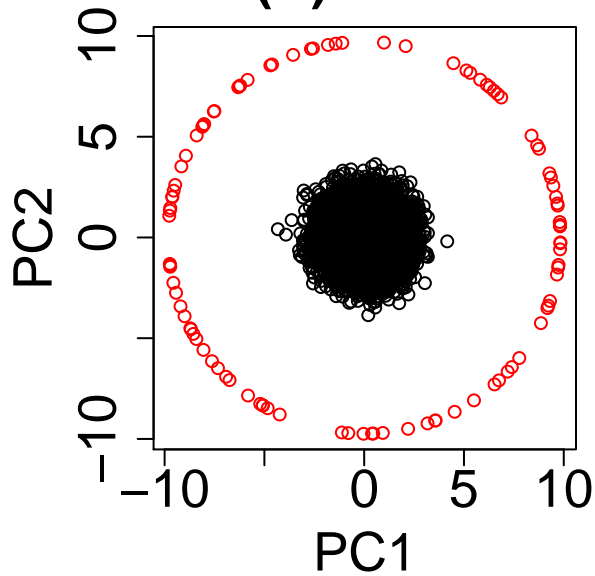**(B)**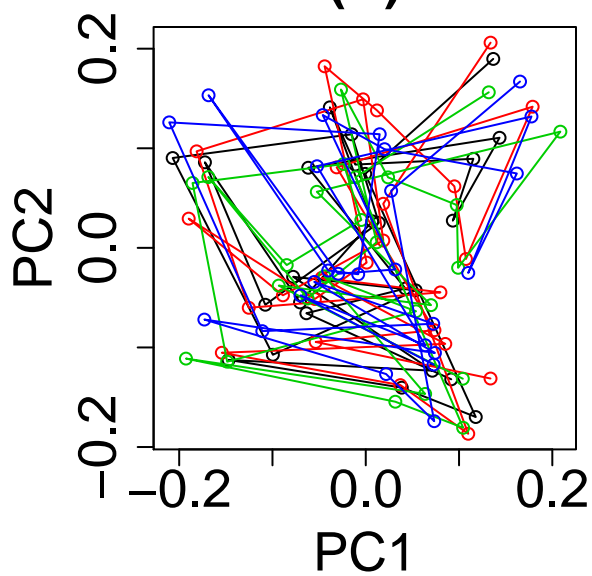**(C)**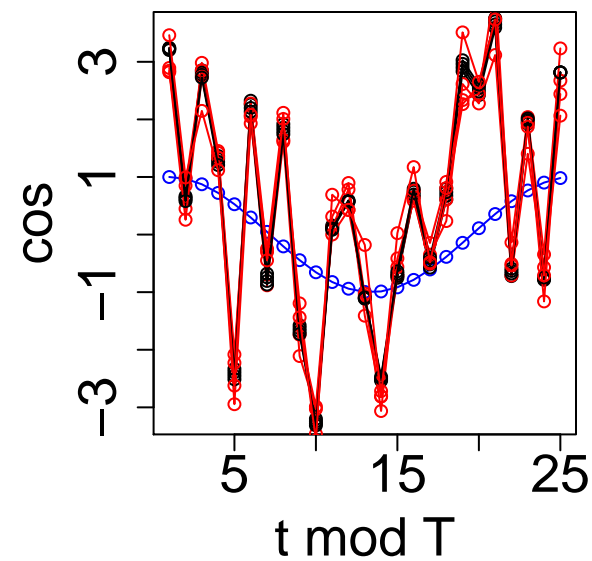**(D)**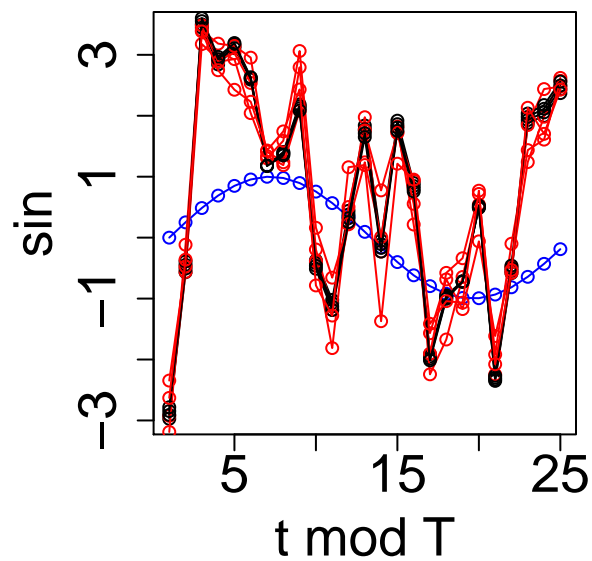

**(A):  $A = 4$**

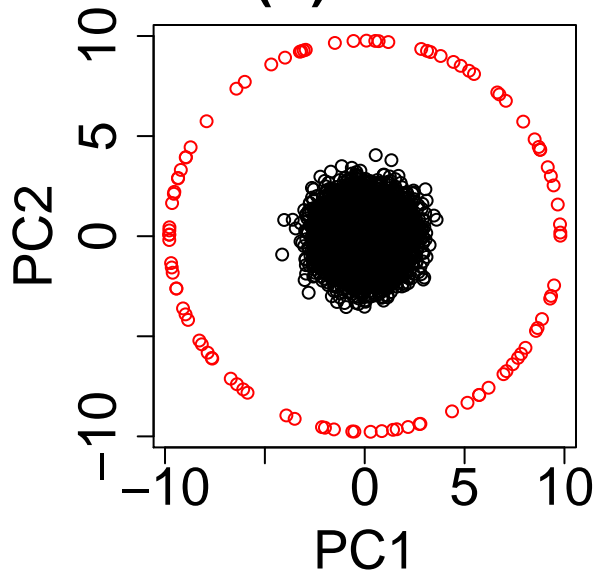

**(B)**

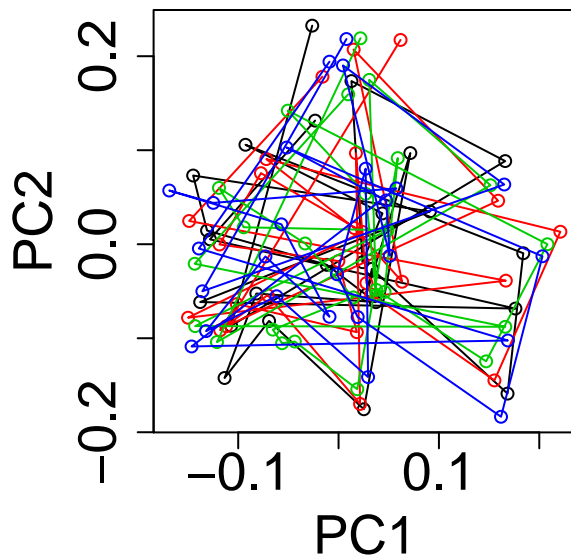

**(C)**

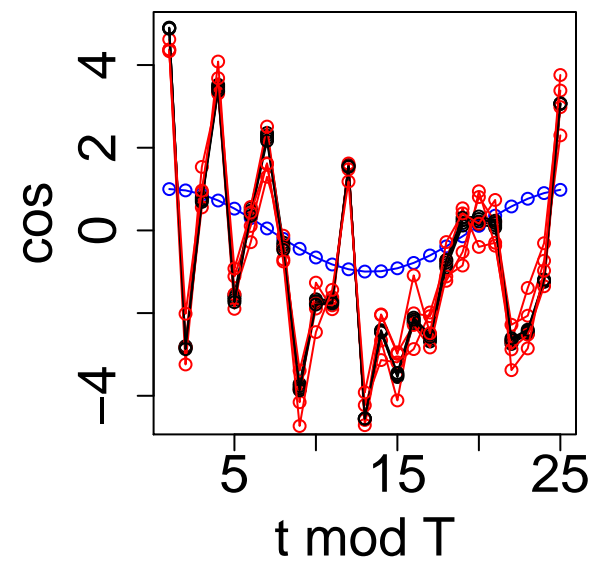

**(D)**

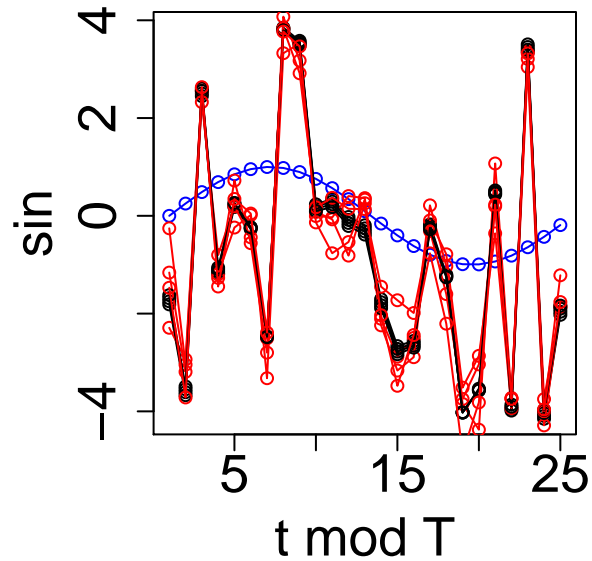

**(A):  $A = 5$** 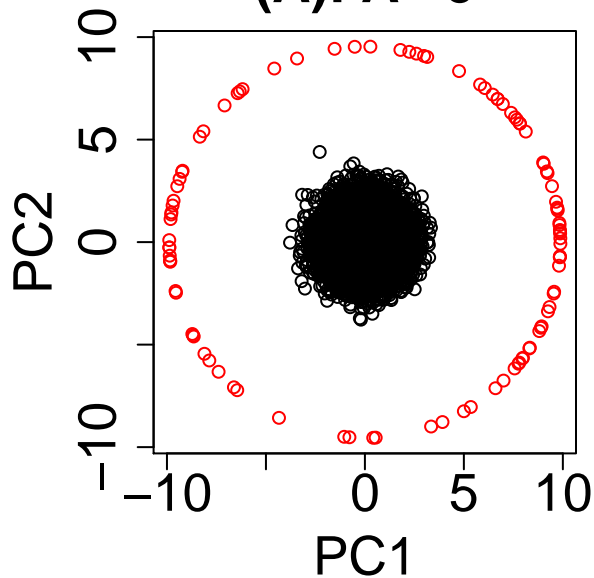**(B)**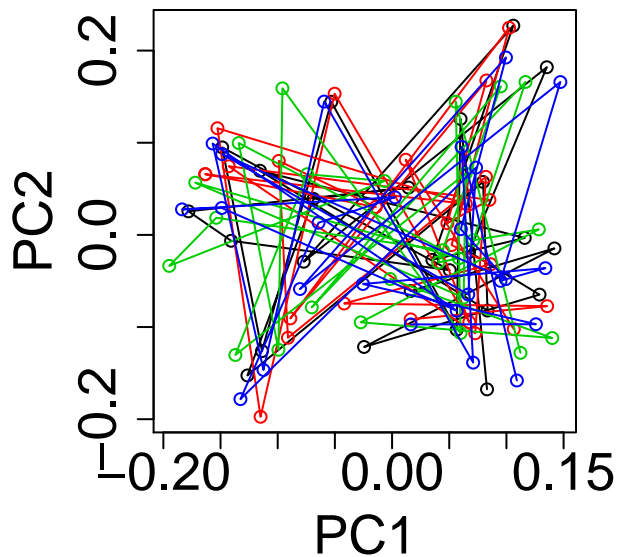**(C)**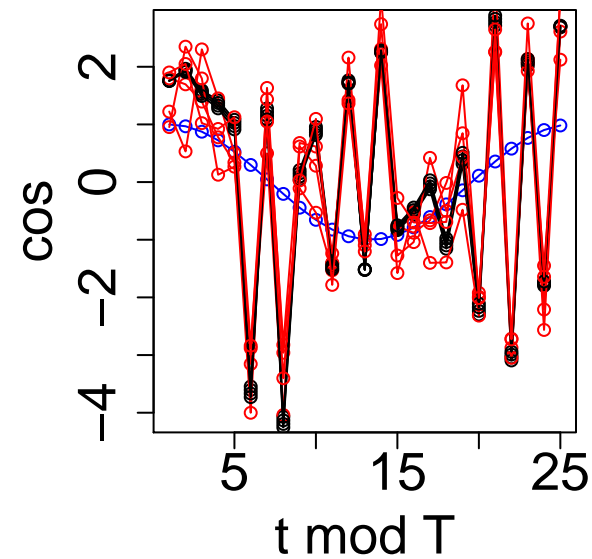**(D)**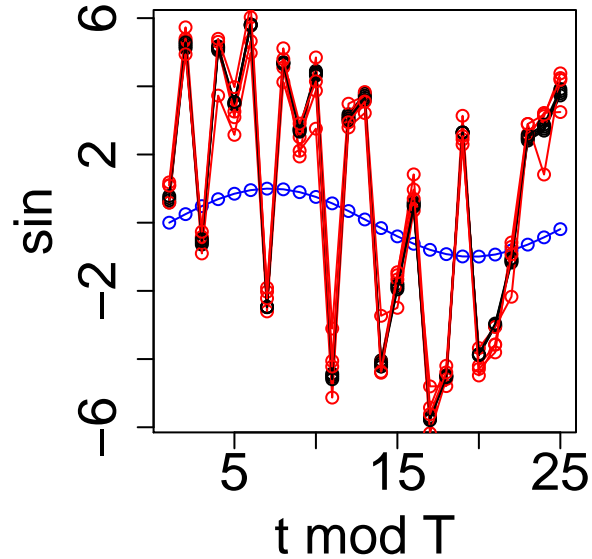

**(A):  $A = 6$** 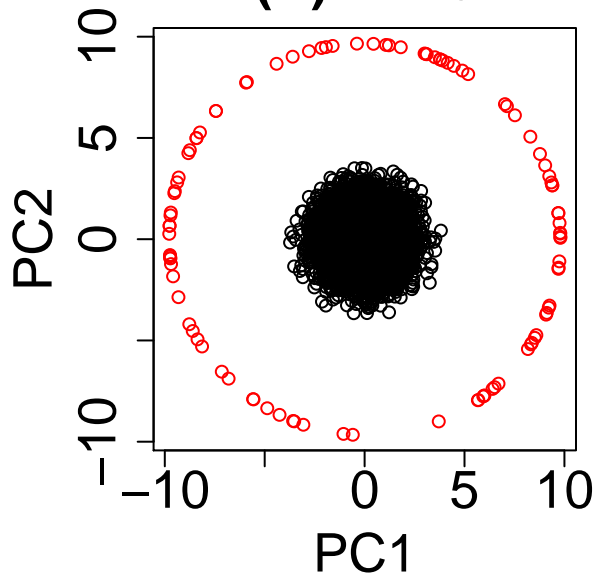**(B)**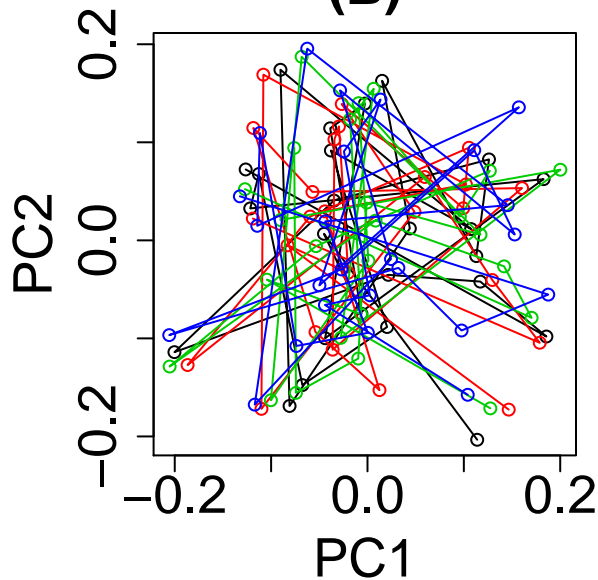**(C)**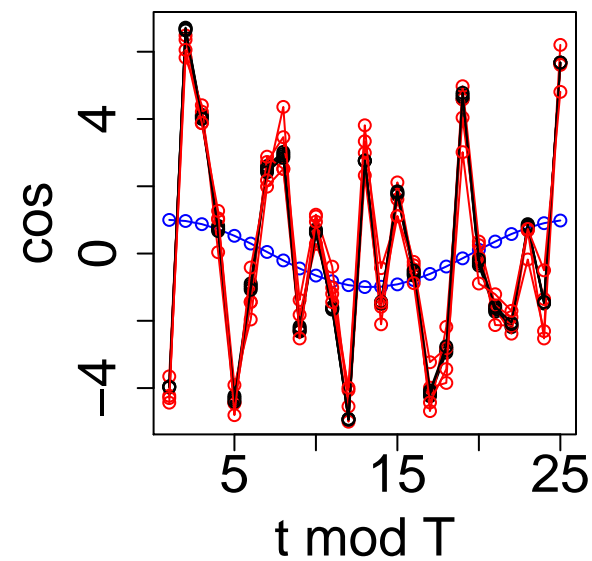**(D)**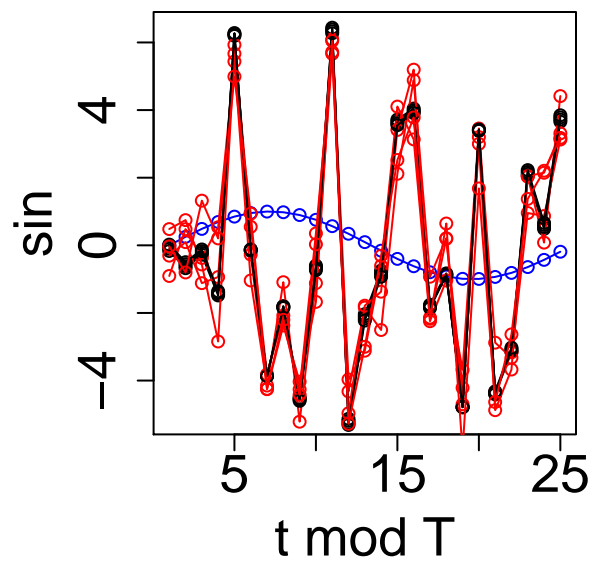

Supplement: Additional file 9 — Figure S2. PCA based unsupervised FE results for synthetic data (A=1,2,3,4,5,6). Representative examples (one of 100 ensembles, from which the averages shown in Table 2 were taken). (A) The first and second PCA scores attributed to genes. Red open circles correspond to genes associated with significant adjusted P-values (<0.01), which are equivalent to genes i≤102. (B) The first and second PC loading attributed to each sample. Black: 1≤j≤25, red: 26≤j≤50, green: 51≤j≤75, blue: 76≤j≤100. (C) Regression analysis, C j=av 1j+bv 2j. Black: C j, red: fitted results. Blue: ${C^{0}_{j}}$Cj0. (D) Regression analysis, S j=av 1j+bv 2j. Black: S j, red: fitted results. Blue: ${S^{0}_{j}}$Sj0. (PDF 402 kb) [file 13040_2016_101_MOESM9_ESM.pdf]

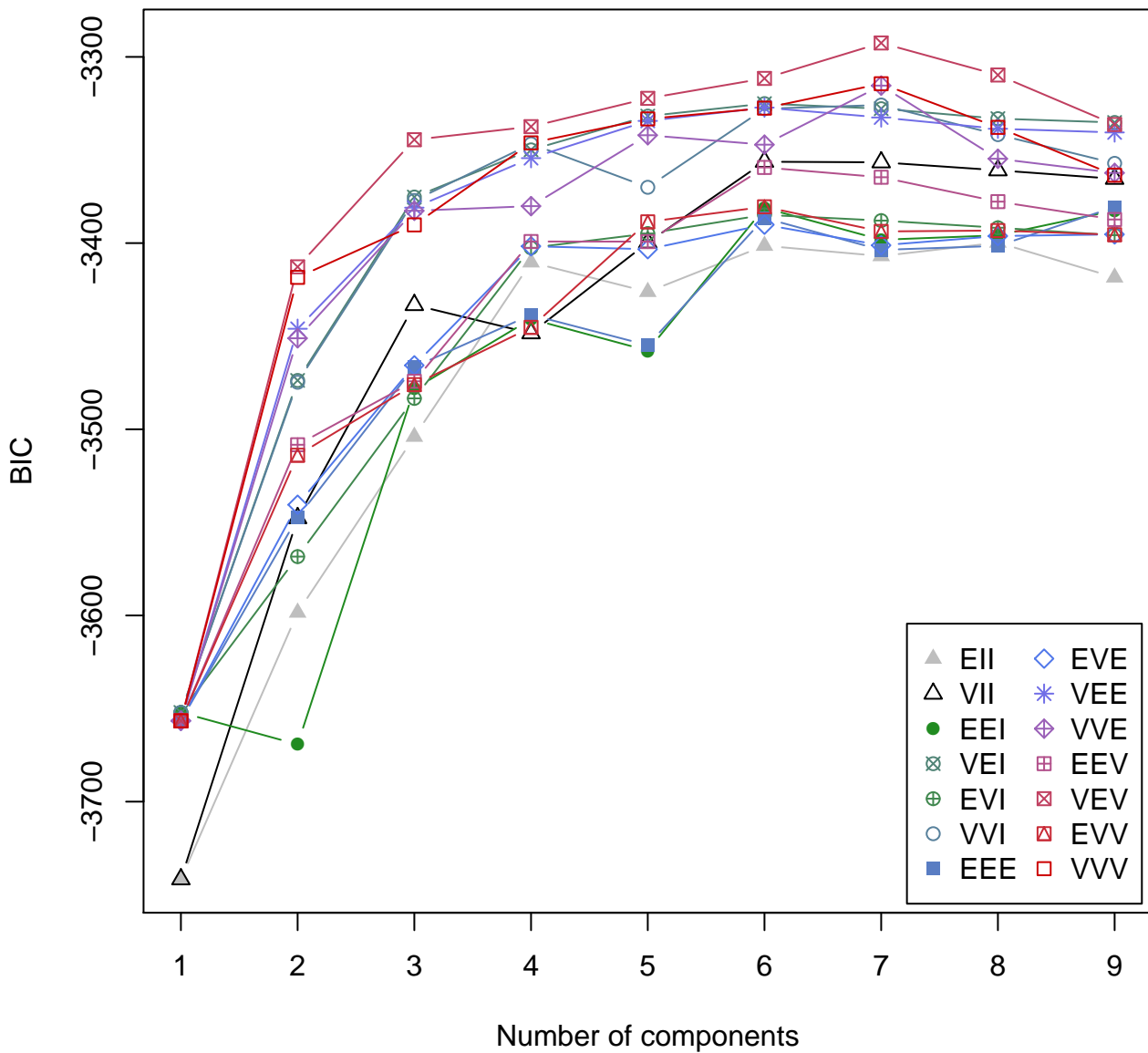

# Classification

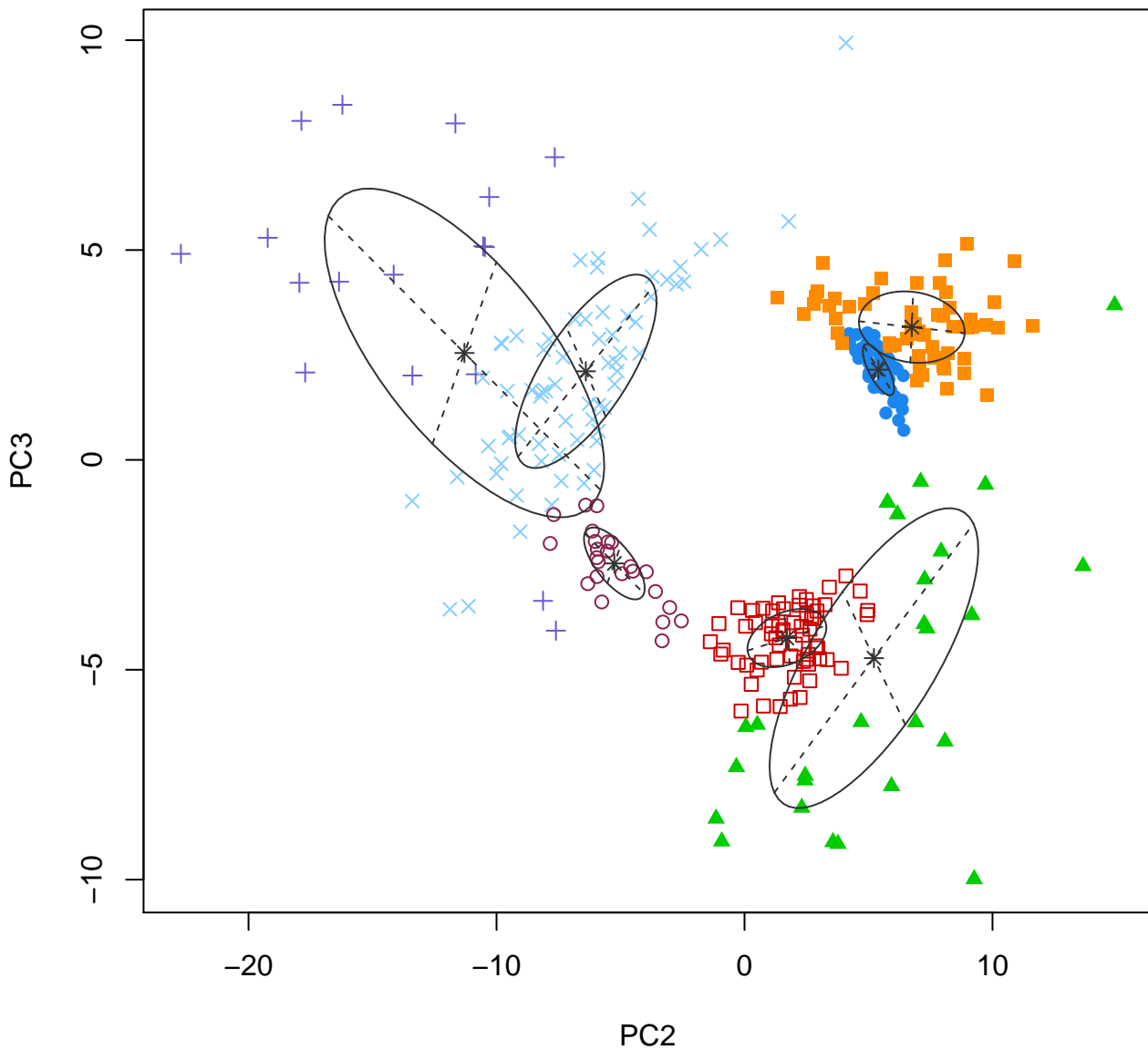

# Classification Uncertainty

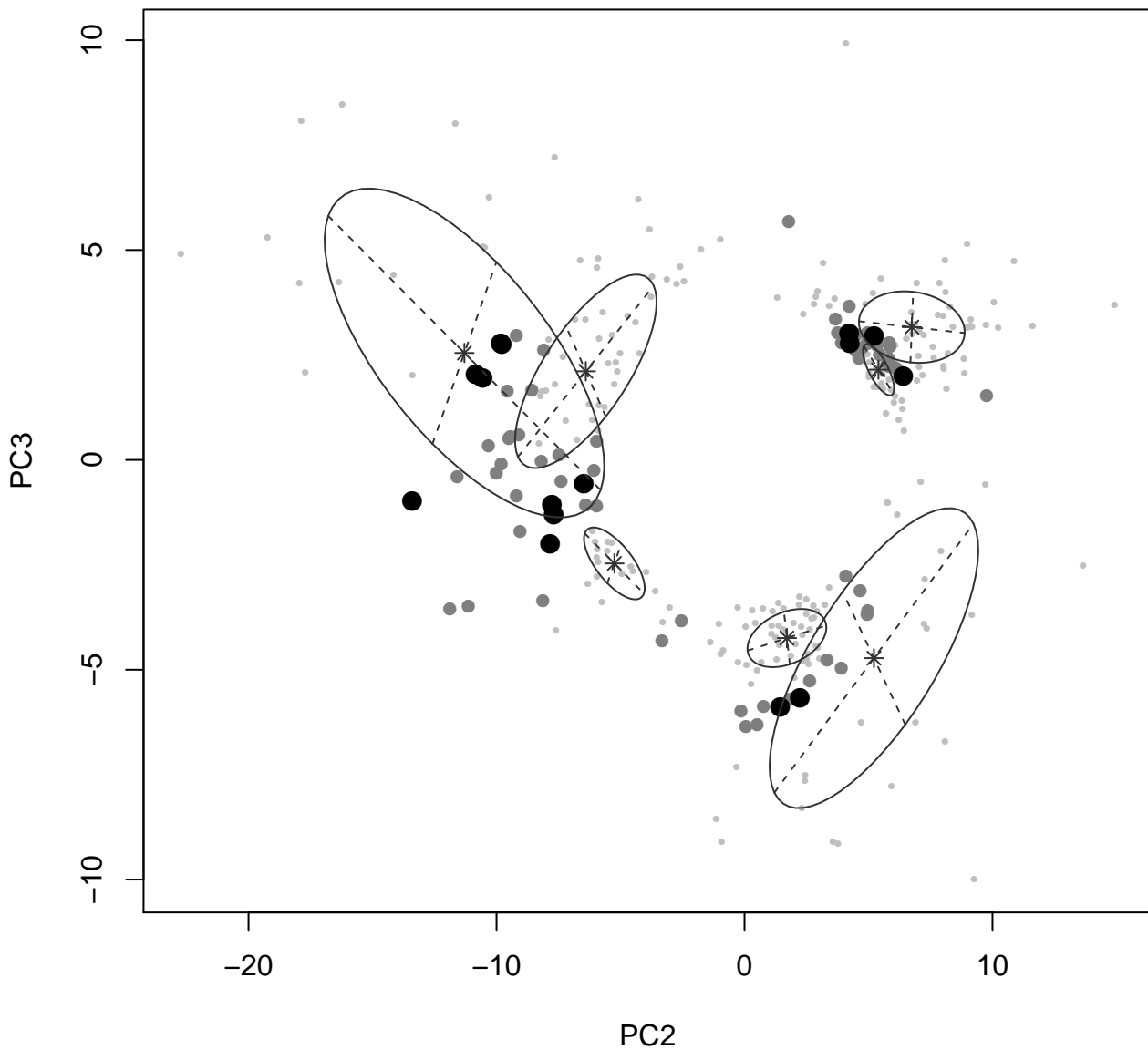

**log Density Contour Plot**

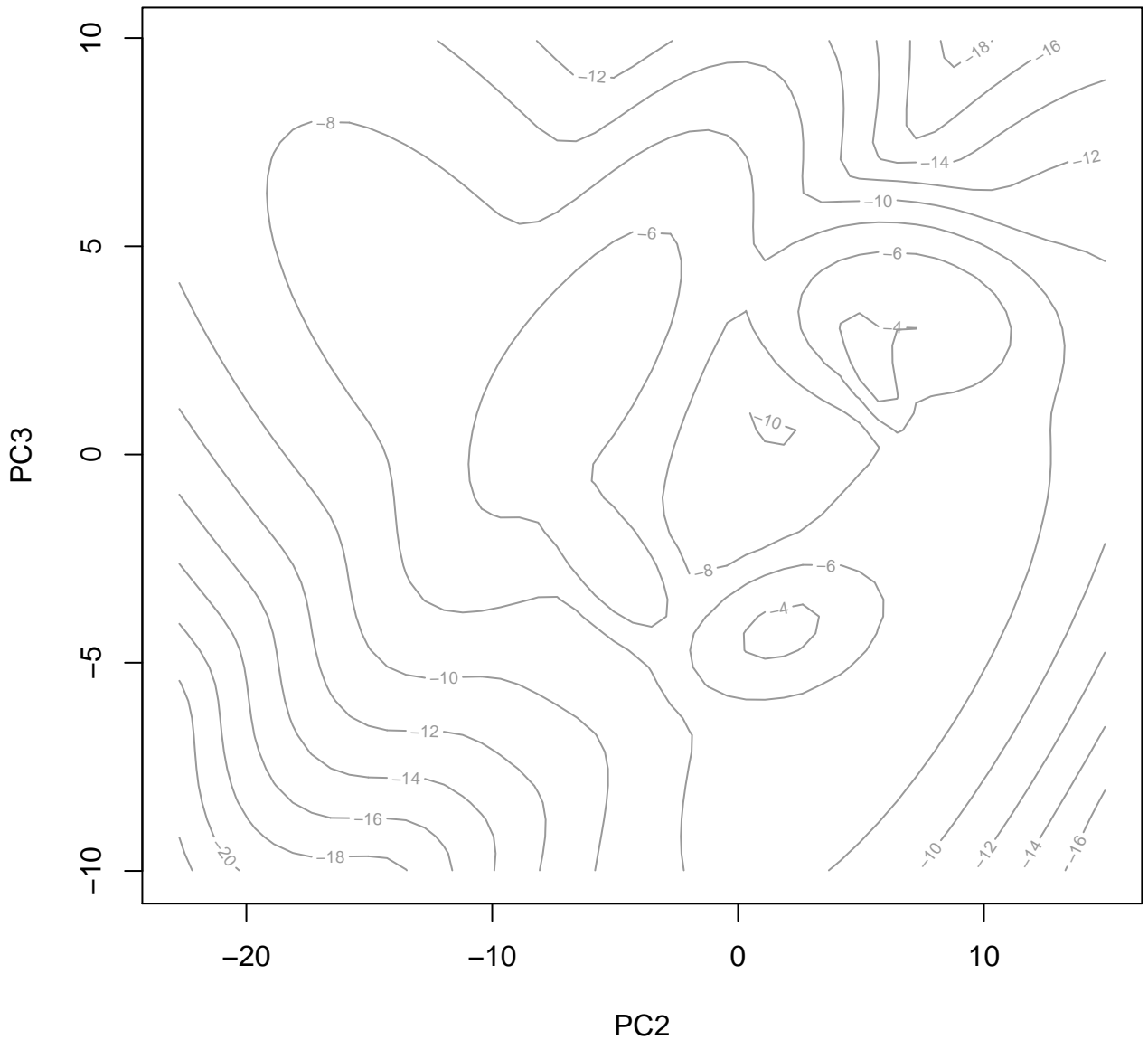

Supplement: Additional file 10 — Figure S3. Detailed results obtained from mclust (Gaussian mixture). (PDF 30.5 kb) [file 13040_2016_101_MOESM10_ESM.pdf]
