# Supplementary material for: Principal component analysis based unsupervised feature extraction applied to budding yeast temporally periodic gene expression
Source: BioData Min. 2016 Jun 29;9:22. doi: 10.1186/s13040-016-0101-9 (PMC4928327; doi:10.1186/s13040-016-0101-9)
Supplement: Additional file 11 — Table S7. Enrichment analyses by g:profiler for the YMC genes that correspond to Figure 9A to 9E. (PDF 229 kb) [file 13040_2016_101_MOESM11_ESM.pdf]

source	term name<br>Gene Ontology (Biological process)	term ID	n. of term genes	corrected p-value
BP	monosaccharide metabolic process	GO:0005996	91	1.12e-02
BP	hexose metabolic process	GO:0019318	85	4.05e-03
BP	glucose metabolic process	GO:0006006	68	1.15e-04
BP	monosaccharide biosynthetic process	GO:0046364	35	5.95e-03
BP	hexose biosynthetic process	GO:0019319	34	4.32e-03
BP	gluconeogenesis	GO:0006094	33	3.09e-03
BP	ribosomal small subunit assembly	GO:0000028	16	2.78e-02
BP	rRNA transport	GO:0051029	18	1.20e-06
BP	rRNA export from nucleus	GO:0006407	18	1.20e-06
BP	response to oxidative stress	GO:0006979	105	7.23e-03
BP	cellular response to oxidative stress	GO:0034599	97	3.03e-02
BP	lysine metabolic process	GO:0006553	14	9.35e-03
BP	lysine biosynthetic process	GO:0009085	13	4.94e-03
BP	lysine biosynthetic process via aminoadipic acid	GO:0019878	11	1.06e-03
BP	metabolic process	GO:0008152	4109	3.57e-06
BP	organic substance metabolic process	GO:0071704	3723	1.44e-07
BP	carbohydrate derivative metabolic process	GO:1901135	367	3.20e-12
BP	organonitrogen compound metabolic process	GO:1901564	651	1.67e-17
BP	organonitrogen compound catabolic process	GO:1901565	110	4.28e-02
BP	pyridine-containing compound metabolic process	GO:0072524	100	1.50e-10
BP	purine-containing compound metabolic process	GO:0072521	170	6.49e-24
BP	primary metabolic process	GO:0044238	3554	1.25e-05
BP	protein metabolic process	GO:0019538	1543	9.13e-04
BP	cellular protein metabolic process	GO:0044267	1430	2.76e-04
BP	protein folding	GO:0006457	110	9.41e-04
BP	'de novo' protein folding	GO:0006458	11	1.05e-03
BP	protein refolding	GO:0042026	17	2.33e-04
BP	biosynthetic process	GO:0009058	2100	5.08e-03
BP	organic substance biosynthetic process	GO:1901576	2070	1.83e-03
BP	organonitrogen compound biosynthetic process	GO:1901566	385	4.21e-04
BP	mitochondrial RNA metabolic process	GO:0000959	28	4.04e-03
BP	single-organism process	GO:0044699	3755	1.16e-17
BP	single-organism metabolic process	GO:0044710	2051	1.05e-17
BP	small molecule metabolic process	GO:0044281	789	3.94e-29
BP	single-organism biosynthetic process	GO:0044711	858	8.27e-22
BP	small molecule biosynthetic process	GO:0044283	315	2.80e-02
BP	single-organism catabolic process	GO:0044712	398	1.17e-03
BP	small molecule catabolic process	GO:0044282	113	1.52e-03
BP	glycosyl compound metabolic process	GO:1901657	165	1.25e-25
BP	glycosyl compound biosynthetic process	GO:1901659	65	3.16e-04
BP	oxidation-reduction process	GO:0055114	437	3.77e-19
BP	cellular process	GO:0009987	4884	2.36e-10
BP	single-organism cellular process	GO:0044763	3397	3.56e-21
BP	nucleobase-containing small molecule metabolic process	GO:0055086	279	1.66e-22
BP	nucleoside metabolic process	GO:0009116	161	2.80e-26
BP	ribonucleoside metabolic process	GO:0009119	154	1.80e-26
BP	purine nucleoside metabolic process	GO:0042278	140	6.08e-27
BP	purine ribonucleoside metabolic process	GO:0046128	140	6.08e-27
BP	nucleoside biosynthetic process	GO:0009163	61	1.15e-04
BP	ribonucleoside biosynthetic process	GO:0042455	60	8.77e-05
BP	cellular metabolic process	GO:0044237	3696	1.78e-09
BP	generation of precursor metabolites and energy	GO:0006091	186	1.89e-23
BP	energy derivation by oxidation of organic compounds	GO:0015980	152	6.26e-15
BP	cellular respiration	GO:0045333	103	1.69e-15
BP	aerobic respiration	GO:0009060	78	5.85e-12
BP	electron transport chain	GO:0022900	32	3.47e-11
BP	respiratory electron transport chain	GO:0022904	31	1.61e-11
BP	phosphorus metabolic process	GO:0006793	760	1.03e-06
BP	organophosphate metabolic process	GO:0019637	417	8.59e-14
BP	phosphate-containing compound metabolic process	GO:0006796	737	6.36e-05
BP	nucleoside phosphate metabolic process	GO:0006753	247	1.50e-23
BP	nucleoside monophosphate metabolic process	GO:0009123	135	8.78e-25
BP	ribonucleoside monophosphate metabolic process	GO:0009161	132	2.47e-25
BP	purine nucleoside monophosphate metabolic process	GO:0009126	116	1.69e-27
BP	purine ribonucleoside monophosphate metabolic process	GO:0009167	116	1.69e-27
BP	nucleoside phosphate biosynthetic process	GO:1901293	121	1.75e-02
BP	nucleoside monophosphate biosynthetic process	GO:0009124	61	1.88e-02
BP	purine nucleoside monophosphate biosynthetic process	GO:0009127	42	9.97e-04
BP	ribonucleoside monophosphate biosynthetic process	GO:0009156	58	1.00e-02
BP	purine ribonucleoside monophosphate biosynthetic process	GO:0009168	42	9.97e-04
BP	nucleotide metabolic process	GO:0009117	243	5.19e-24
BP	purine nucleotide metabolic process	GO:0006163	139	4.45e-25
BP	nucleotide biosynthetic process	GO:0009165	119	4.53e-02
BP	nucleoside triphosphate metabolic process	GO:0009141	70	1.62e-15
BP	ribonucleoside triphosphate metabolic process	GO:0009199	62	2.09e-17
BP	purine nucleoside triphosphate metabolic process	GO:0009144	60	9.66e-17
BP	purine ribonucleoside triphosphate metabolic process	GO:0009205	58	2.85e-17
BP	nucleoside triphosphate biosynthetic process	GO:0009142	29	6.95e-04
BP	ribonucleoside triphosphate biosynthetic process	GO:0009201	26	1.84e-04
BP	purine nucleoside triphosphate biosynthetic process	GO:0009145	22	3.01e-04
BP	purine ribonucleoside triphosphate biosynthetic process	GO:0009206	22	3.01e-04
BP	ribose phosphate metabolic process	GO:0019693	168	4.08e-26
BP	ribonucleotide metabolic process	GO:0009259	148	1.65e-25
BP	purine ribonucleotide metabolic process	GO:0009150	135	8.30e-26
BP	ATP metabolic process	GO:0046034	54	3.63e-17
BP	oxidative phosphorylation	GO:0006119	32	1.62e-12
BP	ATP synthesis coupled electron transport	GO:0042773	29	3.03e-12
BP	mitochondrial ATP synthesis coupled electron tran ...	GO:0042775	29	3.03e-12
BP	mitochondrial electron transport, cytochrome c ...	GO:0006123	12	2.40e-03
BP	mitochondrial electron transport, ubiquinol to ...	GO:0006122	11	1.05e-03
BP	thioester metabolic process	GO:0035383	20	1.58e-02
BP	organic acid metabolic process	GO:0006082	434	2.45e-23
BP	oxoacid metabolic process	GO:0043436	433	2.04e-23
BP	carboxylic acid metabolic process	GO:0019752	417	5.31e-24
BP	tricarboxylic acid metabolic process	GO:0072350	29	1.47e-09
BP	citrate metabolic process	GO:0006101	29	1.47e-09
BP	tricarboxylic acid cycle	GO:0006099	29	1.47e-09
BP	dicarboxylic acid metabolic process	GO:0043648	44	1.15e-02
BP	monocarboxylic acid metabolic process	GO:0032787	157	1.56e-18
BP	pyruvate metabolic process	GO:0006090	46	7.32e-10
BP	cellular amino acid metabolic process	GO:0006520	262	5.67e-03
BP	alpha-amino acid metabolic process	GO:1901605	195	3.63e-03
BP	glutamine family amino acid metabolic process	GO:0009064	61	1.88e-02
BP	organic acid catabolic process	GO:0016054	89	1.90e-03
BP	carboxylic acid catabolic process	GO:0046395	89	1.90e-03
BP	monocarboxylic acid catabolic process	GO:0072329	30	1.04e-03
BP	cofactor metabolic process	GO:0051186	206	8.77e-10
BP	coenzyme metabolic process	GO:0006732	182	2.14e-10
BP	oxidoreduction coenzyme metabolic process	GO:0006733	99	5.08e-09
BP	pyridine nucleotide metabolic process	GO:0019362	85	7.27e-11
BP	nicotinamide nucleotide metabolic process	GO:0046496	84	4.08e-10
BP	NADP metabolic process	GO:0006739	22	3.39e-02
BP	acyl-CoA metabolic process	GO:0006637	20	1.58e-02
BP	cofactor catabolic process	GO:0051187	9	4.61e-03
BP	coenzyme catabolic process	GO:0009109	8	4.99e-02
BP	cellular biosynthetic process	GO:0044249	2030	5.91e-03
BP	organic acid biosynthetic process	GO:0016053	191	6.95e-03
BP	carboxylic acid biosynthetic process	GO:0046394	191	6.95e-03
BP	cellular amino acid biosynthetic process	GO:0008652	144	2.83e-02
BP	alpha-amino acid biosynthetic process	GO:1901607	136	3.47e-02
BP	glutamine family amino acid biosynthetic process	GO:0009084	29	6.95e-04
BP	glutamate biosynthetic process	GO:0006537	11	2.28e-02
BP	cellular carbohydrate metabolic process	GO:0044262	176	4.00e-03
BP	cellular aldehyde metabolic process	GO:0006081	53	1.42e-07
BP	aldehyde catabolic process	GO:0046185	8	4.80e-02
BP	protein localization to mitochondrion	GO:0070585	66	4.02e-04
BP	establishment of protein localization to mitochondrion	GO:0072655	66	4.02e-04
BP	cellular component organization or biogenesis	GO:0071840	2397	1.82e-02
BP	cellular component organization	GO:0016043	2045	8.00e-08
BP	organelle organization	GO:0006996	1491	7.10e-12
BP	single-organism organelle organization	GO:1902589	1007	2.20e-13
BP	mitochondrion organization	GO:0007005	377	2.13e-48
BP	mitochondrial membrane organization	GO:0007006	49	1.80e-04
BP	inner mitochondrial membrane organization	GO:0007007	27	2.40e-02
BP	cristae formation	GO:0042407	10	1.09e-02
BP	protein complex biogenesis	GO:0070271	350	5.45e-03
BP	mitochondrial respiratory chain complex IV biogenesis	GO:0097034	27	2.96e-03
BP	ribose phosphate biosynthetic process	GO:0046390	76	1.53e-02
BP	ribonucleotide biosynthetic process	GO:0009260	70	4.95e-03
BP	purine-containing compound biosynthetic process	GO:0072522	70	9.98e-04
BP	purine nucleoside biosynthetic process	GO:0042451	48	1.23e-04
BP	purine ribonucleoside biosynthetic process	GO:0046129	48	1.23e-04
BP	purine nucleotide biosynthetic process	GO:0006164	60	1.53e-02
BP	purine ribonucleotide biosynthetic process	GO:0009152	57	8.07e-03
BP	ATP biosynthetic process	GO:0006754	19	7.91e-04
BP	carbohydrate catabolic process	GO:0016052	95	1.22e-05
BP	single-organism carbohydrate catabolic process	GO:0044724	82	9.90e-05
BP	glycolytic process	GO:0006096	32	2.10e-06
BP	hydrogen transport	GO:0006818	68	1.15e-04
BP	ion transport	GO:0006811	319	1.56e-04
BP	cation transport	GO:0006812	176	1.21e-02
BP	monovalent inorganic cation transport	GO:0015672	98	2.30e-05
BP	proton transport	GO:0015992	67	8.95e-05
BP	protein complex assembly	GO:0006461	339	3.01e-02
BP	cellular protein complex assembly	GO:0043623	236	3.63e-03
BP	cytochrome complex assembly	GO:0017004	32	1.70e-02
BP	mitochondrial respiratory chain complex assembly	GO:0033108	36	1.08e-03
BP	transmembrane transport	GO:0055085	407	1.64e-04
BP	ion transmembrane transport	GO:0034220	146	2.82e-04
BP	cation transmembrane transport	GO:0098655	94	4.52e-03
BP	protein transmembrane transport	GO:0071806	83	6.09e-04
BP	intracellular protein transmembrane transport	GO:0065002	83	6.09e-04
BP	intracellular protein transmembrane import	GO:0044743	70	4.95e-03
BP	inorganic ion transmembrane transport	GO:0098660	101	8.56e-04
BP	inorganic cation transmembrane transport	GO:0098662	88	1.59e-03
BP	hydrogen ion transmembrane transport	GO:1902600	57	5.31e-06
BP	energy coupled proton transport, down electrochemical gradient	GO:0015985	17	4.47e-02
BP	ATP synthesis coupled proton transport	GO:0015986	17	4.47e-02
BP	mitochondrial transport	GO:0006839	80	1.59e-03
BP	mitochondrial transmembrane transport	GO:1990542	48	5.34e-03
BP	protein targeting to mitochondrion	GO:0006626	59	4.15e-04
BP	protein import into mitochondrial matrix	GO:0030150	20	1.49e-02
BP	fatty acid metabolic process	GO:0006631	65	8.71e-03
BP	fatty acid catabolic process	GO:0009062	18	6.14e-03
BP	translation	GO:0006412	476	1.39e-20
BP	cytoplasmic translation	GO:0002181	171	1.96e-42
BP	mitochondrial translation	GO:0032543	126	6.22e-52
BP	tRNA aminoacylation for mitochondrial protein translation	GO:0070127	13	2.24e-04
BP	translational elongation	GO:0006414	100	1.17e-02
BP	NADH metabolic process	GO:0006734	15	1.65e-02
BP	NADH oxidation	GO:0006116	10	1.09e-02

source	term name<br>Gene Ontology (Cellular component)	term ID	n. of term genes	corrected p-value
CC	cell	GO:0005623	5339	5.72e-11
CC	cell part	GO:0044464	5339	5.72e-11
CC	cell periphery	GO:0071944	661	3.34e-07
CC	envelope	GO:0031975	552	2.10e-05
CC	external encapsulating structure	GO:0030312	106	1.10e-04
CC	cell wall	GO:0005618	106	1.10e-04
CC	fungal-type cell wall	GO:0009277	99	1.34e-04
CC	intracellular	GO:0005622	5073	7.15e-04
CC	intracellular part	GO:0044424	5062	2.39e-03
CC	cytoplasm	GO:0005737	4072	1.01e-16
CC	cytoplasmic part	GO:0044444	2797	8.11e-25
CC	microbody	GO:0042579	70	1.86e-04
CC	peroxisome	GO:0005777	70	1.86e-04
CC	cytosol	GO:0005829	573	3.23e-29
CC	cytosolic part	GO:0044445	220	2.23e-40
CC	mitochondrion	GO:0005739	1124	4.59e-42
CC	mitochondrial part	GO:0044429	603	1.66e-42
CC	ribonucleoprotein complex	GO:0030529	685	7.14e-12
CC	plasma membrane	GO:0005886	469	3.51e-07
CC	oxidoreductase complex	GO:1990204	36	1.26e-06
CC	membrane-enclosed lumen	GO:0031974	1132	1.44e-03
CC	cytochrome complex	GO:0070069	22	6.17e-08
CC	non-membrane-bounded organelle	GO:0043228	1276	4.16e-02
CC	intracellular non-membrane-bounded organelle	GO:0043232	1276	4.16e-02
CC	ribosome	GO:0005840	264	7.14e-31
CC	ribosomal subunit	GO:0044391	230	4.98e-30
CC	large ribosomal subunit	GO:0015934	131	1.25e-18
CC	small ribosomal subunit	GO:0015935	99	5.08e-09
CC	cytosolic ribosome	GO:0022626	161	5.85e-46
CC	cytosolic large ribosomal subunit	GO:0022625	86	1.17e-26
CC	cytosolic small ribosomal subunit	GO:0022627	64	1.21e-14
CC	organellar ribosome	GO:0000313	83	4.68e-47
CC	organellar large ribosomal subunit	GO:0000315	44	1.09e-31
CC	organellar small ribosomal subunit	GO:0000314	34	6.66e-12
CC	proton-transporting ATP synthase complex	GO:0045259	17	4.47e-02
CC	organelle lumen	GO:0043233	1074	7.46e-03
CC	intracellular organelle lumen	GO:0070013	1074	7.46e-03
CC	mitochondrial matrix	GO:0005759	216	1.68e-43
CC	mitochondrial ribosome	GO:0005761	83	4.68e-47
CC	mitochondrial large ribosomal subunit	GO:0005762	44	1.09e-31
CC	mitochondrial small ribosomal subunit	GO:0005763	34	6.66e-12
CC	transmembrane transporter complex	GO:1902495	11	2.28e-02
CC	organelle envelope	GO:0031967	552	2.10e-05
CC	organelle inner membrane	GO:0019866	254	1.25e-05
CC	organelle envelope lumen	GO:0031970	66	1.06e-02
CC	mitochondrial envelope	GO:0005740	418	5.97e-09
CC	mitochondrial intermembrane space	GO:0005758	61	3.74e-03
CC	respiratory chain	GO:0070469	32	1.62e-12
CC	respiratory chain complex IV	GO:0045277	12	2.40e-03
CC	respiratory chain complex III	GO:0045275	10	1.09e-02
CC	mitochondrial membrane	GO:0031966	379	2.30e-08
CC	mitochondrial membrane part	GO:0044455	168	2.51e-06
CC	intrinsic component of mitochondrial membrane	GO:0098573	62	2.37e-05
CC	integral component of mitochondrial membrane	GO:0032592	59	6.67e-05
CC	mitochondrial inner membrane	GO:0005743	242	2.88e-06
CC	mitochondrial respiratory chain	GO:0005746	27	1.31e-11
CC	mitochondrial respiratory chain complex IV	GO:0005751	12	2.40e-03
CC	mitochondrial respiratory chain complex III	GO:0005750	10	1.09e-02
CC	mitochondrial proton-transporting ATP synthase complex	GO:0005753	17	4.47e-02
CC	mitochondrial outer membrane translocase complex	GO:0005742	7	4.29e-04
CC	extracellular region	GO:0005576	97	1.87e-05

source	term name<br>Gene Ontology (Molecular function)	term ID	n. of term genes	corrected p-value
MF	structural molecule activity	GO:0005198	359	1.76e-22
MF	structural constituent of ribosome	GO:0003735	225	8.19e-30
MF	cofactor binding	GO:0048037	153	4.18e-08
MF	coenzyme binding	GO:0050662	103	4.86e-07
MF	NAD binding	GO:0051287	29	6.69e-05
MF	catalytic activity	GO:0003824	2171	2.07e-03
MF	oxidoreductase activity	GO:0016491	331	1.49e-11
MF	oxidoreductase activity, acting on CH-OH group of donors	GO:0016614	85	9.02e-04
MF	oxidoreductase activity, acting on the CH-OH group of donors, NAD ...	GO:0016616	82	2.34e-03
MF	oxidoreductase activity, acting on the aldehyde or oxo group of donors	GO:0016903	28	3.56e-02
MF	oxidoreductase activity, acting on the aldehyde or oxo group of do ...	GO:0016620	22	3.59e-02
MF	lyase activity	GO:0016829	108	3.67e-02
MF	antioxidant activity	GO:0016209	32	1.75e-02
MF	peroxidase activity	GO:0004601	17	4.48e-02
MF	transferase activity, transferring acyl groups, acyl groups converted in ...	GO:0046912	11	1.05e-03
MF	electron carrier activity	GO:0009055	47	2.40e-02
MF	transporter activity	GO:0005215	414	8.51e-03
MF	substrate-specific transporter activity	GO:0022892	351	2.46e-03
MF	transmembrane transporter activity	GO:0022857	329	5.37e-05
MF	substrate-specific transmembrane transporter activity	GO:0022891	294	1.07e-04
MF	ion transmembrane transporter activity	GO:0015075	219	2.05e-04
MF	cation transmembrane transporter activity	GO:0008324	151	1.98e-03
MF	inorganic cation transmembrane transporter activity	GO:0022890	124	2.58e-02
MF	monovalent inorganic cation transmembrane transporter activity	GO:0015077	85	3.67e-05
MF	hydrogen ion transmembrane transporter activity	GO:0015078	60	8.77e-05
MF	unfolded protein binding	GO:0051082	78	1.07e-03

source	term name<br>Biological pathways (KEGG)	term ID	n. of term genes	corrected p-value
ke	Propanoate metabolism	KEGG:00640	11	2.46e-02
ke	Peroxisome	KEGG:04146	38	6.61e-04
ke	Metabolic pathways	KEGG:01100	687	3.18e-03
ke	Biosynthesis of secondary metabolites	KEGG:01110	275	1.98e-05
ke	Aminoacyl-tRNA biosynthesis	KEGG:00970	39	2.21e-02
ke	2-Oxocarboxylic acid metabolism	KEGG:01210	35	8.12e-04
ke	Glycolysis / Gluconeogenesis	KEGG:00010	58	4.16e-05
ke	Biosynthesis of antibiotics	KEGG:01130	217	2.82e-08
ke	Ribosome	KEGG:03010	181	5.10e-21
ke	Carbon metabolism	KEGG:01200	111	1.72e-11
ke	Citrate cycle (TCA cycle)	KEGG:00020	32	4.29e-06
ke	Pyruvate metabolism	KEGG:00620	39	1.21e-04
ke	Lysine biosynthesis	KEGG:00300	12	3.37e-04
ke	Biosynthesis of amino acids	KEGG:01230	123	1.07e-04
ke	Fatty acid degradation	KEGG:00071	19	8.34e-03
ke	Phenylalanine metabolism	KEGG:00360	11	3.83e-02
ke	Oxidative phosphorylation	KEGG:00190	72	4.70e-07
ke	Starch and sucrose metabolism	KEGG:00500	41	5.53e-04
ke	Glyoxylate and dicarboxylate metabolism	KEGG:00630	22	5.13e-05

source	term name<br>Biological pathways (Reactome)	term ID	n. of term genes	corrected p-value
re	Glycolysis	REAC:5719448	26	3.08e-03
re	Metabolism of proteins	REAC:5719483	308	2.97e-11
re	Mitochondrial protein import	REAC:5719964	59	4.30e-05
re	Gene Expression	REAC:5719443	319	8.37e-05
re	Nonsense-Mediated Decay (NMD)	REAC:5719944	145	2.79e-21
re	Nonsense Mediated Decay (NMD) enhanced by the Exon Junction Complex ...	REAC:5719945	145	2.79e-21
re	Nonsense Mediated Decay (NMD) independent of the Exon Junction Compl ...	REAC:5719943	143	9.74e-22
re	Translation	REAC:5719482	164	2.04e-17
re	SRP-dependent cotranslational protein targeting to membrane	REAC:5719995	138	6.25e-23
re	Eukaryotic Translation Elongation	REAC:5719700	139	1.10e-22
re	Peptide chain elongation	REAC:5719699	139	1.10e-22
re	Eukaryotic Translation Initiation	REAC:5719481	155	3.81e-19
re	L13a-mediated translational silencing of Ceruloplasmin expression	REAC:5719697	148	1.29e-20
re	Cap-dependent Translation Initiation	REAC:5719480	154	2.39e-19
re	GTP hydrolysis and joining of the 60S ribosomal subunit	REAC:5719486	136	1.98e-23
re	Formation of the ternary complex, and subsequently, the 43S complex	REAC:5719484	66	7.55e-05
re	Formation of a pool of free 40S subunits	REAC:5719487	136	1.98e-23
re	Ribosomal scanning and start codon recognition	REAC:5719479	70	2.92e-04
re	Gluconeogenesis	REAC:5719427	27	2.74e-02
re	Peroxisomal lipid metabolism	REAC:5719850	11	4.38e-03
re	Beta-oxidation of very long chain fatty acids	REAC:5719863	6	4.05e-02
re	Metabolism	REAC:5719430	431	2.66e-02
re	The citric acid (TCA) cycle and respiratory electron transport	REAC:5719463	36	1.06e-02
re	Pyruvate metabolism and Citric Acid (TCA) cycle	REAC:5719462	33	3.02e-03
re	Citric acid cycle (TCA cycle)	REAC:5719461	24	1.25e-04

source	term name<br>Regulatory motifs in DNA (TRANSFAC TFBS)	term ID	n. of term genes	corrected p-value
tf	Factor: HSF; motif: AGAANNTTCTAGAAN; match class: 3	TF:M00169_3	264	2.62e-04
tf	Factor: HSF; motif: AGAANNTTCTAGAAN; match class: 1	TF:M00169_1	113	6.63e-06
tf	Factor: HSF; motif: AGAANNTTCTAGAAN; match class: 0	TF:M00169_0	42	1.53e-04
tf	Factor: HSF; motif: AGAANAGAANNTTCT; match class: 4	TF:M00168_4	355	1.44e-02
tf	Factor: MCM1+SFF; motif: TTWCCYAAWNNGGAAAWWNGTAAAYAA; match class: 4	TF:M01051_4	26	3.07e-04
tf	Factor: MCM1+SFF; motif: TTWCCYAAWNNGGAAAWWNGTAAAYAA; match class: 3	TF:M01051_3	19	1.83e-02
tf	Factor: MIG1; motif: KANWWWWATSYGGGGWA; match class: 4	TF:M00061_4	13	1.74e-02
tf	Factor: TBP; motif: YNTTTATAT; match class: 4	TF:M00713_4	2164	1.95e-03
tf	Factor: TBP; motif: YNTTTATAT; match class: 3	TF:M00713_3	2070	3.16e-03
tf	Factor: TBP; motif: YNTTTATAT; match class: 2	TF:M00713_2	1710	1.73e-02
tf	Factor: RAP1; motif: WRMACCCATACAYY; match class: 4	TF:M00213_4	65	1.57e-02
tf	Factor: ADR1; motif: NGGRGK; match class: 0	TF:M00048_0	924	2.07e-03
