# Supplementary material for: Principal component analysis based unsupervised feature extraction applied to budding yeast temporally periodic gene expression
Source: BioData Min. 2016 Jun 29;9:22. doi: 10.1186/s13040-016-0101-9 (PMC4928327; doi:10.1186/s13040-016-0101-9)
Supplement: Additional file 12 — Table S8. Enrichment analyses by g:profiler for YCDC, cdc28-13 cells from the study by Cho et al [20], genes that correspond to Figure 9F to 9J. (PDF 1259 kb) [file 13040_2016_101_MOESM12_ESM.pdf]

source	term name<br>Gene Ontology (Biological process)	term ID	n. of term genes	corrected p-value	A	B	C	D	E
BP	DNA replication checkpoint	GO:0000076	10	4.90e-02			4		4
BP	sulfur amino acid metabolic process	GO:0000096	50	1.57e-02	8				
BP	methionine metabolic process	GO:0006555	42	4.04e-03	8				
BP	regulation of gene expression, epigenetic	GO:0040029	151	2.81e-02					13
BP	anatomical structure homeostasis	GO:0060249	78	1.22e-05			11		13
BP	response to stimulus	GO:0050896	1148	1.49e-04		45	51		51
BP	response to stress	GO:0006950	730	1.92e-06	36	36	42	33	41
BP	reproduction	GO:0000003	486	8.31e-09		33	36	27	37
BP	reproduction of a single-celled organism	GO:0032505	203	9.67e-03			15		16
BP	asexual reproduction	GO:0019954	60	6.18e-03					9
BP	biological regulation	GO:0065007	1791	3.17e-06		61	70		72
BP	regulation of biological quality	GO:0065008	477	1.35e-02			26		25
BP	regulation of biological process	GO:0050789	1576	7.04e-04		55	59		61
BP	regulation of metabolic process	GO:0019222	1194	4.62e-02					46
BP	cellular component organization or biogenesis	GO:0071840	2397	5.45e-06		75	84	77	86
BP	cellular component organization	GO:0016043	2045	4.56e-09		73	82	72	84
BP	single-organism process	GO:0044699	3755	2.03e-09		110	119	116	119
BP	cellular process	GO:0009987	4884	1.05e-05		123	130	125	129
BP	cellular response to stimulus	GO:0051716	983	6.85e-06		41	49		49
BP	cellular response to stress	GO:0033554	631	7.47e-08		31	41	31	40
BP	cellular response to DNA damage stimulus	GO:0006974	331	1.27e-11	23	24	34	26	33
BP	single-organism cellular process	GO:0044763	3397	9.14e-14		105	119	111	119
BP	cell division	GO:0051301	355	1.92e-18	25	31	43	33	42
BP	cell budding	GO:0007114	60	6.18e-03					9
BP	cell cycle	GO:0007049	741	6.67e-34	43	60	77	65	73
BP	cell cycle process	GO:0022402	629	9.77e-33	35	50	71	57	68
BP	cell cycle phase transition	GO:0044770	140	3.93e-04		13	15	13	14
BP	cell cycle G2/M phase transition	GO:0044839	37	8.72e-04		8			
BP	cell cycle checkpoint	GO:0000075	85	3.78e-06			14		13
BP	meiotic cell cycle	GO:0051321	303	1.62e-04		21	23	20	23
BP	meiotic cell cycle process	GO:1903046	236	1.98e-04		18	18		20
BP	mitotic cell cycle	GO:0000278	379	3.17e-29	28	38	55	45	53
BP	mitotic cell cycle process	GO:1903047	367	6.85e-29	26	36	54	44	52
BP	regulation of transcription involved in G1/S transition of mi ...	GO:0000083	27	2.38e-02			6		6
BP	mitotic cell cycle phase transition	GO:0044772	137	2.95e-04		13	15	13	14
BP	G2/M transition of mitotic cell cycle	GO:0000086	36	6.96e-04		8			
BP	microtubule-based process	GO:0007017	122	1.31e-08		15	17	17	19
BP	chromosome segregation	GO:0007059	199	1.47e-15		17	31	30	31
BP	external encapsulating structure organization	GO:0045229	212	2.50e-02	16				
BP	cell wall organization	GO:0071555	212	2.50e-02	16				
BP	organelle organization	GO:0006996	1491	1.09e-12		59	72	61	76
BP	organelle fission	GO:0048285	351	1.21e-18	23	32	43	39	42
BP	nuclear division	GO:0000280	341	3.34e-18	23	31	42	39	42
BP	meiotic nuclear division	GO:0007126	170	4.56e-06		17	18	17	19
BP	single-organism organelle organization	GO:1902589	1007	9.55e-16		46	64	53	66
BP	mitotic nuclear division	GO:0007067	215	1.50e-16	18	22	33	32	33
BP	meiosis I	GO:0007127	98	2.89e-06	11	12	13	11	15
BP	chromosome organization	GO:0051276	523	1.67e-17	27	32	47	39	50
BP	DNA conformation change	GO:0071103	90	8.18e-06			11		14
BP	DNA packaging	GO:0006323	57	3.98e-03					9
BP	sister chromatid segregation	GO:0000819	112	1.24e-13		13	23	21	23
BP	mitotic sister chromatid segregation	GO:0000070	104	4.32e-12		12	21	19	21
BP	sister chromatid cohesion	GO:0007062	49	6.22e-11			15	13	15
BP	mitotic sister chromatid cohesion	GO:0007064	38	6.37e-10			12	10	13
BP	telomere organization	GO:0032200	79	1.38e-06			11		14
BP	cytoskeleton organization	GO:0007010	243	1.71e-09		20	25	20	27
BP	septin cytoskeleton organization	GO:0032185	29	3.69e-02					6
BP	septin ring organization	GO:0031106	28	2.98e-02					6
BP	microtubule cytoskeleton organization	GO:0000226	111	2.29e-09		15	17	16	19
BP	microtubule polymerization or depolymerization	GO:0031109	37	8.00e-05		8	9	8	9
BP	microtubule polymerization	GO:0046785	31	4.21e-03		6	7		7
BP	microtubule organizing center organization	GO:0031023	32	2.60e-04		8		7	8
BP	spindle pole body organization	GO:0051300	32	2.60e-04		8		7	8
BP	spindle pole body separation	GO:0000073	13	1.39e-04		6		6	
BP	spindle organization	GO:0007051	40	2.21e-03				8	7
BP	mitotic spindle organization	GO:0007052	34	8.15e-03				7	7
BP	microtubule nucleation	GO:0007020	18	1.76e-03		5	6		6
BP	regulation of cellular process	GO:0050794	1506	8.25e-04			57		59
BP	regulation of cell division	GO:0051302	127	2.31e-02					12
BP	regulation of nuclear division	GO:0051783	118	1.08e-02					12
BP	regulation of cell cycle	GO:0051726	214	3.84e-05		15	19		20
BP	regulation of cell cycle process	GO:0010564	154	7.15e-03			13		14
BP	regulation of spindle pole body separation	GO:0010695	9	2.48e-02		4			
BP	regulation of mitotic cell cycle	GO:0007346	121	3.83e-04			13		14
BP	negative regulation of biological process	GO:0048519	554	2.25e-05		27	33		34
BP	negative regulation of cellular process	GO:0048523	527	2.35e-05			32		33
BP	negative regulation of cell cycle	GO:0045786	97	1.81e-04			13		12
BP	negative regulation of mitotic cell cycle	GO:0045930	64	1.15e-05			12		11
BP	mitotic cell cycle checkpoint	GO:0007093	57	3.57e-05			11		10
BP	morphogenesis checkpoint	GO:0044879	4	2.59e-04		3	4		4
BP	negative regulation of metabolic process	GO:0009892	444	1.08e-03			26		27
BP	negative regulation of nitrogen compound metabolic process	GO:0051172	309	1.36e-02					20
BP	single-organism metabolic process	GO:0044710	2051	3.46e-03			71		71
BP	negative regulation of gene expression, epigenetic	GO:0045814	150	2.62e-02					13
BP	sulfate reduction	GO:0019419	3	1.25e-02	3				
BP	sulfate assimilation, phosphoadenylyl sulfate reduction by phosphoaden ...	GO:0019379	3	1.25e-02	3				
BP	negative regulation of macromolecule metabolic process	GO:0010605	399	1.70e-03			24		25
BP	regulation of cellular metabolic process	GO:0031323	1086	4.82e-02					43
BP	negative regulation of cellular metabolic process	GO:0031324	430	1.99e-03			25		26
BP	negative regulation of nucleobase-containing compound metabolic process	GO:0045934	304	1.07e-02					20
BP	DNA metabolic process	GO:0006259	542	1.21e-13	30	35	41	34	46
BP	DNA strand elongation	GO:0022616	37	1.18e-03			8		8
BP	heteroduplex formation	GO:0030491	9	6.51e-04	4	4	5	4	5
BP	regulation of DNA metabolic process	GO:0051052	107	5.82e-04			12		13
BP	negative regulation of DNA metabolic process	GO:0051053	41	2.70e-03			8		7
BP	DNA recombination	GO:0006310	241	3.62e-07		18	24	19	23
BP	reciprocal DNA recombination	GO:0035825	60	6.61e-04		9	10		10
BP	reciprocal meiotic recombination	GO:0007131	60	6.61e-04		9	10		10
BP	regulation of DNA recombination	GO:0000018	27	1.53e-03			7		6
BP	mitotic recombination	GO:0006312	58	4.75e-04		8	10	8	10
BP	negative regulation of DNA recombination	GO:0045910	16	1.99e-02			5		
BP	DNA replication	GO:0006260	211	4.11e-10		17	22	20	26
BP	DNA-dependent DNA replication	GO:0006261	122	1.25e-10		15	20	18	21
BP	DNA strand elongation involved in DNA replication	GO:0006271	34	5.84e-04			8		8
BP	lagging strand elongation	GO:0006273	18	1.76e-03			5		6
BP	DNA topological change	GO:0006265	10	4.90e-02			4		4
BP	DNA replication initiation	GO:0006270	53	2.00e-02					8
BP	maintenance of DNA repeat elements	GO:0043570	23	6.83e-07		7	9		9
BP	maintenance of DNA trinucleotide repeats	GO:0035753	8	1.40e-02		4	4		4
BP	DNA repair	GO:0006281	284	6.43e-12	20	22	32	24	32
BP	base-excision repair	GO:0006284	18	3.77e-02			5		5
BP	double-strand break repair	GO:0006302	109	1.70e-07		12	17	12	17
BP	recombinational repair	GO:0000725	67	1.91e-03			10		9
BP	double-strand break repair via homologous recombination	GO:0000724	59	5.35e-03			9		8
BP	telomere maintenance	GO:0000723	78	1.22e-05			11		13
BP	telomere maintenance via recombination	GO:0000722	34	8.15e-03			7		7
BP	gene silencing	GO:0016458	154	3.47e-02					13
BP	chromatin silencing	GO:0006342	150	2.62e-02					13
BP	regulation of cell shape	GO:0008360	16	1.99e-02			5		

source	term name<br>Gene Ontology (Cellular component)	term ID	n. of term genes	corrected p-value	A	B	C	D	E
CC	MBF transcription complex	GO:0030907	4	2.59e-04			4		4
CC	extracellular region	GO:0005576	97	5.31e-16	24				
CC	site of polarized growth	GO:0030427	255	3.94e-04		20	17		19
CC	cellular bud	GO:0005933	219	9.53e-07	18	22	18		19
CC	cellular bud neck	GO:0005935	168	8.48e-05		17	16		17
CC	SBF transcription complex	GO:0033309	4	4.71e-02			3		3
CC	anchored component of membrane	GO:0031225	65	1.87e-03	10				
CC	cell periphery	GO:0071944	661	1.40e-04	37				
CC	external encapsulating structure	GO:0030312	106	1.01e-12	22				
CC	cell wall	GO:0005618	106	1.01e-12	22				
CC	fungal-type cell wall	GO:0009277	99	3.02e-12	21				
CC	macromolecular complex	GO:0032991	2124	3.02e-02			70		
CC	protein-DNA complex	GO:0032993	61	7.50e-05	10	10	10	9	11
CC	protein complex	GO:0043234	1484	3.26e-08			68	58	64
CC	DNA packaging complex	GO:0044815	17	1.39e-03	6				
CC	DNA bending complex	GO:1990104	12	1.14e-04	6				
CC	membrane-enclosed lumen	GO:0031974	1132	4.55e-03					47
CC	non-membrane-bounded organelle	GO:0043228	1276	6.21e-11		50	63	51	67
CC	intracellular non-membrane-bounded organelle	GO:0043232	1276	6.21e-11		50	63	51	67
CC	cytoskeleton	GO:0005856	233	2.84e-08		21	25	20	24
CC	microtubule cytoskeleton	GO:0015630	123	1.52e-08		15	19	16	18
CC	chromosome	GO:0005694	387	1.47e-23		32	45	37	50
CC	condensed chromosome	GO:0000793	114	3.03e-11		15	21	20	20
CC	chromosomal part	GO:0044427	351	1.49e-20		27	41	34	45
CC	chromosomal region	GO:0098687	143	2.91e-13		13	22	19	25
CC	chromosome, centromeric region	GO:0000775	89	5.71e-09			16	15	17
CC	condensed chromosome, centromeric region	GO:0000779	64	1.15e-05			12	12	11
CC	chromosome, telomeric region	GO:0000781	54	2.31e-02					8
CC	cohesin complex	GO:0008278	4	2.59e-04			4	3	4
CC	mitotic cohesin complex	GO:0030892	4	2.59e-04			4	3	4
CC	replication fork	GO:0005657	56	5.63e-10	10	11	14	11	15
CC	replisome	GO:0030894	14	3.06e-04		5	5	5	6
CC	kinetochore	GO:0000776	65	1.39e-05			12	12	11
CC	condensed chromosome kinetochore	GO:0000777	58	3.50e-06			12	12	11
CC	MIS12/MIND type complex	GO:0000444	4	4.71e-02			3	3	3
CC	nucleosome	GO:0000786	12	1.14e-04	6				
CC	cytoskeletal part	GO:0044430	218	2.79e-07		21	23	19	22
CC	spindle	GO:0005819	100	4.08e-08		13	17	15	17
CC	microtubule organizing center	GO:0005815	64	1.23e-03			9		10
CC	spindle pole	GO:0000922	74	6.26e-06		10	12	10	13
CC	spindle pole body	GO:0005816	64	1.23e-03			9		10
CC	microtubule	GO:0005874	63	1.06e-03			10	9	
CC	spindle microtubule	GO:0005876	17	2.77e-02			5	5	
CC	organelle lumen	GO:0043233	1074	9.74e-04			43		47
CC	intracellular organelle lumen	GO:0070013	1074	9.74e-04			43		47
CC	microtubule organizing center part	GO:0044450	15	1.39e-02					5
CC	nucleus	GO:0005634	2303	4.87e-09			84	79	90
CC	nuclear part	GO:0044428	1227	1.88e-04			50		53
CC	nuclear lumen	GO:0031981	816	4.46e-07			42		46
CC	nuclear chromosome	GO:0000228	275	6.34e-21	19	28	38	30	41
CC	nuclear chromosome part	GO:0044454	248	1.61e-18	17	23	34	28	37
CC	nuclear replication fork	GO:0043596	44	1.08e-07	8	8	10	8	12
CC	nuclear replisome	GO:0043601	14	3.06e-04		5	5	5	6
CC	DNA replication factor A complex	GO:0005662	3	1.02e-02	3	3	3		3
CC	nuclear nucleosome	GO:0000788	9	7.32e-04	5				
CC	condensed nuclear chromosome	GO:0000794	96	7.65e-13		15	21	19	20
CC	nuclear cohesin complex	GO:0000798	4	2.59e-04			4	3	4
CC	nuclear mitotic cohesin complex	GO:0034990	4	2.59e-04			4	3	4
CC	condensed nuclear chromosome, centromeric region	GO:0000780	54	1.45e-06			12	12	11
CC	condensed nuclear chromosome kinetochore	GO:0000778	47	4.06e-06			11	11	10
CC	nuclear MIS12/MIND complex	GO:0000818	4	4.71e-02			3	3	3
CC	septin ring	GO:0005940	15	1.39e-02			5		

source	term name<br>Gene Ontology (Molecular function)	term ID	n. of term genes	corrected p-value	A	B	C	D	E
MF	structural constituent of cytoskeleton	GO:0005200	29	2.59e-03		6	7		7
MF	structural constituent of cell wall	GO:0005199	16	9.16e-04	6				
MF	DNA binding	GO:0003677	579	7.46e-04			30		32
MF	structure-specific DNA binding	GO:0043566	92	7.52e-04		11	11		12
MF	double-stranded DNA binding	GO:0003690	44	2.94e-05		9	9		10
MF	cyclin-dependent protein serine/threonine kinase regulator activity	GO:0016538	26	2.18e-02	6				

source	term name<br>Biological pathways (KEGG)	term ID	n. of term genes	corrected p-value	A	B	C	D	E
ke	Meiosis - yeast	KEGG:04113	130	2.74e-05		10	14	14	16
ke	Sulfur metabolism	KEGG:00920	15	1.96e-02	4				
ke	Base excision repair	KEGG:03410	18	2.71e-04		5	6	5	6
ke	Mismatch repair	KEGG:03430	20	8.41e-08	5	6	9		7
ke	DNA replication	KEGG:03030	31	5.10e-06	5	7	9	8	9
ke	Cell cycle - yeast	KEGG:04111	126	6.83e-14	16	13	23	19	25
ke	Nucleotide excision repair	KEGG:03420	37	4.23e-04			8	6	6

source	term name<br>Biological pathways (Reactome)	term ID	n. of term genes	corrected p-value	A	B	C	D	E
re	DNA Replication	REAC:5719403	67	5.10e-03			9	9	9
re	Cell Cycle	REAC:5719402	113	3.28e-04		12	12	13	13
re	Chromosome Maintenance	REAC:5719707	14	1.43e-04			5	6	5
re	Telomere Maintenance	REAC:5719706	14	1.43e-04			5	6	5
re	Extension of Telomeres	REAC:5719705	14	1.43e-04			5	6	5
re	Telomere C-strand (Lagging Strand) Synthesis	REAC:5719746	12	4.59e-05			5	6	5
re	Telomere C-strand synthesis initiation	REAC:5719745	6	4.49e-02				3	3
re	Polymerase switching on the C-strand of the telomere	REAC:5719747	10	1.12e-02			4	4	4
re	Cell Cycle, Mitotic	REAC:5719401	101	3.28e-04		12	11	13	12
re	S Phase	REAC:5719400	68	5.77e-03			9	9	9
re	Synthesis of DNA	REAC:5719399	67	5.10e-03			9	9	9
re	DNA strand elongation	REAC:5719415	26	1.86e-05	5	6	8	7	8
re	Lagging Strand Synthesis	REAC:5719416	16	7.39e-06		5	7	6	7
re	Processive synthesis on the lagging strand	REAC:5719417	11	1.43e-05	4	5	5	4	6
re	Removal of the Flap Intermediate	REAC:5719418	11	1.43e-05	4	5	5	4	6
re	Leading Strand Synthesis	REAC:5719414	10	1.12e-02			4	4	4
re	Polymerase switching	REAC:5719413	10	1.12e-02			4	4	4
re	DNA replication initiation	REAC:5719398	6	4.49e-02				3	3
re	Mitotic G1-G1/S phases	REAC:5719408	21	6.82e-05	5	7	5	7	7
re	G1/S Transition	REAC:5719407	21	6.82e-05	5	7	5	7	7
re	E2F mediated regulation of DNA replication	REAC:5719680	19	7.15e-04	5	6		5	6
re	G1/S-Specific Transcription	REAC:5719922	9	6.83e-03		4	4	4	4
re	Mitotic G2-G2/M phases	REAC:5719696	14	3.63e-02	4				
re	G2/M Transition	REAC:5719695	14	3.63e-02	4				
re	Cyclin A/B1 associated events during G2/M transition	REAC:5719730	14	3.63e-02	4				
re	M/G1 Transition	REAC:5719406	6	4.49e-02				3	3
re	DNA Replication Pre-Initiation	REAC:5719405	6	4.49e-02				3	3
re	Activation of the pre-replicative complex	REAC:5719404	6	4.49e-02				3	3
re	Attenuation phase	REAC:5720061	6	3.42e-02	3				
re	Mitotic Prophase	REAC:5720016	11	1.27e-02	4				
re	DNA Repair	REAC:5719501	66	6.66e-04			10		9
re	DNA Damage Bypass	REAC:5719649	19	1.34e-02			5		
re	Translesion synthesis by Y family DNA polymerases bypasses lesions o ...	REAC:5719648	19	1.34e-02			5		
re	Translesion synthesis by POLK	REAC:5720079	14	2.56e-03			5		4
re	Termination of translesion DNA synthesis	REAC:5720077	15	3.76e-03			5		
re	Translesion synthesis by REV1	REAC:5719647	13	1.68e-03		4	5	4	4
re	Translesion synthesis by POLI	REAC:5720080	14	2.56e-03			5		4
re	Translesion Synthesis by POLH	REAC:5719650	16	5.36e-03			5		

source	term name<br>Regulatory motifs in DNA (TRANSFAC TFBS)	term ID	n. of term genes	corrected p-value	A	B	C	D	E
tf	Factor: MCM1; motif: WTTCCYAAWNNGGTAA; match class: 4	TF:M00125_4	198	1.93e-08	21	13		19	
tf	Factor: MCM1; motif: WTTCCYAAWNNGGTAA; match class: 3	TF:M00125_3	161	2.25e-08	19			15	
tf	Factor: MCM1; motif: WTTCCYAAWNNGGTAA; match class: 2	TF:M00125_2	82	2.34e-04	10			9	
tf	Factor: HSF; motif: AGAANNTTCTAGAAN; match class: 0	TF:M00169_0	42	7.12e-03	6				
tf	Factor: MCM1+SFF; motif: TTWCCYAAWNNGGAAAWWNGTAAAYAA; match class: 4	TF:M01051_4	26	5.74e-03	5	4	4		
tf	Factor: MCM1+SFF; motif: TTWCCYAAWNNGGAAAWWNGTAAAYAA; match class: 3	TF:M01051_3	19	1.36e-02	4	4			
tf	Factor: HSF; motif: AGAANNTTCTAGAAN; match class: 3	TF:M00169_3	264	1.30e-02	15				
