# Supplementary material for: Principal component analysis based unsupervised feature extraction applied to budding yeast temporally periodic gene expression
Source: BioData Min. 2016 Jun 29;9:22. doi: 10.1186/s13040-016-0101-9 (PMC4928327; doi:10.1186/s13040-016-0101-9)
Supplement: Additional file 13 — Document S2. Performances of four other unsupervised clustering methods. (PDF 252 kb) [file 13040_2016_101_MOESM13_ESM.pdf]

Comparison with other (unsupervised) clustering methods

Y-h. Taguchi, Department of Physics, Chuo University, Tokyo, Japan

Contents

1 Introduction **1**

2 Methods **1**

2.1 SOM 1
2.2 HC 1
2.3 KM 2
2.4 WGCNA 2
2.5 Hierarchical clustering of representative profiles of each cluster together with PC loadings 2
2.6 Computation of representative profiles 2
2.7 Regression analysis of representative profiles using multiple PC loadings 2

3 Results **3**

3.1 SOM 3
3.2 HC 5
3.3 KM 7
3.4 WGCNA 9

1 Introduction

To demonstrate the superiority of PCA based unsupervised FE over other (unsupervised) clustering methods, we compared the performances of PCA based unsupervised FE with that of multiple popular clustering methods including self-organized map (SOM), hierarchical clustering (HC), K-means (KM) and weighted correlation network analysis (WGCNA). In the following studies, all profiles were normalized to have a mean of 0 and a variance of 1 within each sample (time point) as described in the main text before clustering was performed,

$$x_{ij}^{(j)} \equiv \frac{x_{ij} - \langle x_{i'j} \rangle_{i'}}{\sqrt{\left\langle (x_{i"j} - \langle x_{i'j} \rangle_{i'})^2 \right\rangle_{i"}}}$$

where

$$\langle A_i \rangle_i \equiv \sum_{i=1}^{N} \frac{A_i}{N}$$

where N is the number of genes (probes). Superscript "(j)" suggested that normalization was performed with jth sample (time point).

2 Methods

2.1 SOM

SOM was performed using `som` functions included in `som` package [1] in R [2]. SOM was applied to normalized profiles (mean of 0 and variance of 1 within each sample (time point)) assuming either a two times two (2×2) square lattice or a three times three (3×3) square lattice. Profiles that represented each cell were extracted from the element named `code` in the output from `som`.

2.2 HC

HC was performed using the `hclust` function in R [2]. The negative signed Pearson's correlation coefficients between expression profiles of ith and i'th genes, i.e., $\{x_{ij} \mid j = 1, \ldots, M\}$ and $\{x_{i'j} \mid j = 1, \ldots, M\}$ are used as distance. Unweighted Pair Group Method with Arithmetic mean (UPGMA) was employed as a clustering algorithm by using the setting `method="average"` in `hclust`. Then `cutree` in R [2] was used to obtain n_c clusters ($n_c = 4, 9$) by setting `k=`n_c in `hclust`.

2.3 KM

KM was performed using `kmeans` function in R [2]. The number of cluster n_c was 4 and 9 by setting `center=`n_c for `kmeans`. Initial condition dependence of K-means was compensated by setting `nstart=100`; clustering of the majority among 100 independent trials was employed.

2.4 WGCNA

WGCNA [3] was performed using the `WGCNA` package in R [2]. Although various methodologies/algorithms were implemented in the `WGCNA` package, after preliminary experiments, we decided to employ UPGMA (using `hclust` in R [2]) using topological overlap matrix dissimilarity (TOMdist) computed by `TOMdist` function implemented in `WGCNA` [3] from absolute Pearson's correlation coefficients to the sixth power. After soft connectivities were computed by `softConnectivity` function, only genes (probes) with the top most 3600th connectivities were considered. Then, the `cutreeDynamic` function was used to obtain clusters assigning `cutHeight = 0.65` and `0.75` for $n_c = 4$ and 9, respectively.

2.5 Hierarchical clustering of representative profiles of each cluster together with PC loadings

To determine the coincidence between representative profiles of each cluster and PC loadings, representative profiles were clustered together with PC loadings by hierarchical clustering using the `hclust` function implemented in R [2] employing negative signed absolute Pearson's correlations as distances; UPGMA was employed as a clustering algorithm by setting `method="average"` in `hclust`. Representative profiles were named "CLS" followed with a sequential number that represented each cluster while kth PC loadings were named "PCk" in hierarchical clustering in Figs.

2.6 Computation of representative profiles

Other than SOM, no functions that automatically output representative profiles were implemented. Representative profiles in each cluster c were computed as

$$x_j^c \equiv \langle x_{ij}^{(i)} \rangle_i^c$$

$$x_{ij}^{(i)} \equiv \frac{x_{ij}^{(j)} - \langle x_{ij'}^{(j)} \rangle_{j'}}{\sqrt{\left\langle \left(x_{ij"}^{(j)} - \langle x_{ij'}^{(j)} \rangle_{j'} \right)^2 \right\rangle_{j"}}}$$

where

$$\langle B_j \rangle_j \equiv \sum_{j=1}^{M} \frac{B_j}{M}$$

where M is the number of samples (time points) and

$$\langle A_i \rangle_i^c \equiv \frac{1}{N_c} \sum_{i \in c} A_i$$

and $\sum_{i \in c}$ is the summation of genes i that belong to cluster c and N_c is the number of genes in the cluster c. Superscript "(i)" suggested that normalization was performed with ith feature.

2.7 Regression analysis of representative profiles using multiple PC loadings

Representative profiles are often represented not by individual PC loading but by a linear combination of multiple PC loadings. To evaluate this relationship, we performed regression analysis of representative profiles using a pair of PC loadings, i.e. a pair of PC2 and PC3 loadings or PC1 and PC4 loadings. This was executed by `lm` function in R [2]. Regression coefficients together with 95 % confidence intervals as well as P-values are listed. Correlation coefficients between representative profiles and fitted values given by regression analysis together with associated P-values are also listed.

3 Results

We evaluated how well the representative profiles reproduced four PC loadings (PC1 to PC4 loadings) used for PCA based unsupervised FE using hierarchical clustering by checking if representative profiles were clustered together with PC loadings (see methods). Because PC1 to PC4 loadings were useful for extracting genes related to YMC, if representative profiles are coincident with PC1 to PC4 loadings, this indicates that representative profiles are related to YMC. Other than coincidence with PC1 to PC4 loadings, we also determined whether representative profiles exhibited clear periodicity coincident with cell division cycle as observed in Fig. 2(b). Furthermore, when investigating coincidence with PC1 and PC4, we checked whether representative profiles exhibited periods that were half as long as the cell cycle period, which was identified only by PC1 and PC4. Of note, the cell division cycle corresponds to the time interval spanned by twelve time points, thus representative profiles coincident with cell division cycle should exhibit three periods within 36 observed time points, while representative profiles whose periods are half as long as the cell division cycle should exhibit six periods. Table S1 summarized the outcomes described in the following subsections. Although WGCNA when $n_c = 9$ was the best method for reproducing the results obtained by PCA based unsupervised FE, it still did not generate clusters whose representative profiles had a period half as long as the cell division cycle period.

	$n_c = 4$			$n_c = 9$				
	PC2*	PC3†	PC1/PC4‡	PC2*	PC3†	PC1/PC4‡	PC2 + PC3§	half period¶
SOM	3	—	1,4	2,3,7,8	—	1,4,6,9	—	—
HC	2	4	—	3	4	—	—	—
KM	2	3	4	—	1	3	4,6,7	—
WGCNA	—	4	1,3	7,9	3	5	2,4,8	—

Table S1: Summary of UPGMA of PC loadings and representative profiles. *:clusters whose representative profiles are clustered together with PC2, †:clusters whose representative profiles are clustered together with PC3, ‡:clusters whose representative profiles are clustered together with PC1 and/or PC4, §:clusters whose representative profiles are highly correlated with regression using PC2 and PC3, ¶: clusters whose representative profiles have a period that is half as long as the cell division cycle period.

We did not perform any biological term enrichment analyses for each obtained cluster because each cluster often included more than 1000 genes. Thus, it is not suitable to evaluate biological significance without specifying the limited number of representative genes in each cluster; clusters including more than 1000 genes are unlikely to have significant biological term enrichments, thus are inevitably judged to be biologically unfeasible, but this is clearly not a fair comparison. However, as has been demonstrated in the main text, even if we can successfully obtain biologically feasible profiles, whether we select genes based upon correlation or projection is important. Thus, we decided that evaluating the biological significance of each cluster by biological term enrichment is not a good strategy to compare outcomes between cluster analysis and PCA based unsupervised FE.

3.1 SOM

SOM [4] that aims to cluster profiles onto cells arranged over lattice structure is a commonly used algorithm. Groups of genes that share similar profiles are mapped to the same cell while cells associated with distinct profiles are placed apart from each other. Because a specific lattice structure for SOM needs to be selected and there were at least four PC loadings identified in PCA based unsupervised FE and orthogonal to (thus are apart from) each other, we first assumed a 2×2 square lattice; and second a 3×3 square because nine cells were large enough to cover the eight cells required to separately cluster two PC loadings with opposite signs: 2 (positive or negative sign) times 4 (four critical PC loadings) = 8.

Figs. S2 and S3 shows the UPGMA of PC loadings and representative profiles as well as the profiles themselves ($n_c = 4$ and 9). Representative profiles were clustered together with PC2 loadings (cluster 3 for $n_c = 4$ and clusters 2, 3, 7 and 8 for $n_c = 9$). When $n_c = 4$, SOM identified only one cluster whose representative profiles were coincident with PC2 loadings (cluster 3) while there were four clusters identified with SOM that had representative profiles coincident with PC2 loadings (clusters 2, 3, 7 and 8) when $n_c = 9$. As n_c increased, SOM identified more probes that belonged to the cluster associated with PC2 loadings. As shown in Fig. S3(c), among four clusters clustered together with PC2 loadings when $n_c = 9$, the majorities in clusters 3 and 7 when $n_c = 9$ moved from clusters 3 and 2 when $n_c = 4$, respectively. The reason why clusters 3 and 7 when $n_c = 9$ exhibited clearer coincidence with PC2 loadings is because some probes (34 probes from cluster 3 when $n_c = 4$ and 48 probes from cluster 2 when

$n_c = 4$) were removed from clusters 3 and 2 when $n_c = 4$ and were moved to cluster 5 when $n_c = 9$; this process can be regarded as denoising because cluster 5 when $n_c = 9$ did not exhibit any periodic oscillations. However, some probes were moved to cluster 2 when $n_c = 9$ from clusters 1 and 4, which did not exhibit periodic oscillations when $n_c = 4$; this process can be regarded as signal collecting. The reason why clusters 2 and 3 when $n_c = 9$ cannot be identified when $n_c = 4$ is simple; for clusters 2 and 3 to exhibit clear periodicity, probes without periodic natures must be removed. However, because of the definition of clustering analysis, removed aperiodic profiles must be clustered. This is a clear discrepancy; aperiodic profiles must be removed from clusters that exhibit periodicity but also must be clustered together within any other cluster, but this is impossible because aperiodic profiles that share nothing cannot be clustered together. Therefore, in clustering algorithms including SOM, there cannot be a garbage box, because a garbage box must be also a cluster, but it is impossible for the probes in the garbage box to form a cluster, because they are garbage with no shared (representative) profiles. Thus, if n_c is not large enough, clusters whose representative profiles do not appear periodic must include some periodic profiles with which they can be clustered, while clusters whose representative profiles appear periodic must accept some aperiodic profiles because there are no other clusters to which they can belong. This is the reason why PCA based unsupervised FE can outperform clustering methods. In PCA based unsupervised FE, the region around the origin can work as a garbage box. In Fig. 2(a), grey marks are collected around the origin while those with periodicity are apart from the origin. Thus, there naturally must be a garbage box in PCA based unsupervised FE: the region around the origin. In contrast to each cluster in clustering analysis, probes around the origin do not have to have shared representative profiles, because there are $36 - 2 = 34$ remaining dimensions that accept their diversity. The existence of a garbage box is one advantage of PCA based unsupervised FE.

No profiles coincident with PC3 loadings were identified by SOM when $n_c = 4$ or 9. This might be because aperiodic clusters cannot accept sufficient numbers of aperiodic profiles to produce periodic clusters coincident with PC3 loading, because the overall contribution of PC3 was less than PC2. In addition, no profiles whose period was half of the cell cycle period were identified, although representative profiles of clusters 1 and 4 ($n_c = 4$) and clusters 1, 4, 6, and 9 ($n_c = 9$) were well represented by the linear combination of PC1 and PC4 loadings (Fig. S5 and Table S2) and were clustered together with PC1 and PC4 that exhibited half of the cell cycle period. Representative profiles of these clusters did not exhibit half of the cell cycle period (Fig. S4). The reason for this might be the same as why PC3 loadings cannot be a representative profile in any cluster. Thus, the overall evaluation suggested that SOM was inferior to PCA based unsupervised FE.

(a) (b)

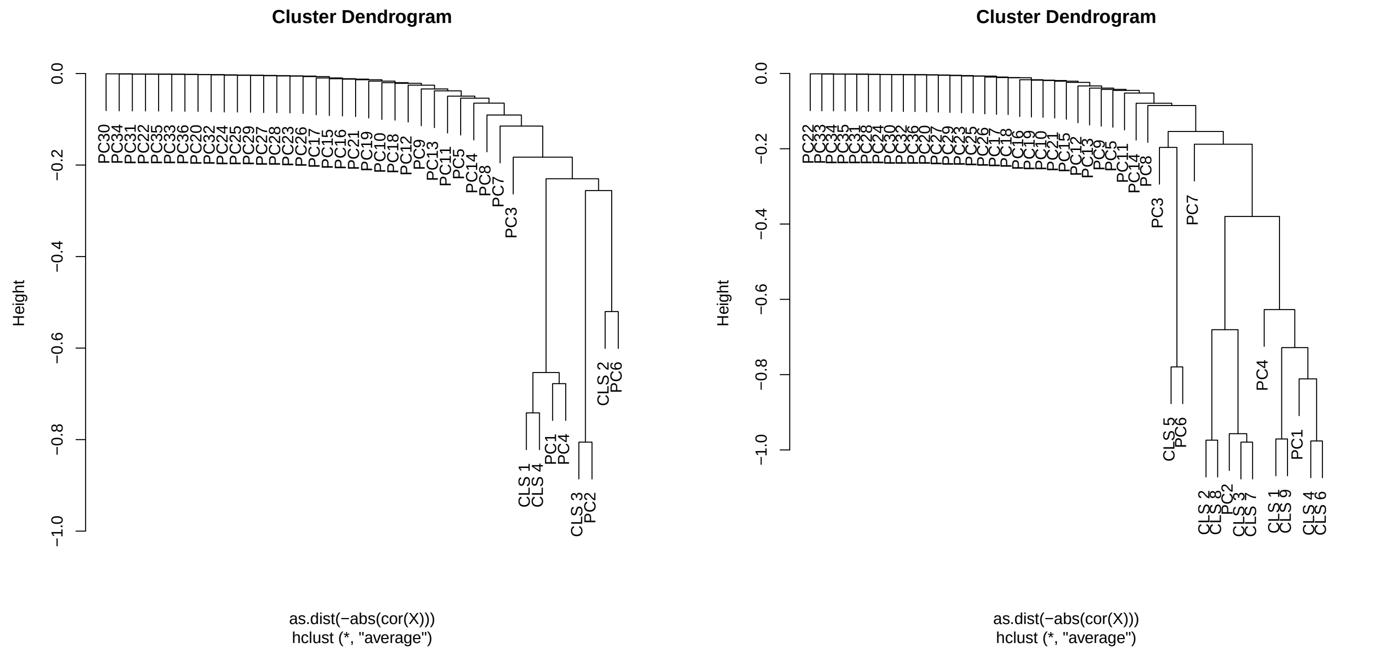

Figure S2: UPGMA of PC loadings and representative profiles of clusters obtained by SOM. (a) $n_c = 4$ (b) $n_c = 9$

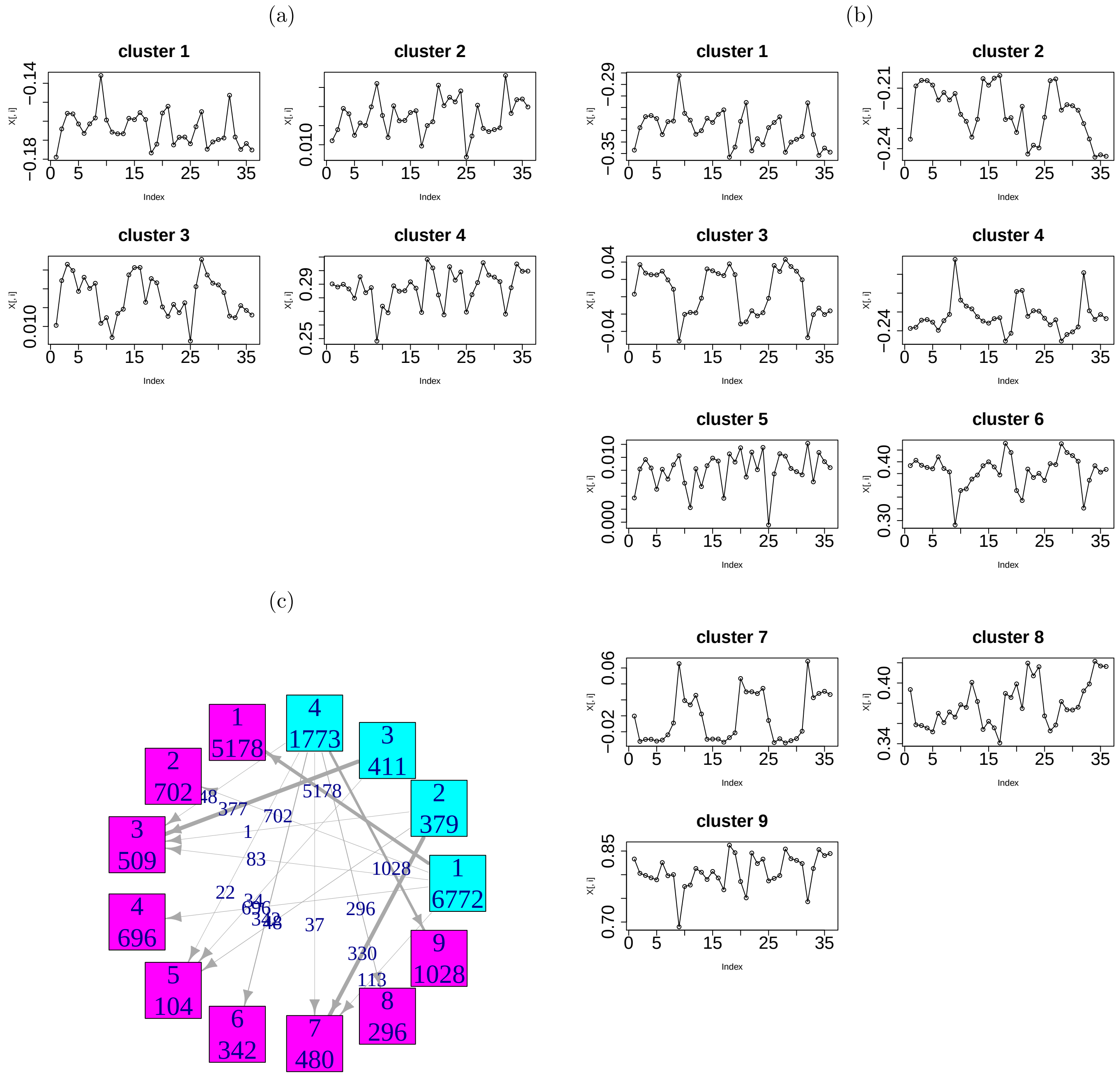

Figure S3: Representative profiles of clusters obtained by SOM (a) $n_c = 4$ (b) $n_c = 9$ (c) Comparison between (a) and (b). Squares filled with cyan and magenta correspond to clusters in (a) and (b), respectively. Upper numbers in squares are the cluster number. Lower numbers in squares are the number of probes in each cluster. Arrows are how probes moved from clusters to clusters when n_c increases from 4 to 9. The numbers associated with arrows are the number of genes that moved between clusters; width of arrow represents the ratio of moving genes to total number of genes in each cluster.

3.2 HC

$n_c = 4, 9$ so that HC had the same number of clusters as SOM. Figs. S6 and S7 show the UPGMA of PC loadings and representative profiles as well as the profiles themselves ($n_c = 4$ and 9). HC was slightly better than SOM. HC

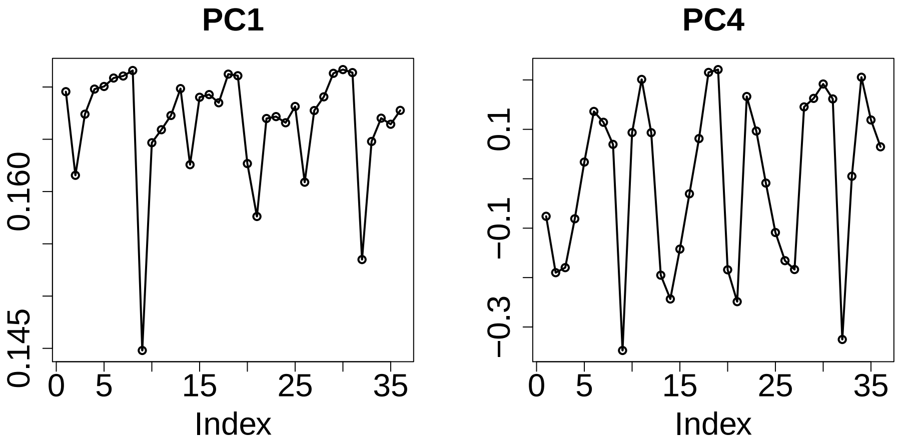

Figure S4: PC loadings PC1 and PC4.

(a) (b)

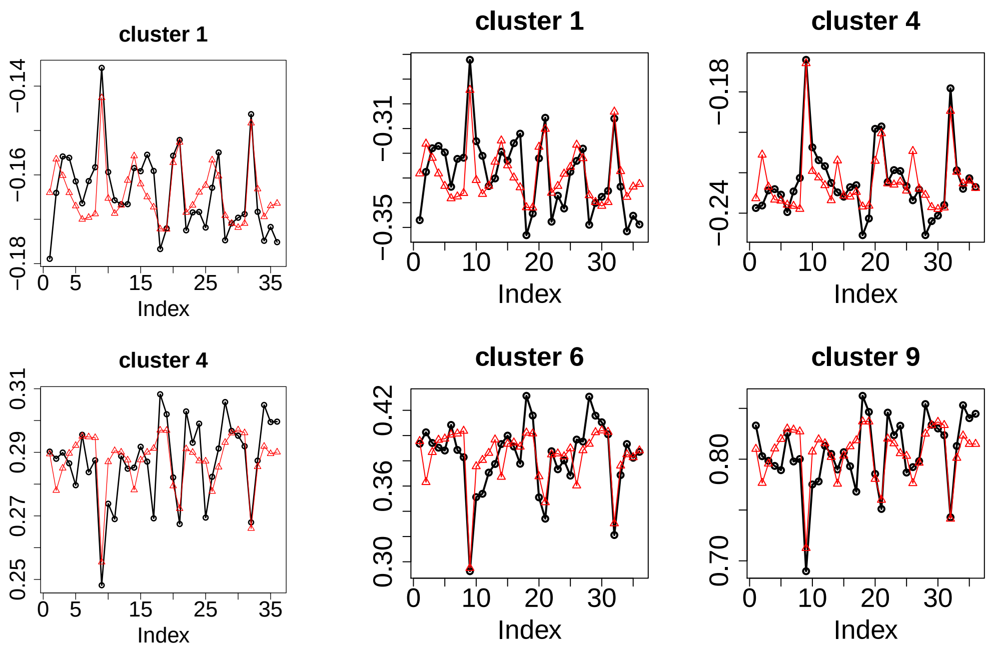

Figure S5: Regression analysis of representative profiles of clusters (a) 1 and 4 identified by SOM when $n_c = 4$ and (b) 1, 4, 6 and 9 identified by SOM when $n_c = 9$ using PC1 and PC4 loadings. Black lines are representative profiles and red lines are regression profiles using PC1 and PC4 loadings (Table S2).

identified two clusters 3 and 4 when $n_c = 9$ whose representative clusters were clustered together with PC2 and PC3 loadings, respectively. Although HC also identified two clusters 2 and 4 when $n_c = 4$ whose representative clusters were clustered together with PC2 and PC3 loadings, respectively, the absolute correlation coefficients are insufficiently large (vertical axis). As shown in Fig. S7(c), cluster 3 when $n_c = 9$ clustered with PC2 loadings with relatively larger absolute correlation coefficients was a part of cluster 1 when $n_c = 4$ whose representative profile exhibited no periodic oscillation. Again, the difficulty of removing aperiodic profiles from periodic profiles and moving them to other clusters that gather aperiodic oscillations (a garbage box) prevented HC from generating a cluster whose representative profiles were clustered together with PC2 loadings. Furthermore, no obtained representative profiles exhibited periodicity coincident with cell division cycle as clearly as PC2 and PC3 loadings did in Fig. 2(b). Moreover, no profiles whose period was half of the cell cycle period were identified similar to SOM. Thus, the overall evaluation suggested that HC was inferior to PCA based unsupervised FE, although HC could identify some clusters whose

Cluster	Coefficients (P-value) PC1	PC4	Correlation Coefs	P-value
		$n_c = 4$		
1	$-0.593 \pm 0.260(2.92 \times 10^{-2})$	$-0.025 \pm 0.009(6.58 \times 10^{-3})$	0.747	1.39×10^{-6}
4	$1.166 \pm 0.404(6.77 \times 10^{-3})$	$0.019 \pm 0.013(1.60 \times 10^{-1})$	0.688	2.55×10^{-5}
		$n_c = 9$		
1	$-0.905 \pm 0.460(5.78 \times 10^{-2})$	$-0.042 \pm 0.015(8.89 \times 10^{-3})$	0.718	6.33×10^{-6}
4	$-2.714 \pm 0.423(2.84 \times 10^{-7})$	$0.002 \pm 0.014(9.11 \times 10^{-1})$	0.832	3.52×10^{-9}
6	$4.066 \pm 0.745(4.72 \times 10^{-6})$	$-0.001 \pm 0.024(9.82 \times 10^{-1})$	0.790	9.61×10^{-8}
9	$3.121 \pm 0.942(2.26 \times 10^{-3})$	$0.075 \pm 0.031(2.13 \times 10^{-2})$	0.791	1.96×10^{-7}

Table S2: Regression analysis of representative profiles of clusters 1 and 4 (1, 4, 6 and 9) identified by SOM when $n_c = 4(9)$ using PC1 and PC4 loadings (Fig. S5). Errors are 95th percentile confidence intervals.

representative profiles were clustered together with PC3 loadings, which was lost in SOM.

(a) (b)

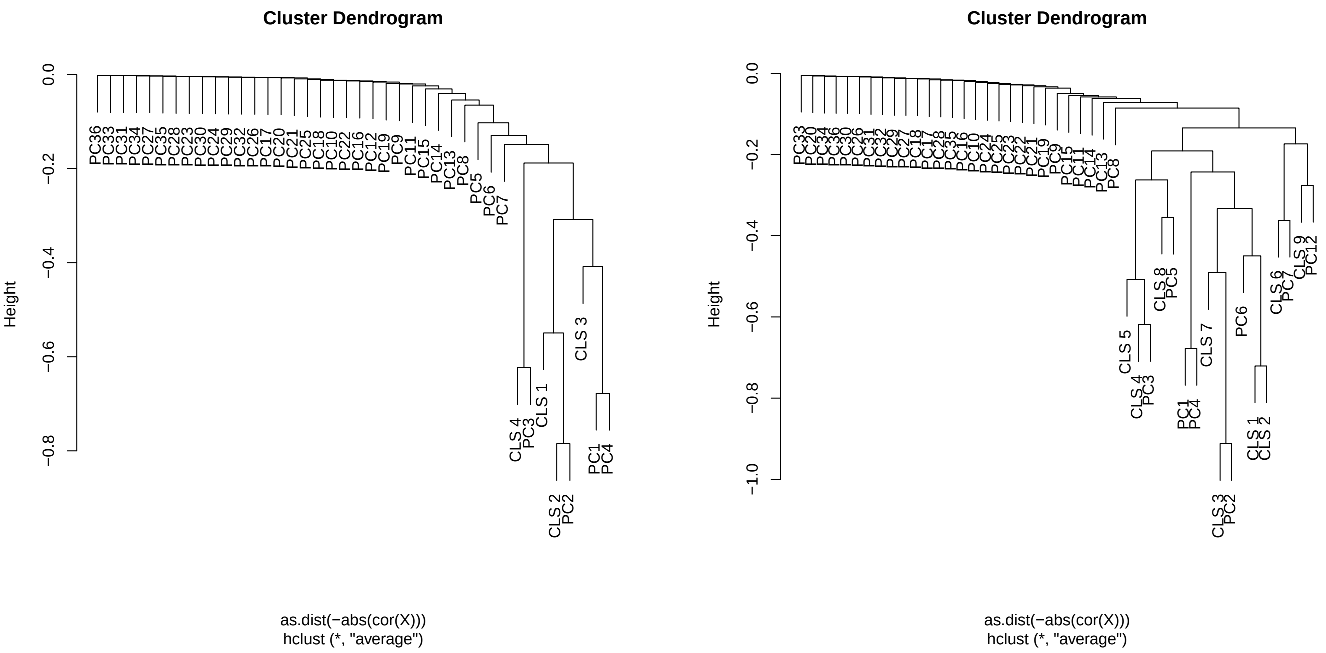

Figure S6: UPGMA of PC loadings and representative profiles of clusters obtained by HC. (a) $n_c = 4$ (b) $n_c = 9$

3.3 KM

$n_c = 4, 9$ so that KM had the same number of clusters as SOM. Figs. S8 and S9 show the UPGMA of PC loadings and representative profiles as well as the profiles themselves ($n_c = 4$ and 9). This method was better than for SOM or HC; three (PC1, PC2, and PC3) loadings had clusters whose representative profiles were clustered together even when $n_c = 4$. This successful achievement of KM was possibly because KM works in the same liner space as PCA. The only difference between PCA and KM is that the former generates new linear space by combining original axes while the latter does not. It is also clear that more representative profiles are coincident with cell division cycle, i.e., those exhibiting three period oscillations, e.g., clusters 2 and 4 when $n_c = 4$ and clusters 1, 2, 3 and 4 when $n_c = 9$. Especially, KM first identified representative profiles clustered together with PC1 and coincident with cell division cycle (cluster 4 when $n_c = 4$ and cluster 3 when $n_c = 9$). Comparison of clusters between $n_c = 4$ and $n_c = 9$ (Fig. S9 (c)) showed a smaller number of edges between clusters, indicating that drastic rearrangements of clusters did not take place between $n_c = 4$ and $n_c = 9$. Thus, KM works relatively well even for smaller numbers of clusters. Cluster 4 when $n_c = 4$ almost directly corresponded to cluster 3 when $n_c = 9$. Cluster 2 when $n_c = 9$ was a subset of cluster 2

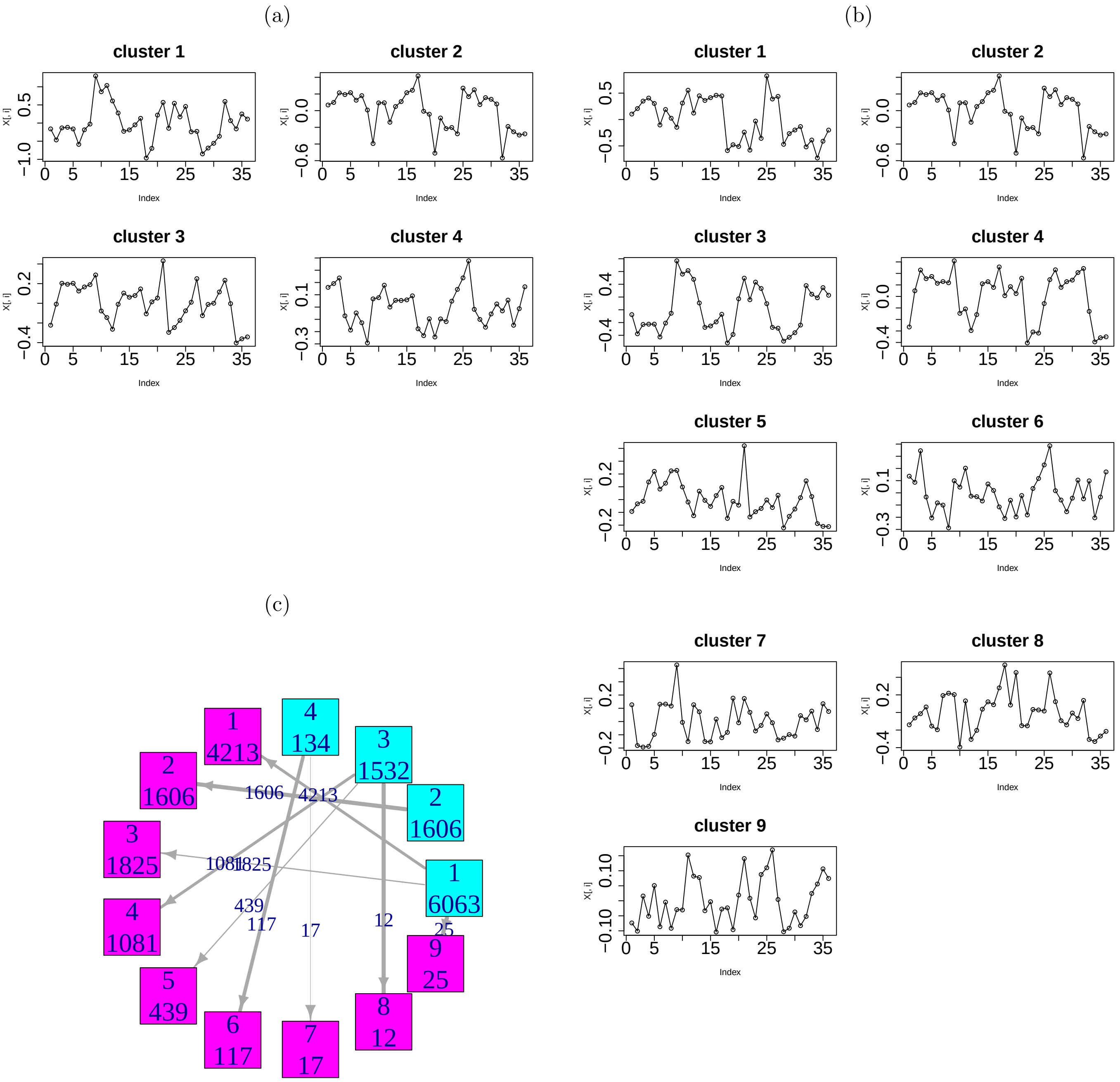

Figure S7: Representative profiles of clusters obtained by HC (a) $n_c = 4$ (b) $n_c = 9$ (c) Comparison between (a) and (b). Squares filled with cyan and magenta correspond to clusters in (a) and (b), respectively. Upper numbers in squares indicate cluster number. Lower numbers in squares indicate the number of probes in each cluster. Arrows are how probes moved from clusters to clusters when n_c increases from 4 to 9. The numbers associated with arrows are the number of genes that moved between clusters; width of arrow represents the ratio of moving genes to total number of genes in each cluster.

when $n_c = 4$ and included more than half of the probes in cluster 2 when $n_c = 4$. Thus, cluster 2 when $n_c = 4$ is also directly related to cluster 2 when $n_c = 9$. Despite these improvements compared with the previous two clusterings, SOM and HC, the problem caused by the lack of garbage box still exists. For example, although cluster 1 when $n_c = 9$

exhibited clear three period oscillations, the probes in it were divided into clusters 4 and 2 when $n_c = 4$. However, a representative profile of cluster 3 when $n_c = 9$ exhibited three relatively clear period oscillations, because it included too many probes (more than 5000 probes), and thus it is unlikely that the majority of probes in cluster 3 when $n_c = 9$ exhibit oscillations coincident with representative profiles. Considering the flatness of the representative protein of cluster 3 when $n_c = 9$, it must accept more aperiodic profiles because of the smaller penalty and had to work as a garbage box. Thus, KM still could not fully resolve the problem of a garbage box.

Interestingly, clusters 4, 6 and 7 when $n_c = 9$ whose representative profiles were clustered together with PC2 loadings did not show a large correlation (less than 0.8) with PC2 loadings. Can we regard these clusters as identification of PC2 loadings by KM even if the correlation is less than 0.8? To address this, we performed regression analysis between representative profiles of clusters 4, 6 and 7 when $n_c = 9$ and PC2, PC3 loadings (Fig. S10 and Table S3). It is obvious that these representative profiles are well represented by the linear combinations of PC2 and PC3 loadings. This suggested these clusters were located on the plane spanned by PC2 and PC3. Cell cycle regulated genes distributed on the plane spanned by PC2 and PC3 were already identified by PCA based unsupervised FE (Fig. 2). This suggested that KM successfully reproduced the findings of the PCA based unsupervised FE. Although KM was more coincident with PC loadings identified than the previous two clustering methods, representative profiles are still less periodic that those in Fig. 2(b) and profiles whose period was half as long as the cell cycle period are not present. Thus, PCA based unsupervised FE was superior to KM.

(a) (b)

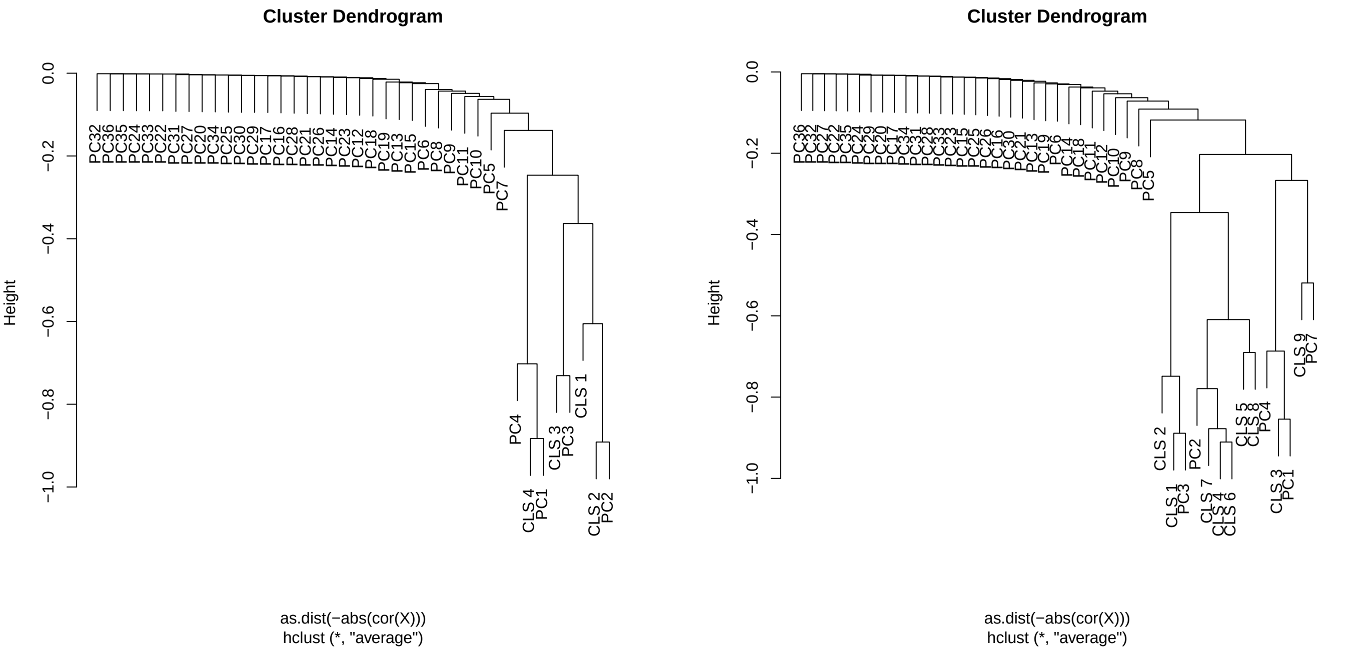

Figure S8: UPGMA of PC loadings and representative profiles of clusters obtained by KM. (a) $n_c = 4$ (b) $n_c = 9$

	Coefficients (P-value)			
Cluster	PC2	PC3	Correlation Coefs	P-value
4	$0.356 \pm 0.035(8.14 \times 10^{-12})$	$-0.206 \pm 0.035(1.27 \times 10^{-6})$	0.900	1.28×10^{-12}
6	$0.583 \pm 0.061(5.36 \times 10^{-11})$	$-0.259 \pm 0.061(1.7 \times 10^{-4})$	0.876	3.59×10^{-11}
7	$-0.250 \pm 0.022(5.31 \times 10^{-13})$	$0.175 \pm 0.022(3.11 \times 10^{-9})$	0.925	1.47×10^{-14}

Table S3: Regression analysis of representative profiles of clusters 4, 6 and 7 identified by KM when $n_c = 9$ using PC2 and PC3 loadings (Fig. S10). Errors are 95th percentile confidence intervals.

3.4 WGCNA

Finally, we evaluated WGCNA (see methods). In contrast to the previous three clustering methods that are general purpose algorithms, WGCNA was proposed specifically to cluster gene expression profiles and has been used widely

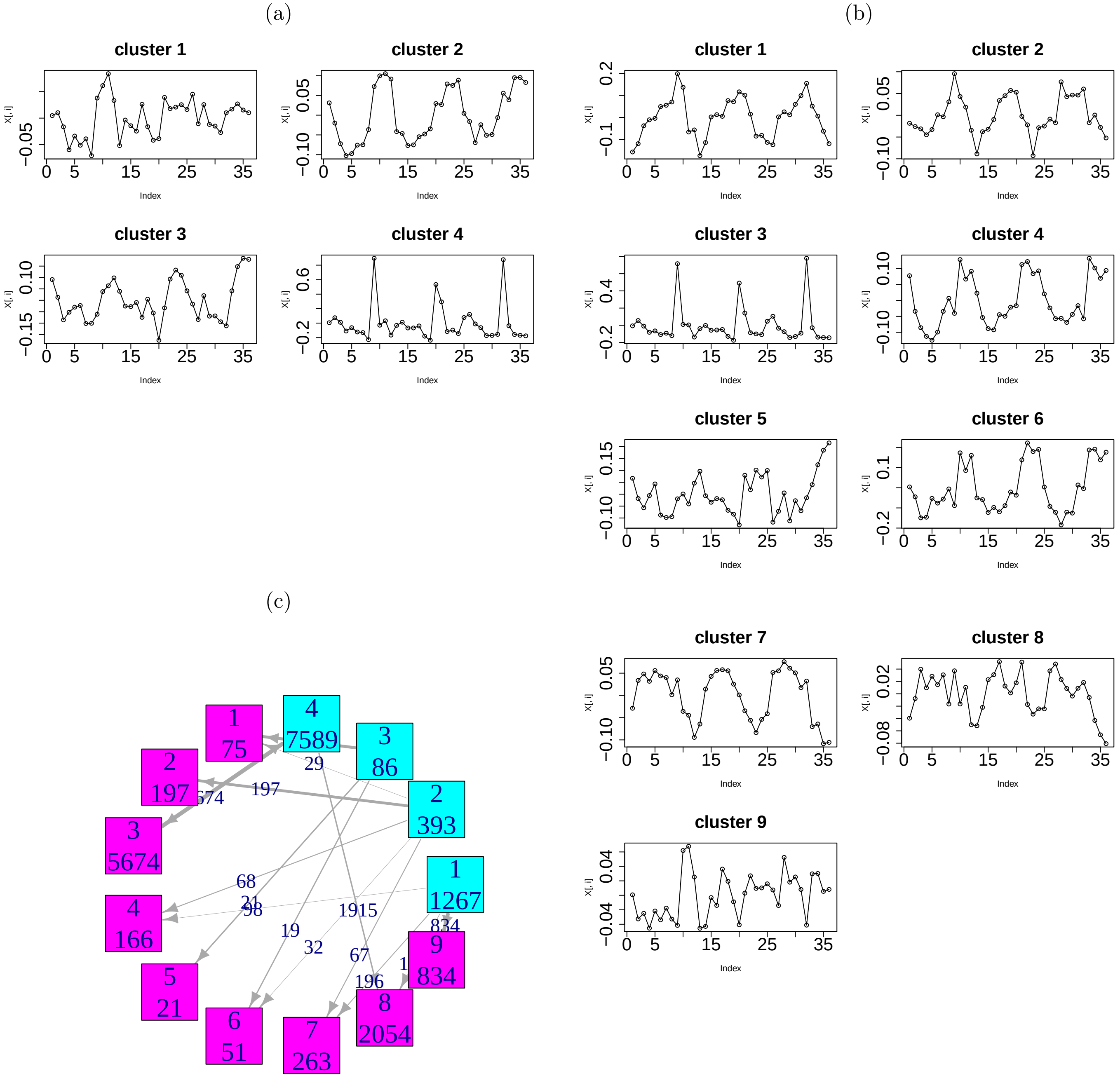

Figure S9: Representative profiles of clusters obtained by KM (a) $n_c = 4$ (b) $n_c = 9$ (c) Comparison between (a) and (b). Squares filled with cyan and magenta correspond to clusters in (a) and (b), respectively. Upper numbers in squares indicate cluster number. Lower numbers in squares indicate the number of probes in each cluster. Arrows are how probes moved from clusters to clusters when n_c increases from 4 to 9. The numbers associated with arrows are the number of genes that moved between clusters; width of arrow represents the ratio of moving genes to total number of genes in each cluster.

among societies. Figs. S11 and S12 indicate the UPGMA of PC loadings and representative profiles as well as the profiles themselves ($n_c = 4$ and 9). The outcomes of WGCNA were distinct from the other three clustering methods. First, when $n_c = 4$ each of the four clusters was clustered with each of PC1 to PC4 (Fig. S11(a)). Thus,

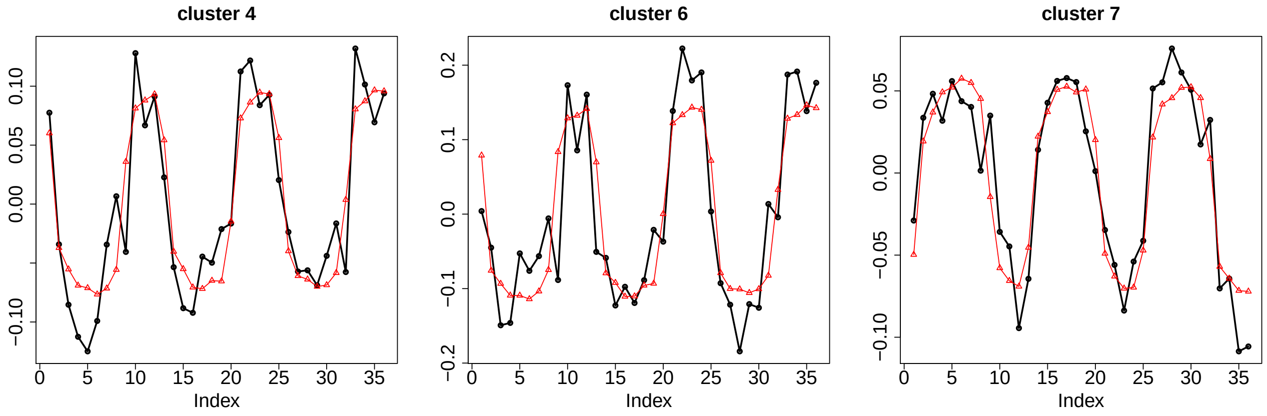

Figure S10: Regression analysis of representative profiles of clusters 4, 6 and 7 identified by KM when $n_c = 9$ using PC2 and PC3 loadings. Black lines are representative profiles and red lines are regression profiles using PC2 and PC3 loadings (Table S3).

potentially, each cluster corresponds to each PC loading. Despite this, no representative profiles exhibited clear periodic oscillations. Thus, these four clusters were a mixture of profiles coincident with PC1 to PC4 and aperiodic profiles. As expected, when $n_c = 9$, many clusters whose representative profiles were correctly periodic. Cluster 3 had a representative profile clustered with PC3 loadings while clusters 7 and 9 were clustered with PC2 loadings when $n_c = 9$. The shapes of the profiles were also highly coincident with the cell division cycle because they exhibited three period oscillations. The representative profiles of clusters 2, 4, and 8 were also represented by the linear combinations of PC2 and PC3 loadings (Fig. S13 and Table S4). The remaining clusters 1, 5 and 6 exhibited no periodic nature and functioned as a garbage box. Thus, the outcomes of WGCNA were mostly coincident with those of PCA based unsupervised FE, i.e., a mixture of garbage (gray marks in Fig. 2(a)) and cell cycle regulated genes (black, red, and green marks in Fig. 2(a)).

Using WGCNA, we obtained outcomes similarly coincident with PCA based unsupervised FE. However, the representative profiles did not exhibit periodicity as clear as those in Fig. 2(b). WGCNA also failed to detect profiles whose period was half as long as the cell division cycle. If n_c increases further, can WGCNA detect clusters whose representative profile has a period half as long as the cell division cycle? However, WGCNA does not always work as expected, for example clusters 2, 4, and 8 were distinctly clustered although these three shared almost similar representative profiles (regression coefficients in Table S4 were almost identical). Moreover, although the representative profiles of clusters 2, 4 and 8 were expressed as linear combinations of PC2 and PC3 loadings, it would be difficult to recognize whether the analysis by PCA based unsupervised FE was not performed in advance.

In summary, although WGCNA could reproduce some features obtained by PCA based unsupervised FE, PCA based unsupervised FE still outperformed WGCNA, which is a frequently used and *de facto* standard methodology for gene expression profile clustering.

	Coefficients (P-value)			
Cluster	PC2	PC3	Correlation Coefs	P-value
2	$-1.500 \pm 0.208 (2.74 \times 10^{-8})$	$-0.636 \pm 0.208 (1.27 \times 10^{-6})$	0.807	2.82×10^{-8}
4	$-1.197 \pm 0.093 (1.91 \times 10^{-14})$	$-0.453 \pm 0.093 (2.61 \times 10^{-5})$	0.869	1.98×10^{-14}
8	$-0.810 \pm 0.108 (1.21 \times 10^{-8})$	$-0.724 \pm 0.108 (1.17 \times 10^{-7})$	0.923	8.30×10^{-11}

Table S4: Regression analysis of representative profiles of clusters 2, 4 and 8 identified by WGCNA when $n_c = 9$ using PC2 and PC3 loadings (Fig. S13). Errors are 95th percentile confidence intervals.

Conclusion

We compared the outcome of four clustering algorithms with those of PCA based unsupervised FE. Among those compared, the WGCNA performance was most coincident with PCA based unsupervised FE, but was still inferior to PCA based unsupervised FE. The main difficulty is that no clustering method can cluster aperiodic features into a

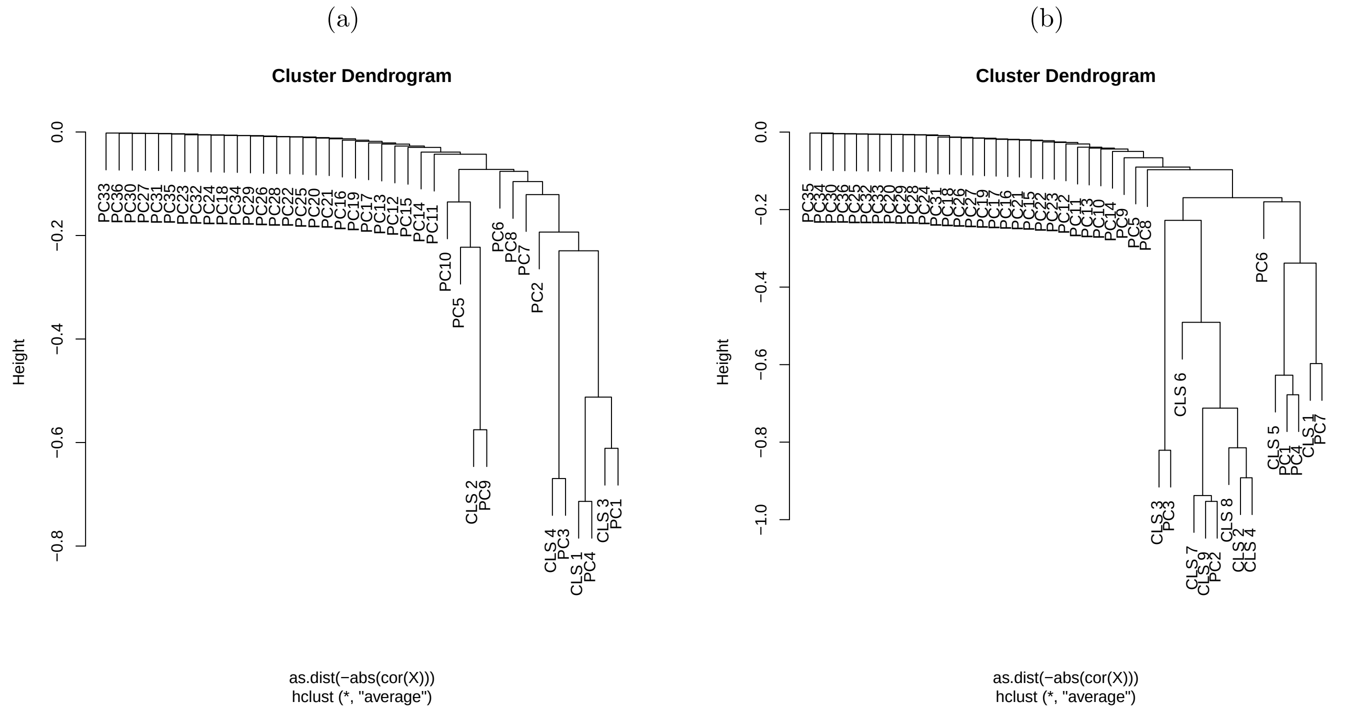

Figure S11: UPGMA of PC loadings and representative profiles of clusters obtained by WGCNA. (a) $n_c = 4$ (b) $n_c = 9$

cluster that works as a garbage box because a garbage box must include probes associated with a diversity too large to be clustered. Regarding the methodology, this is a fundamental problem that clustering cannot avoid. This is why PCA based unsupervised FE can outperform the other four clustering methods.

References

[1] Jun Yan. *som: Self-Organizing Map*, 2010. R package version 0.3-5.

[2] R Core Team. *R: A Language and Environment for Statistical Computing.* R Foundation for Statistical Computing, Vienna, Austria, 2014.

[3] P. Langfelder and S. Horvath. WGCNA: an R package for weighted correlation network analysis. *BMC Bioinformatics*, 9:559, 2008.

[4] Teuvo Kohonen. The self-organizing map. *Neurocomputing*, 21(1):1–6, 1998.

(a)

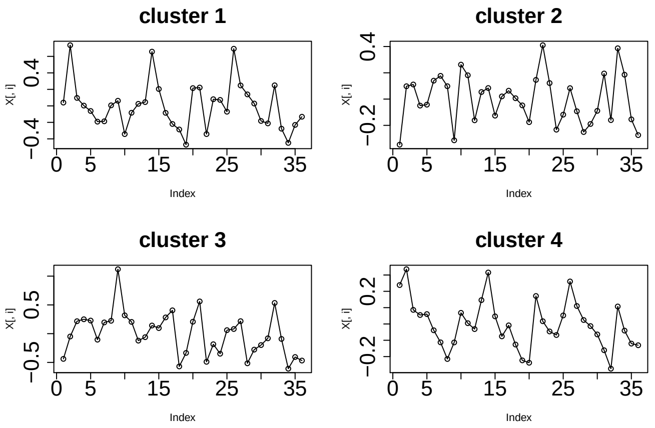

(b)

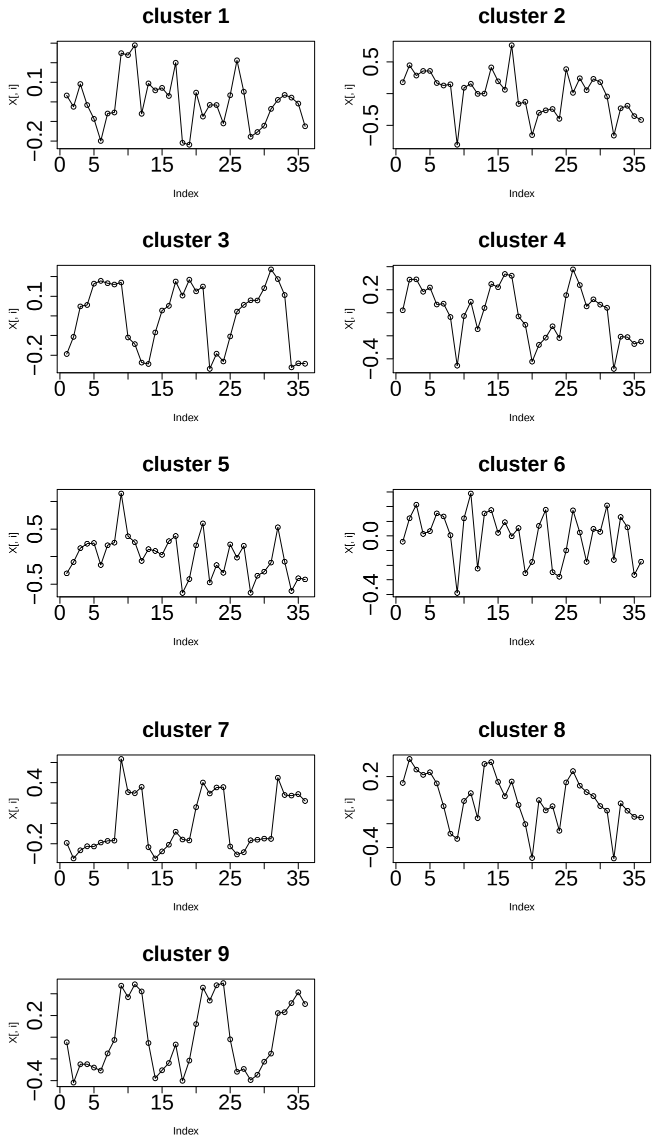

(c)

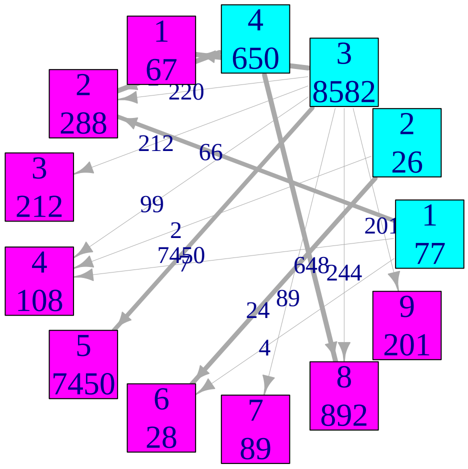

Figure S12: Representative profiles of clusters obtained by WGCNA (a) $n_c = 4$ (b) $n_c = 9$ (c) Comparison between (a) and (b). Squares filled with cyan and magenta correspond to clusters in (a) and (b), respectively. Upper numbers in squares indicate cluster number. Lower numbers in squares indicate the number of probes in each cluster. Arrows are how probes moved from clusters to clusters when n_c increases from 4 to 9. The numbers associated with arrows are the number of genes that moved between clusters; width of arrow represents the ratio of moving genes to total number of genes in each cluster.

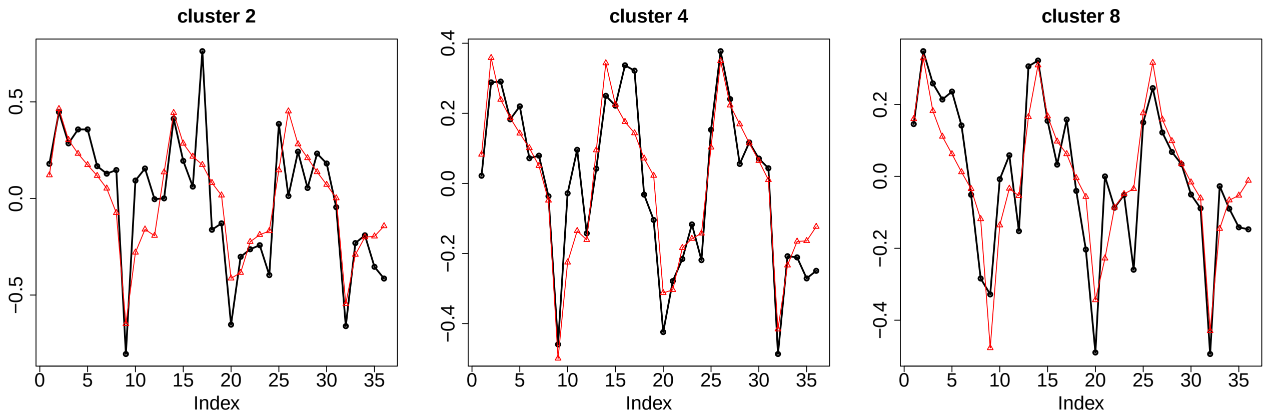

Figure S13: Regression analysis of representative profiles of clusters 4, 6 and 7 identified by WGCNA when $n_c = 9$ using PC2 and PC3 loadings. Black lines are representative profiles and red lines are regression profiles using PC2 and PC3 loadings (Table S4).
