# Supplementary material for: Principal component analysis based unsupervised feature extraction applied to budding yeast temporally periodic gene expression
Source: BioData Min. 2016 Jun 29;9:22. doi: 10.1186/s13040-016-0101-9 (PMC4928327; doi:10.1186/s13040-016-0101-9)
Supplement: Additional file 14 — Figure S4. Relationship between figures and tables. (PDF 114 kb) [file 13040_2016_101_MOESM14_ESM.pdf]

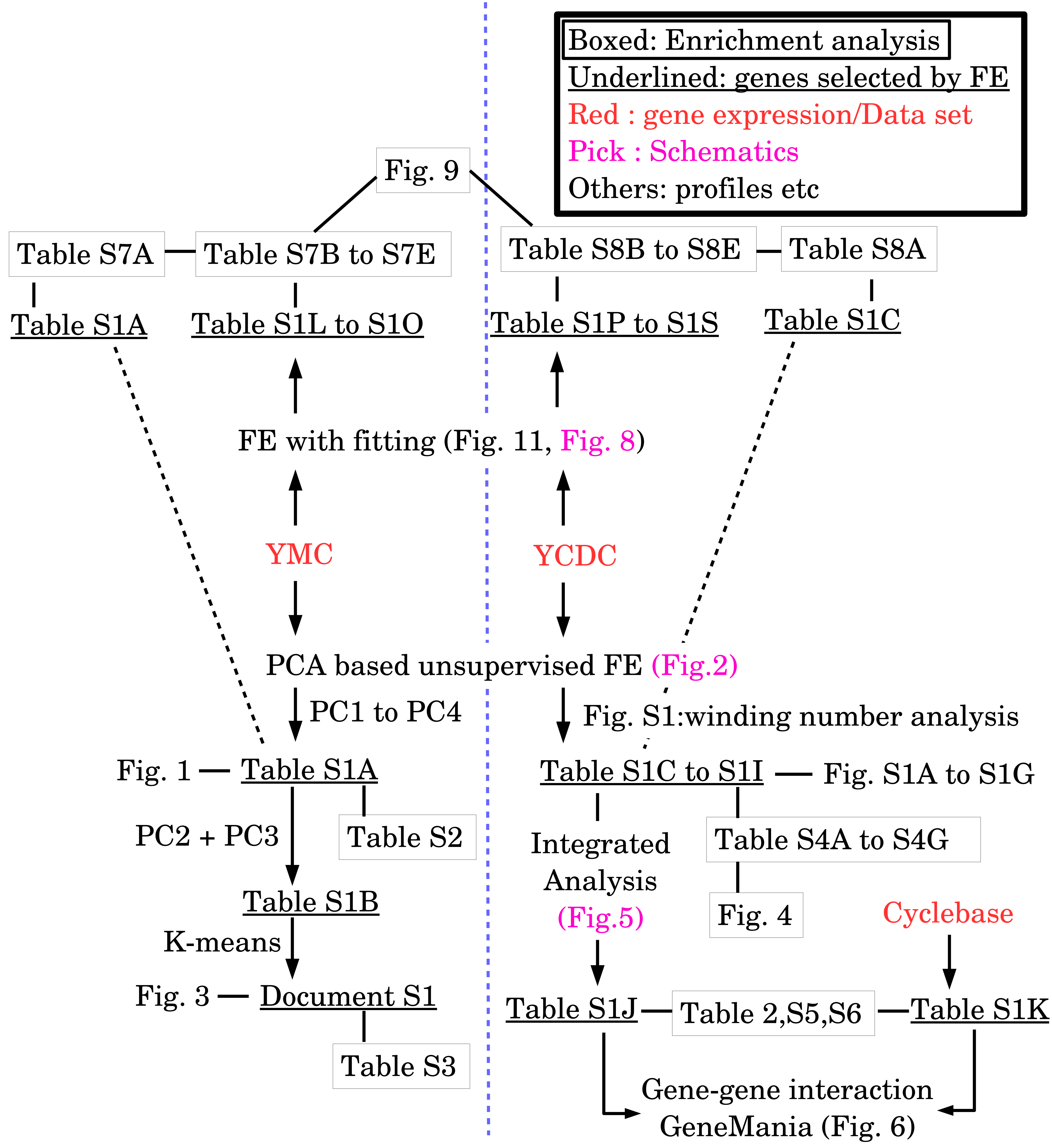
Boxed: Enrichment analysis
Underlined: genes selected by FE
Red : gene expression/Data set
Pick : Schematics
Others: profiles etc
Fig. 9
Table S7A
Table S7B to S7E
Table S8B to S8E
Table S8A
Table S1A
Table S1L to S1O
Table S1P to S1S
Table S1C
FE with fitting (Fig. 11, Fig. 8)
YMC
YCDC
PCA based unsupervised FE (Fig.2)
PC1 to PC4
Fig. S1:winding number analysis
Fig. 1
Table S1A
Table S1C to S1I
Fig. S1A to S1G
PC2 + PC3
Table S2
Integrated Analysis (Fig.5)
Table S4A to S4G
Table S1B
Fig. 4
Cyclebase
K-means
Fig. 3
Document S1
Table S1J
Table 2,S5,S6
Table S1K
Table S3
Gene-gene interaction
GeneMania (Fig. 6)
