# Supplementary material for: Principal component analysis based unsupervised feature extraction applied to budding yeast temporally periodic gene expression
Source: BioData Min. 2016 Jun 29;9:22. doi: 10.1186/s13040-016-0101-9 (PMC4928327; doi:10.1186/s13040-016-0101-9)
Supplement: Additional file 6 — Table S4. Enrichment analyses by g:profiler for the genes listed in Table S1C to S1I, each of which corresponds to A to G, respectively. (PDF 235 kb) [file 13040_2016_101_MOESM6_ESM.pdf]

source	term name Gene Ontology (Biological process)	term ID	n. of term genes	corrected p-value	A	B	C	D	E	F	G
BP	response to organic substance	GO:0010033	206	1.08e-02					16		
BP	response to pheromone	GO:0019236	107	7.44e-05				11	14		
BP	response to stimulus	GO:0050896	1148	1.15e-04		49	51	52	51	45	
BP	response to stress	GO:0006950	730	1.14e-04	36	39	41	38	34	32	
BP	single-organism process	GO:0044699	3755	4.43e-13		115	144	121	108		101
BP	cellular process	GO:0009987	4884	4.35e-07		127	155	137	125		
BP	cell wall organization or biogenesis	GO:0071554	251	2.89e-05				18	20	19	21
BP	single-organism cellular process	GO:0044763	3397	7.87e-17		114	142	117	105		96
BP	cell division	GO:0051301	355	6.69e-17	25	41	43	43	31	30	32
BP	cell cycle	GO:0007049	741	2.29e-28	43	72	75	71	56	49	51
BP	cell cycle process	GO:0022402	629	1.61e-23	35	62	64	63	46	41	45
BP	cell cycle phase transition	GO:0044770	140	8.95e-10		20	23	16		13	14
BP	cell cycle G1/S phase transition	GO:0044843	66	3.17e-11		15	18	11			11
BP	spindle organization	GO:0007051	40	3.44e-03				8			
BP	cytokinesis	GO:0000910	83	4.47e-02							9
BP	cytoskeleton-dependent cytokinesis	GO:0061640	72	1.40e-02						9	9
BP	cytokinetic process	GO:0032506	67	7.67e-03						9	9
BP	spindle pole body separation	GO:0000073	13	1.95e-04		6	6	6			
BP	mitotic cell cycle	GO:0000278	379	8.03e-22	28	48	52	47	33	31	31
BP	mitotic cell cycle process	GO:1903047	367	1.16e-20	26	46	50	45	32	29	31
BP	mitotic cytokinesis	GO:0000281	65	4.80e-02							8
BP	mitotic cell cycle phase transition	GO:0044772	137	5.56e-10		20	23	16	12	13	14
BP	G1/S transition of mitotic cell cycle	GO:0000082	66	3.17e-11		15	18	11			11
BP	regulation of transcription involved in G1/S transition of ...	GO:0000083	27	2.52e-11		10	13	8	6	7	7
BP	negative regulation of transcription involved in G1/S tr ...	GO:0071930	7	1.45e-02			4				
BP	mitotic cytokinetic process	GO:1902410	55	1.38e-02							8
BP	mitotic spindle organization	GO:0007052	34	1.17e-02				7			
BP	cellular response to stimulus	GO:0051716	983	4.98e-05		46		48	47	41	
BP	cellular response to stress	GO:0033554	631	2.35e-05		37	37	35	30	29	
BP	cellular response to DNA damage stimulus	GO:0006974	331	2.48e-08	23	30	28	30	23	23	21
BP	cell separation after cytokinesis	GO:0000920	20	2.24e-04				7		6	6
BP	multi-organism process	GO:0051704	305	6.83e-04					22		
BP	multi-organism cellular process	GO:0044764	186	3.03e-06				15	20	15	
BP	conjugation	GO:0000746	127	2.44e-08				15	19	15	
BP	regulation of gene expression, epigenetic	GO:0040029	151	2.23e-04		16	14	14			13
BP	sex determination	GO:0007530	35	2.38e-03		7	8				
BP	reproduction	GO:0000003	486	4.56e-14		38	38	43	44	35	29
BP	reproduction of a single-celled organism	GO:0032505	203	1.20e-03		15	19	16	17		
BP	meiotic cell cycle	GO:0051321	303	3.46e-03		21	21	20			
BP	meiotic cell cycle process	GO:1903046	236	2.01e-02		17		17			
BP	biological regulation	GO:0065007	1791	7.41e-06		72	73	66	63		
BP	regulation of molecular function	GO:0065009	248	1.00e-02		18					
BP	regulation of biological process	GO:0050789	1576	8.11e-05		64	69				
BP	regulation of cellular process	GO:0050794	1506	1.24e-05		64	69	58	54		
BP	regulation of cell cycle	GO:0051726	214	1.55e-07		23	25	24	19		15
BP	regulation of cell cycle process	GO:0010564	154	1.05e-02		13	15	14			
BP	regulation of spindle pole body separation	GO:0010695	9	7.01e-04		5	5	4	4	4	
BP	positive regulation of spindle pole body separation	GO:0010696	7	9.12e-03		4	4				
BP	regulation of mitotic cell cycle	GO:0007346	121	5.18e-03		12		13			
BP	regulation of metabolic process	GO:0019222	1194	9.23e-04		51					
BP	regulation of catalytic activity	GO:0050790	228	1.28e-02		17					
BP	regulation of transferase activity	GO:0051338	104	4.28e-04		13	14	11	11		
BP	regulation of nitrogen compound metabolic process	GO:0051171	796	3.12e-03		38					
BP	cellular component organization or biogenesis	GO:0071840	2397	2.18e-04				88	80		
BP	cellular component organization	GO:0016043	2045	2.33e-07		74		86	78	66	65
BP	external encapsulating structure organization	GO:0045229	212	4.73e-05	16				18	18	19
BP	cell wall organization	GO:0071555	212	4.73e-05	16				18	18	19
BP	organelle organization	GO:0006996	1491	1.87e-04		58		64	55		
BP	organelle fission	GO:0048285	351	2.44e-08	23	30	30	32	26		
BP	nuclear division	GO:0000280	341	1.12e-08	23	30	30	32	26		
BP	meiotic nuclear division	GO:0007126	170	4.85e-04		16	15	17	15		
BP	single-organism organelle organization	GO:1902589	1007	1.13e-02				45			
BP	mitotic nuclear division	GO:0007067	215	2.76e-08	18	23	24	25	19		
BP	meiosis I	GO:0007127	98	4.56e-04	11	12	11	13	12		
BP	chromosome organization	GO:0051276	523	3.36e-06	27	34	34	36	31		
BP	DNA conformation change	GO:0071103	90	8.11e-03		10		11			
BP	DNA geometric change	GO:0032392	32	5.55e-04		7		8			
BP	DNA duplex unwinding	GO:0032508	31	4.24e-04		7		8			
BP	sister chromatid cohesion	GO:0007062	49	3.33e-02			8				
BP	mitotic sister chromatid cohesion	GO:0007064	38	4.62e-03			8				
BP	regulation of cellular component organization	GO:0051128	393	5.87e-03		24	25				
BP	regulation of organelle organization	GO:0033043	290	6.59e-03		20		20			
BP	reproductive process	GO:0022414	273	2.35e-08				23	27	20	
BP	reproductive process in single-celled organism	GO:0022413	151	2.66e-02					13		
BP	multi-organism reproductive process	GO:0044703	238	4.14e-05				17	21	16	
BP	sexual reproduction	GO:0019953	238	4.14e-05				17	21	16	
BP	conjugation with cellular fusion	GO:0000747	126	2.11e-08				15	19	15	
BP	cytogamy	GO:0000755	13	6.10e-03					5		
BP	cellular response to organic substance	GO:0071310	169	4.10e-03					15		
BP	cellular response to pheromone	GO:0071444	97	1.69e-04					13		
BP	response to pheromone involved in conjugation with cellular fusion	GO:0000749	83	2.49e-05				10	13		
BP	regulation of macromolecule metabolic process	GO:0060255	1059	1.42e-04		49	50				
BP	regulation of primary metabolic process	GO:0080090	1066	1.75e-04		49	50				
BP	negative regulation of biological process	GO:0048519	554	3.66e-08		39	36	36	31		27
BP	negative regulation of cellular process	GO:0048523	527	7.71e-09		39	36	36	31		27
BP	negative regulation of cell cycle	GO:0045786	97	1.68e-02				11			
BP	negative regulation of metabolic process	GO:0009892	444	7.83e-06		31	28	27	24		23
BP	negative regulation of macromolecule metabolic process	GO:0010605	399	1.08e-05		29	26	25	24		22
BP	negative regulation of gene expression	GO:0010629	291	1.80e-03		21					18
BP	negative regulation of gene expression, epigenetic	GO:0045814	150	2.04e-04		16	14	14			13
BP	gene silencing	GO:0016458	154	2.94e-04		16	14	14			13
BP	negative regulation of nitrogen compound metabolic process	GO:0051172	309	7.20e-05		24	22	20			20
BP	regulation of protein modification process	GO:0031399	137	2.12e-03		14	15	13	13		
BP	cell fate commitment	GO:0045165	35	2.38e-03		7	8				
BP	signal transduction involved in conjugation with cellular fusion	GO:0032005	33	6.41e-03					7		
BP	negative regulation of biosynthetic process	GO:0009890	323	2.61e-03		22					19
BP	regulation of cellular metabolic process	GO:0031323	1086	1.15e-04		50	51				
BP	regulation of phosphorus metabolic process	GO:0051174	141	1.58e-02		13	14				
BP	regulation of phosphate metabolic process	GO:0019220	141	1.58e-02		13	14				
BP	regulation of nucleobase-containing compound metabolic process	GO:0019219	783	2.08e-03		38	39				
BP	regulation of RNA metabolic process	GO:0051252	722	4.31e-02		33					
BP	negative regulation of cellular metabolic process	GO:0031324	430	3.60e-06		31	28	27	24		23
BP	negative regulation of nucleobase-containing compound metabolic process	GO:0045934	304	5.26e-05		24	22	20			20
BP	negative regulation of RNA metabolic process	GO:0051253	270	5.24e-04		21					18
BP	sulfate reduction	GO:0019419	3	1.25e-02	3						
BP	sulfate assimilation, phosphoadenylyl sulfate reduction by phosphoaden ...	GO:0019379	3	1.25e-02	3						
BP	mating type determination	GO:0007531	35	2.38e-03		7	8				
BP	regulation of macromolecule biosynthetic process	GO:0010556	860	4.86e-02		37					
BP	regulation of cellular macromolecule biosynthetic process	GO:2000112	854	4.16e-02		37					
BP	negative regulation of macromolecule biosynthetic process	GO:0010558	302	8.37e-04		22					19
BP	negative regulation of cellular biosynthetic process	GO:0031327	323	2.61e-03		22					19
BP	negative regulation of cellular macromolecule biosynthetic process	GO:2000113	301	7.91e-04		22					19
BP	nucleobase-containing compound biosynthetic process	GO:0034654	966	8.95e-03		42					
BP	RNA biosynthetic process	GO:0032774	769	2.54e-02		35					
BP	regulation of RNA biosynthetic process	GO:2001141	705	2.63e-02		33					
BP	regulation of nucleic acid-templated transcription	GO:1903506	705	2.63e-02		33					
BP	regulation of transcription, DNA-templated	GO:0006355	704	2.56e-02		33					
BP	negative regulation of RNA biosynthetic process	GO:1902679	268	4.62e-04		21					18
BP	negative regulation of nucleic acid-templated transcription	GO:1903507	268	4.62e-04		21					18
BP	negative regulation of transcription, DNA-templated	GO:0045892	267	4.34e-04		21					18
BP	chromatin silencing	GO:0006342	150	2.04e-04		16	14	14			13
BP	chromatin silencing at silent mating-type cassette	GO:0030466	53	3.05e-02				8			
BP	chromatin silencing at telomere	GO:0006348	84	1.76e-02		10					
BP	phosphorylation	GO:0016310	338	1.51e-03		23		22			
BP	regulation of phosphorylation	GO:0042325	89	5.79e-05		13	14	12	11	10	
BP	regulation of kinase activity	GO:0043549	69	1.86e-05		12	13	10	10		
BP	G-protein coupled receptor signaling pathway	GO:0007186	44	4.71e-02					7		
BP	pheromone-dependent signal transduction involved in conjugation with c ...	GO:0000750	33	6.41e-03					7		
BP	DNA metabolic process	GO:0006259	542	1.86e-08	30	39	41	37	33	29	26
BP	DNA strand elongation	GO:0022616	37	8.85e-06		9		10		7	
BP	heteroduplex formation	GO:0030491	9	3.24e-02	4						
BP	regulation of DNA metabolic process	GO:0051052	107	1.74e-06		13	17	12			
BP	positive regulation of DNA metabolic process	GO:0051054	27	4.18e-03			7				
BP	DNA recombination	GO:0006310	241	3.57e-04		20	19	20	18	16	
BP	mitotic recombination	GO:0006312	58	4.45e-03		8			9		
BP	DNA replication	GO:0006260	211	1.21e-06		22	21	22		17	
BP	cell cycle DNA replication	GO:0044786	36	7.02e-05		9	7	7			
BP	nuclear DNA replication	GO:0033260	35	5.37e-05		9	7	7			
BP	DNA-dependent DNA replication	GO:0006261	122	1.72e-08		19	16	19	12	12	
BP	pre-replicative complex assembly	GO:0036388	20	3.87e-03		6					
BP	pre-replicative complex assembly involved in cell cycle DNA rep ...	GO:1902299	20	3.87e-03		6					
BP	pre-replicative complex assembly involved in nuclear cell cyc ...	GO:0006267	20	3.87e-03		6					
BP	DNA strand elongation involved in DNA replication	GO:0006271	34	3.54e-06		9		10		7	
BP	lagging strand elongation	GO:0006273	18	1.92e-03		6		6		5	
BP	DNA unwinding involved in DNA replication	GO:0006268	13	2.42e-04				6	5		
BP	regulation of DNA replication	GO:0006275	56	1.22e-02			9				
BP	DNA replication initiation	GO:0006270	53	2.54e-06		11		12			
BP	maintenance of DNA repeat elements	GO:0043570	23	5.03e-04		7	7	6		6	
BP	maintenance of DNA trinucleotide repeats	GO:0035753	8	1.94e-02				4			
BP	DNA repair	GO:0006281	284	5.44e-07	20	26	25	26	19	19	
BP	double-strand break repair	GO:0006302	109	3.46e-05		14	13	15	11		
BP	recombinational repair	GO:0000725	67	4.03e-04		9		11			
BP	double-strand break repair via homologous recombination	GO:0000724	59	1.01e-03		8		10			
BP	mating type switching	GO:0007533	29	4.97e-04		7	8	6	6		
BP	protein phosphorylation	GO:0006468	213	1.46e-06		22	20	21	19	15	
BP	regulation of protein phosphorylation	GO:0001932	77	7.37e-05		12	13	11	11	9	
BP	regulation of protein kinase activity	GO:0045859	65	8.66e-06		12	13	10	10		
BP	regulation of cyclin-dependent protein kinase activity	GO:1904029	32	1.01e-08		11	12	9	8	8	7
BP	regulation of protein serine/threonine kinase activity	GO:0071900	43	5.35e-07		11	12	9	9	8	7
BP	regulation of cyclin-dependent protein serine/threonine kinase ...	GO:0000079	32	1.01e-08		11	12	9	8	8	7
BP	meiotic mismatch repair	GO:0000710	9	1.96e-02		4	4				4
BP	polysaccharide catabolic process	GO:0000272	18	2.83e-02						5	
BP	spermine transport	GO:0000296	4	3.84e-02						3	
BP	morphogenesis checkpoint	GO:0044879	4	2.57e-04		4		4	4	3	
BP	sulfur amino acid metabolic process	GO:0000096	50	1.57e-02	8						
BP	methionine metabolic process	GO:0006555	42	4.04e-03	8						

source	term name Gene Ontology (Cellular component)	term ID	n. of term genes	corrected p-value	A	B	C	D	E	F	G
CC	protein-DNA complex	GO:0032993	61	2.78e-09	10	15	11	15	12	11	12
CC	pre-replicative complex	GO:0036387	17	1.30e-03		6		5			
CC	DNA replication preinitiation complex	GO:0031261	22	7.17e-03		6					
CC	DNA packaging complex	GO:0044815	17	1.39e-03	6			5	5	5	5
CC	DNA bending complex	GO:1990104	12	1.14e-04	6			5	5	5	5
CC	non-membrane-bounded organelle	GO:0043228	1276	1.17e-02				53			
CC	intracellular non-membrane-bounded organelle	GO:0043232	1276	1.17e-02				53			
CC	cytoskeleton	GO:0005856	233	2.33e-03				19			
CC	microtubule cytoskeleton	GO:0015630	123	3.22e-02				12			
CC	chromosome	GO:0005694	387	2.91e-09		31	31	35	26	24	
CC	nuclear lumen	GO:0031981	816	2.78e-02				38			
CC	cytoskeletal part	GO:0044430	218	8.50e-04				19			
CC	spindle	GO:0005819	100	2.25e-02				11			
CC	chromosomal part	GO:0044427	351	1.20e-07		26	27	31	23	21	
CC	cohesin complex	GO:0008278	4	4.72e-02				3	3		
CC	mitotic cohesin complex	GO:0030892	4	4.72e-02				3	3		
CC	replication fork	GO:0005657	56	4.84e-12	10	16	12	17	12	13	11
CC	replisome	GO:0030894	14	3.35e-04		6		5		5	
CC	nucleosome	GO:0000786	12	1.14e-04	6			5	5	5	5
CC	nuclear chromosome	GO:0000228	275	2.81e-11	19	28	26	32	24	22	18
CC	nuclear chromosome part	GO:0044454	248	3.13e-09	17	24	23	28	21	19	
CC	nuclear replication fork	GO:0043596	44	2.98e-11	8	14	10	15	11	11	10
CC	nuclear replisome	GO:0043601	14	3.35e-04		6		5		5	
CC	alpha DNA polymerase:primase complex	GO:0005658	4	4.93e-02		3		3			
CC	DNA replication factor A complex	GO:0005662	3	1.25e-02	3						
CC	nuclear chromatin	GO:0000790	117	4.94e-02			12				
CC	replication fork protection complex	GO:0031298	27	6.35e-06		8		9	7	6	7
CC	nuclear nucleosome	GO:0000788	9	3.96e-04	5			5	5	5	5
CC	condensed nuclear chromosome	GO:0000794	96	1.52e-02				11			
CC	nuclear cohesin complex	GO:0000798	4	4.72e-02				3	3		
CC	nuclear mitotic cohesin complex	GO:0034990	4	4.72e-02				3	3		
CC	nuclear pre-replicative complex	GO:0005656	17	1.30e-03		6		5			
CC	anchored component of membrane	GO:0031225	65	1.30e-05	10			9	12	11	9
CC	nucleus	GO:0005634	2303	1.29e-05		84					
CC	cell periphery	GO:0071944	661	1.27e-12	37		37	44	43	48	39
CC	external encapsulating structure	GO:0030312	106	3.85e-17	22			18	22	25	16
CC	cell wall	GO:0005618	106	3.85e-17	22			18	22	25	16
CC	fungal-type cell wall	GO:0009277	99	1.16e-16	21			17	20	24	15
CC	plasma membrane	GO:0005886	469	2.83e-02			28				
CC	site of polarized growth	GO:0030427	255	6.22e-09		24	24	28	24	22	19
CC	cellular bud	GO:0005933	219	1.83e-11	18	27	25	29	23	24	25
CC	cellular bud neck	GO:0005935	168	6.24e-09		22	21	23	16	16	17
CC	MCM complex	GO:0042555	6	3.98e-03		4		4			
CC	extracellular region	GO:0005576	97	7.85e-21	24		11	20	23	27	21

source	term name Gene Ontology (Molecular function)	term ID	n. of term genes	corrected p-value	A	B	C	D	E	F	G
MF	structural constituent of cell wall	GO:0005199	16	9.16e-04	6						
MF	spermine transmembrane transporter activity	GO:0000297	4	3.84e-02						3	
MF	macromolecular complex binding	GO:0044877	207	1.50e-02		16		16			
MF	chromatin binding	GO:0003682	109	3.21e-02		11					
MF	acid phosphatase activity	GO:0003993	9	2.34e-02						4	
MF	hydrolase activity, acting on glycosyl bonds	GO:0016798	57	2.54e-03						9	8
MF	hydrolase activity, hydrolyzing O-glycosyl compounds	GO:0004553	46	4.53e-02						7	
MF	molecular function regulator	GO:0098772	270	8.65e-03		19					
MF	enzyme regulator activity	GO:0030234	237	1.24e-03		19					
MF	kinase regulator activity	GO:0019207	53	8.41e-05		10	11	9	8		
MF	protein kinase regulator activity	GO:0019887	51	5.50e-05		10	11	8			
MF	cyclin-dependent protein serine/threonine kinase regulator activity	GO:0016538	26	1.58e-08	6	10	11	8	7	6	7
MF	kinase binding	GO:0019900	27	4.18e-03		6	7				
MF	protein kinase binding	GO:0019901	23	1.26e-03		6	7				
MF	DNA binding	GO:0003677	579	1.01e-04		34	32	31			
MF	sequence-specific DNA binding	GO:0043565	247	9.47e-03		18					
MF	DNA replication origin binding	GO:0003688	33	5.10e-04		8	7	8			

source	term name Biological pathways (KEGG)	term ID	n. of term genes	corrected p-value	A	B	C	D	E	F	G
ke	Base excision repair	KEGG:03410	18	4.30e-02						4	
ke	Mismatch repair	KEGG:03430	20	4.87e-05	5	7	6	6	5	6	5
ke	Cell cycle - yeast	KEGG:04111	126	1.01e-18	16	30	27	30	27	19	17
ke	Sulfur metabolism	KEGG:00920	15	1.96e-02	4						
ke	DNA replication	KEGG:03030	31	6.78e-11	5	13	8	12	8	9	7
ke	Riboflavin metabolism	KEGG:00740	14	1.54e-02						4	
ke	MAPK signaling pathway - yeast	KEGG:04011	56	4.93e-02					7		
ke	Meiosis - yeast	KEGG:04113	130	7.84e-10		22	20	20	13	12	11

source	term name Biological pathways (Reactome)	term ID	n. of term genes	corrected p-value	A	B	C	D	E	F	G
re	DNA Replication	REAC:5719403	67	2.00e-06		13		12	9	8	7
re	Cell Cycle	REAC:5719402	113	1.60e-11		21	16	19	14	15	11
re	Chromosome Maintenance	REAC:5719707	14	3.45e-03		5				4	
re	Telomere Maintenance	REAC:5719706	14	3.45e-03		5				4	
re	Extension of Telomeres	REAC:5719705	14	3.45e-03		5				4	
re	Telomere C-strand (Lagging Strand) Synthesis	REAC:5719746	12	1.43e-03		5	4	4		4	
re	Telomere C-strand synthesis initiation	REAC:5719745	6	1.09e-03		4	3	3		3	
re	Polymerase switching on the C-strand of the telomere	REAC:5719747	10	1.40e-02		4		4			
re	Cell Cycle, Mitotic	REAC:5719401	101	2.36e-11		20	15	18	14	14	11
re	S Phase	REAC:5719400	68	2.42e-06		13		12	9	8	7
re	Synthesis of DNA	REAC:5719399	67	2.00e-06		13		12	9	8	7
re	DNA strand elongation	REAC:5719415	26	7.78e-11	5	12	7	12	9	7	7
re	Unwinding of DNA	REAC:5719729	10	4.76e-04		5		5			
re	Lagging Strand Synthesis	REAC:5719416	16	1.39e-05		7	5	7	6	6	4
re	Processive synthesis on the lagging strand	REAC:5719417	11	4.48e-07	4	7	5	7	6	6	4
re	Removal of the Flap Intermediate	REAC:5719418	11	4.48e-07	4	7	5	7	6	6	4
re	Leading Strand Synthesis	REAC:5719414	10	1.40e-02		4		4			
re	Polymerase switching	REAC:5719413	10	1.40e-02		4		4			
re	DNA replication initiation	REAC:5719398	6	1.09e-03		4	3	3		3	
re	M Phase	REAC:5719693	22	3.79e-02		5	5				
re	Mitotic Prophase	REAC:5720016	11	8.53e-04	4	5	5	4		4	4
re	Nuclear Envelope Breakdown	REAC:5720063	8	4.88e-03		4	4				
re	Depolymerisation of the Nuclear Lamina	REAC:5720062	8	4.88e-03		4	4				
re	Mitotic G1-G1/S phases	REAC:5719408	21	1.40e-10	5	11	10	9	7	8	6
re	G1/S Transition	REAC:5719407	21	1.40e-10	5	11	10	9	7	8	6
re	E2F mediated regulation of DNA replication	REAC:5719680	19	1.64e-09	5	10	9	9	7	7	6
re	E2F-enabled inhibition of pre-replication complex formation	REAC:5719681	8	4.88e-03		4	4				
re	Inhibition of replication initiation of damaged DNA by RB1/E2F1	REAC:5719679	4	1.04e-02		3		3			
re	G1/S-Specific Transcription	REAC:5719922	9	3.97e-03		4	4	4	4		
re	Mitotic G2-G2/M phases	REAC:5719696	14	1.43e-04	4	6	6	5	4	5	4
re	G2/M Transition	REAC:5719695	14	1.43e-04	4	6	6	5	4	5	4
re	Cyclin A/B1 associated events during G2/M transition	REAC:5719730	14	1.43e-04	4	6	6	5	4	5	4
re	Cyclin B2 mediated events	REAC:5719732	8	4.88e-03		4	4				
re	Regulation of mitotic cell cycle	REAC:5719736	9	8.59e-03		4	4				
re	APC/C-mediated degradation of cell cycle proteins	REAC:5719735	9	8.59e-03		4	4				
re	Regulation of APC/C activators between G1/S and early anaphase	REAC:5719737	8	4.88e-03		4	4				
re	M/G1 Transition	REAC:5719406	6	1.09e-03		4	3	3		3	
re	DNA Replication Pre-Initiation	REAC:5719405	6	1.09e-03		4	3	3		3	
re	Activation of the pre-replicative complex	REAC:5719404	6	1.09e-03		4	3	3		3	
re	G2/M Checkpoints	REAC:5719425	14	1.43e-04		6	6	5		5	
re	G2/M DNA replication checkpoint	REAC:5719731	8	1.11e-04		5	5	4		4	
re	Attenuation phase	REAC:5720061	6	3.42e-02	3		3				
re	Mismatch Repair	REAC:5720068	8	1.36e-03		4	4				4
re	Mismatch repair (MMR) directed by MSH2:MSH3 (MutSbeta)	REAC:5720069	7	3.42e-02							3
re	Mismatch repair (MMR) directed by MSH2:MSH6 (MutSalpha)	REAC:5720067	7	6.92e-04		4	4		3	3	4

source	term name Regulatory motifs in DNA (TRANSFAC TFBS)	term ID	n. of term genes	corrected p-value	A	B	C	D	E	F	G
tf	Factor: HSF; motif: AGAANNTTCTAGAAN; match class: 3	TF:M00169_3	264	1.30e-02	15						
tf	Factor: XBP1; motif: CTTCGAG; match class: 0	TF:M00683_0	395	3.49e-02			20				
tf	Factor: HSF; motif: AGAANNTTCTAGAAN; match class: 0	TF:M00169_0	42	7.12e-03	6						
tf	Factor: MCM1+SFF; motif: TTWCCYAAWNNGGAAAWWNGTAAAYAA; match class: 4	TF:M01051_4	26	3.12e-06	5	6	6	8	7	7	6
tf	Factor: MCM1+SFF; motif: TTWCCYAAWNNGGAAAWWNGTAAAYAA; match class: 3	TF:M01051_3	19	1.17e-04	4	5	5	6	5	5	4
tf	Factor: MCM1+SFF; motif: TTWCCYAAWNNGGAAAWWNGTAAAYAA; match class: 2	TF:M01051_2	9	6.61e-04		4	3	3	3	3	
tf	Factor: MCM1; motif: WTTCCYAAWNNGGTAA; match class: 4	TF:M00125_4	198	1.57e-17	21	29	28	31	31	27	22
tf	Factor: MCM1; motif: WTTCCYAAWNNGGTAA; match class: 3	TF:M00125_3	161	8.97e-16	19	24	23	28	26	22	17
tf	Factor: MCM1; motif: WTTCCYAAWNNGGTAA; match class: 2	TF:M00125_2	82	1.80e-08	10	14	12	15	13	10	9
