# Supplementary material for: Principal component analysis based unsupervised feature extraction applied to budding yeast temporally periodic gene expression
Source: BioData Min. 2016 Jun 29;9:22. doi: 10.1186/s13040-016-0101-9 (PMC4928327; doi:10.1186/s13040-016-0101-9)
Supplement: Additional file 15 — Reviewer reports and AU response to reviewers. (DOCX 35.8 kb) [file 13040_2016_101_MOESM15_ESM.docx]

**BIDM-D-15-00024
"Principal component analysis based unsupervised feature extraction applied to budding yeast temporally periodic gene expression"
Original Submission

Alison Motsinger-Reif (Reviewer 1)**

[Edit Reviewer Comments](javascript:open_edit_reviewer_comments())

| **Reviewer Recommendation Term:** | Reject |
| --- | --- |
| **Rate Review:** | Please enter a number from 1-100 to rate this review (100 is best) |
| **Custom Review Question(s)** \| **Response**; **Level of interest** Please indicate how interesting you found the manuscript: \| An article of limited interest; **Quality of written English** Please indicate the quality of language in the manuscript: \| Needs some language corrections before being published; **Declaration of competing interests** Please complete a declaration of competing interests, considering the following questions: 1. Have you in the past five years received reimbursements, fees, funding, or salary from an organisation that may in any way gain or lose financially from the publication of this manuscript, either now or in the future? 2. Do you hold any stocks or shares in an organisation that may in any way gain or lose financially from the publication of this manuscript, either now or in the future? 3. Do you hold or are you currently applying for any patents relating to the content of the manuscript? 4. Have you received reimbursements, fees, funding, or salary from an organization that holds or has applied for patents relating to the content of the manuscript? 5. Do you have any other financial competing interests? 6. Do you have any non-financial competing interests in relation to this paper? If you can answer no to all of the above, write 'I declare that I have no competing interests' below. If your reply is yes to any, please give details below. \| I declare that I have no competing interests.; I agree to the open peer review policy of the journal. I understand that my name will be included on my report to the authors and, if the manuscript is accepted for publication, my named report including any attachments I upload will be posted on the website along with the authors' responses. I agree for my report to be made available under an Open Access Creative Commons CC-BY license (http://creativecommons.org/licenses/by/4.0/). I understand that any comments which I do not wish to be included in my named report can be included as confidential comments to the editors, which will not be published. \| I agree to the open peer review policy of the journal **Comments to Editor:**; This study is of very limited interest.; **Comments to Author:**; The enclosed manuscript described an evaluation of principal components based feature extraction for pathway analysis in budding yeast. The author compares supervised versus unsupervised results, and demonstrate improved performance of the unsupervised approach. While this is an interesting topic, there are a number of concerns with the study and the manuscript. First, there are major concerns with comparing methods entirely on real data. While the data they use is well studied, true measures of model performance cannot be evaluated with real data. This can be done with extensive functional validation, but there is limited discussion on this in the study. Because of this, the results have limited applicability outside of the dataset outside of the immediate dataset used. On a more minor note, there are a number of minor concerns with the technical details in the manuscript. For example, they present the results of a hypothesis test as p = 0, which is not technically possible. Additionally, the figures are extremely complicated, making it difficult to follow the results. **Transfer Authorization** \| **Response**; If this submission is transferred to another publication, do we have your consent to include your identifying information? \| No; If this submission is transferred to another publication, do we have your consent to include your original review? \| Yes | |

**BIDM-D-15-00024
"Principal component analysis based unsupervised feature extraction applied to budding yeast temporally periodic gene expression"
Original Submission

Najaf Shah (Reviewer 2)**

[Edit Reviewer Comments](javascript:open_edit_reviewer_comments())

| **Reviewer Recommendation Term:** | Major Revision |
| --- | --- |
| **Rate Review:** | Please enter a number from 1-100 to rate this review (100 is best) |
| **Custom Review Question(s)** \| **Response**; **Level of interest** Please indicate how interesting you found the manuscript: \| An article of limited interest; **Quality of written English** Please indicate the quality of language in the manuscript: \| Not suitable for publication unless extensively edited; **Declaration of competing interests** Please complete a declaration of competing interests, considering the following questions: 1. Have you in the past five years received reimbursements, fees, funding, or salary from an organisation that may in any way gain or lose financially from the publication of this manuscript, either now or in the future? 2. Do you hold any stocks or shares in an organisation that may in any way gain or lose financially from the publication of this manuscript, either now or in the future? 3. Do you hold or are you currently applying for any patents relating to the content of the manuscript? 4. Have you received reimbursements, fees, funding, or salary from an organization that holds or has applied for patents relating to the content of the manuscript? 5. Do you have any other financial competing interests? 6. Do you have any non-financial competing interests in relation to this paper? If you can answer no to all of the above, write 'I declare that I have no competing interests' below. If your reply is yes to any, please give details below. \| None; I agree to the open peer review policy of the journal. I understand that my name will be included on my report to the authors and, if the manuscript is accepted for publication, my named report including any attachments I upload will be posted on the website along with the authors' responses. I agree for my report to be made available under an Open Access Creative Commons CC-BY license (http://creativecommons.org/licenses/by/4.0/). I understand that any comments which I do not wish to be included in my named report can be included as confidential comments to the editors, which will not be published. \| I agree to the open peer review policy of the journal **Comments to Editor:**; Summary ------- In this manuscript, the authors describe the application of a method they have previously published for identifying candidate genes associated with a particular biological process. The name of the method is PCA-based unsupervised feature extraction, and the authors have previously applied it to miRNA datasets in a paper published in BMC bioinformatics. The method is meant to be generally applicable to the common situation in biology where there is a large number of features (e.g. genes) and a small number of samples (e.g. cell types). The goal of the current manuscript is to apply the same general method to datasets on the yeast metabolic cycle, and the yeast cell division cycle. Authors state that one key aspect of the two chosen datasets is that both are temporal. Authors state that in the past, such datasets have been analyzed by first fitting to sinusoidal functions, and then processing further to identify candidate genes associated with the cell cycle, etc depending on the dataset. However, the authors' method achieves superior enrichment by making no assumptions on the periodicity of the expression profiles (e.g. the method was able to identify profiles with periods about half of the length of the cell cycle). The authors claim that this is the first demonstration of a method that is able to identify temporally-regulated genes via an unsupervised method. The authors first apply the method to metabolic cycle data. They then take the set of enriched genes output by the method, and run it through a Gene Ontology-term enrichment tool, which returned a number of terms under the "metabolic process" umbrella. Tests on the cellular component ontology yielded similar results suggesting success. The authors take this to mean that the method is successful in identifying the key genes in this process. Next, the authors compare their analysis to the one by Tu et al. Specifically, applying the method to only PC2 and PC3, the authors demonstrate good separation of the enriched genes into three classes, as shown by Tu et al. Simular to the usage of GO-term enrichment, the authors used the STRING server to show that there is significant enrichment of protein-protein interactions in their identified set of genes. The authors then performed basic network analysis on the protein-protein interaction networks and observe that the results in concordance to the power law. Next, the authors apply the same approach to the cell disivion cycle, and receive similar results in that their GO-term enrichment and PPI analysis yield results suggestive of having identified genes that are likely to be involved in the cell division process. Additonally, the authors use a list of 37 common genes from across the 7+ cell division experiments as input into the GO-enrichment program. They compare this with the enrichment resulting from the top 36 genes in cyclebase. Again, the results from GO-term enrichment suggest superiority of the authors' unsupervised method. The authors end with a discussion on why their method seems to outperform the method that incorporates sinusoidal fitting. To study this question, they performed fitting of the cell cycle data to sinusoid functions, and compared to the results obtained with their own method. They find that the unsupervised FE method is able to yield important GO terms that the supervised/fitting method does not. They argue that the key is in the unsupervised method's projection-based approach, as opposed to the supervised method's correlation-based approach. General comments ---------------- - I found the text quite dense and hard to follow. I understand that the authors have already published the original method, and want to avoid re-hashing it in the current manuscript. However, it was very difficult for someone like myself to follow the overall logic of the method. Skimming the BMC Bioinformatics paper helped, but should not be a requirement to understand the current manuscript. This manuscript will benefit from a simple diagram or flowchart describing the steps in the actual method. For instance, in the current format, the reader may miss that PCA is not being applied in the way it is traditionally in gene expression studies. The flowchart should clearly show how one starts from a gene expression matrix, and ends up with a set of enriched genes - Although GO-term enrichment as a means to assess a method's utility is (or was) common, it would be more powerful to use actual biological results. Perhaps an experiment where a gene expression screen was done first, and then followed-up with more targeted experiments - I'm not sure how to interpret the k-means analysis presented in Figure 2, given that the value for k is input based on the result from Tu et al. Furthermore, I understand that the genes in the three classes yielded significant GO terms, but shouldn't the actual gene lists be compared with Tu et al's results to see what the extent of overlap is? - I would advise removing the reference to power-law distribution of the identified protein-protein interaction networks. Showing that a particular biological network is scale-free is not by itself very meaningful. If the network itself was extracted by BioGRID, I don't understand how it serves as a test of the method - The last subsection in Results needs to be simplified and expanded to clearly lay out what exactly was done. The authors reference a list of 37 genes that is the intersection of genes found in the 7+ experiments. Is each experiment analyzed separately? I.e. does each of the 7+ experiments itself have multiple time-points? Again, a simple diagram would be helpful here. Would also be useful to summarize how cyclebase comes up with its own ranking of genes for readers (like myself) who have not used it before. Additionally, is stronger GO-term enrichment a good proxy for the utility of the method? - The discussion section is a critical part of the paper, but is hard to follow. The language should be simplified, and yet again, a diagram of what exactly they are comparing to their own method would be helpful. The paper in general is heavy on results and light on describing the approach Minor comments -------------- - Limit cycle is sometimes referred to as "limited cycle" - Power law is mistyped as power "low" in one place Note ---- - Apologies for being late with this review. One thing I'd like to mention is that I was not aware that the reviews themselves would be published online. Given the dense style of this paper, and more importantly, that I am not an expert on the methods that the authors test, I request that my review not be published. I'm happy to have the authors look at my review as coming from a biologist with quantitative faculty -- but should not be interpreted as a review from a person who is deep into this field. Sorry again for the inconvenience this may cause.; **Comments to Author:** **Transfer Authorization** \| **Response**; If this submission is transferred to another publication, do we have your consent to include your identifying information? \| No; If this submission is transferred to another publication, do we have your consent to include your original review? \| Yes | |

**Author's Response To Reviewer Comments**

Reviewer #1:
The enclosed manuscript described an evaluation of principal components based feature extraction for pathway analysis in budding
yeast. The author compares supervised versus unsupervised results, and demonstrate improved performance of the unsupervised
approach. While this is an interesting topic, there are a number of concerns with the study and the manuscript.
First, there are major concerns with comparing methods entirely on real data. While the data they use is well studied, true measures of
model performance cannot be evaluated with real data. This can be done with extensive functional validation, but there is limited
discussion on this in the study.

>Thank you very much for your valuable comments. We demonstrated our performance using synthetic data sets.

Because of this, the results have limited applicability outside of the dataset outside of the immediate
dataset used.

>We have applied our methodology to various biological/genomic problems and published more than 10 papers, some of which were published by BMC. Thus, the good performances of this methodology was proven outside of the immediate dataset used.

On a more minor note, there are a number of minor concerns with the technical details in the manuscript. For example, they present
the results of a hypothesis test as p = 0, which is not technically possible. Additionally, the figures are extremely complicated, making it
difficult to follow the results.

> P=0 was provided by STRING server. Thus, we are not sure why it shows not exact P value but 0.

Reviewer #2:
Summary
------
In this manuscript, the authors describe the application of a method they have previously published for identifying candidate genes
associated with a particular biological process. The name of the method is PCA-based unsupervised feature extraction, and the
authors have previously applied it to miRNA datasets in a paper published in BMC bioinformatics. The method is meant to be generally
applicable to the common situation in biology where there is a large number of features (e.g. genes) and a small number of samples
(e.g. cell types). The goal of the current manuscript is to apply the same general method to datasets on the yeast metabolic cycle, and
the yeast cell division cycle.
Authors state that one key aspect of the two chosen datasets is that both are temporal. Authors state that in the past, such datasets
have been analyzed by first fitting to sinusoidal functions, and then processing further to identify candidate genes associated with the
cell cycle, etc depending on the dataset. However, the authors' method achieves superior enrichment by making no assumptions on
the periodicity of the expression profiles (e.g. the method was able to identify profiles with periods about half of the length of the cell
cycle). The authors claim that this is the first demonstration of a method that is able to identify temporally-regulated genes via an
unsupervised method.
The authors first apply the method to metabolic cycle data. They then take the set of enriched genes output by the method, and run it
through a Gene Ontology-term enrichment tool, which returned a number of terms under the "metabolic process" umbrella. Tests on
the cellular component ontology yielded similar results suggesting success. The authors take this to mean that the method is
successful in identifying the key genes in this process. Next, the authors compare their analysis to the one by Tu et al. Specifically,
applying the method to only PC2 and PC3, the authors demonstrate good separation of the enriched genes into three classes, as
shown by Tu et al. Simular to the usage of GO-term enrichment, the authors used the STRING server to show that there is significant
enrichment of protein-protein interactions in their identified set of genes. The authors then performed basic network analysis on the
protein-protein interaction networks and observe that the results in
concordance to the power law.
Next, the authors apply the same approach to the cell disivion cycle, and receive similar results in that their GO-term enrichment and
PPI analysis yield results suggestive of having identified genes that are likely to be involved in the cell division process. Additonally,
the authors use a list of 37 common genes from across the 7+ cell division experiments as input into the GO-enrichment program.
They compare this with the enrichment resulting from the top 36 genes in cyclebase. Again, the results from GO-term enrichment
suggest superiority of the authors' unsupervised method.
The authors end with a discussion on why their method seems to outperform the method that incorporates sinusoidal fitting. To study
this question, they performed fitting of the cell cycle data to sinusoid functions, and compared to the results obtained with their own
method. They find that the unsupervised FE method is able to yield important GO terms that the supervised/fitting method does not.
They argue that the key is in the unsupervised method's projection-based approach, as opposed to the supervised method's
correlation-based approach.
General comments
-------

--I found the text quite dense and hard to follow. I understand that the authors have already published the original method, and want to
avoid re-hashing it in the current manuscript. However, it was very difficult for someone like myself to follow the overall logic of the
method. Skimming the BMC Bioinformatics paper helped, but should not be a requirement to understand the current manuscript. This
manuscript will benefit from a simple diagram or flowchart describing the steps in the actual method.

> Thank you very much for your suggestions. We added some diagram to explain what we have done.

For instance, in the current
format, the reader may miss that PCA is not being applied in the way it is traditionally in gene expression studies. The flowchart should
clearly show how one starts from a gene expression matrix, and ends up with a set of enriched genes

> As above, we have added diagram

--Although GO-term enrichment as a means to assess a method's utility is (or was) common, it would be more powerful to use actual
biological results. Perhaps an experiment where a gene expression screen was done first, and then followed-up with more targeted
experiments

> Since we do not have any facilities to perform experiments and this journal is aiming to publish the bioinformatic research, we did not do any experiments. In stead, we add additional GeneManina analysis that also confirm the usefulness of our methodology.

--I'm not sure how to interpret the k-means analysis presented in Figure 2, given that the value for k is input based on the result from
Tu et al.

> We have confirmed the reliability of k=3 using Gaussian mixture model.

Furthermore, I understand that the genes in the three classes yielded significant GO terms, but shouldn't the actual gene lists
be compared with Tu et al's results to see what the extent of overlap is?

> Since Tu et al did not provide full list of genes, we could not do this.

--I would advise removing the reference to power-law distribution of the identified protein-protein interaction networks. Showing that a
particular biological network is scale-free is not by itself very meaningful. If the network itself was extracted by BioGRID, I don't
understand how it serves as a test of the method

> We have removed them.

--The last subsection in Results needs to be simplified and expanded to clearly lay out what exactly was done.

>We have divided section to subsections for readability.

The authors reference a
list of 37 genes that is the intersection of genes found in the 7+ experiments. Is each experiment analyzed separately? I.e. does each
of the 7+ experiments itself have multiple time-points? Again, a simple diagram would be helpful here.

> We added some diagrams related to this issue.

Would also be useful to
summarize how cyclebase comes up with its own ranking of genes for readers (like myself) who have not used it before.

> We have added some sentences “ which ranks genes based upon both periodicity and the amplitude of gene expression”

Additionally, is stronger GO-term enrichment a good proxy for the utility of the method?

> It is generally believed that stronger GO-term enrichment is a good proxy for the utility of the method, I believe.

--The discussion section is a critical part of the paper, but is hard to follow. The language should be simplified, and yet again, a
diagram of what exactly they are comparing to their own method would be helpful.

>We have added some diagrams.

The paper in general is heavy on results and light
on describing the approach

>We have added many diagrams and some mathematical details on our methodology.

Minor comments
-----
--Limit cycle is sometimes referred to as "limited cycle"
--Power law is mistyped as power "low" in one place

> These were corrected.
